# Ageing and non-liver comorbidities in population with chronic hepatitis B infection in the western pacific region from 1990 to 2019

**DOI:** 10.3389/fphys.2023.1176113

**Published:** 2023-05-18

**Authors:** Jinzhao Xie, Xu Wang, Deng Pan, Jiaye Liu, Jinghua Li, Jing Gu

**Affiliations:** ^1^ Department of Medical Statistics, School of Public Health, Sun Yat-sen University, Guangzhou, China; ^2^ School of Public Health, Shenzhen University Medical School, Shenzhen, China; ^3^ Sun Yat-sen Global Health Institute, School of Public Health and Institute of State Governance, Sun Yat-sen University, Guangzhou, China; ^4^ Key Laboratory of Health Informatics of Guangdong Province, Sun Yat-sen University, Guangzhou, China

**Keywords:** hepatitis B virus, ageing, noncommunicable diseases, western pacific region, comorbidity

## Abstract

**Objectives:** This study examined the age structure and burden of non-liver noncommunicable diseases in population with chronic hepatitis B virus (HBV) infection in the Western Pacific Region (WPR) from 1990 to 2019.

**Methods:** We estimated ageing trends and the prevalence of non-liver NCDs among the HBV-infected population and the general population in 31 countries/areas in the Western Pacific Region from 1990 to 2019 based on the Global Burden of Disease 2019 dataset.

**Results:** The proportion of individuals aged 60 or older among the HBV-infected population has increased at a faster rate compared to the general population, whereas the proportion of individuals younger than 19 years has decreased rapidly over the past three decades. Among the HBV-infected population, the prevalence of most (29/31) NCDs increased from 1990 to 2019, with the top three most significant increases found for non-Hodgkin’s lymphoma (789.94% increase), prostate cancer (512.40% increase), and kidney cancer (411.34% increase). The prevalence of NCDs among the HBV-infected population increased faster than in the general population over the past three decades, especially in countries with rapid population ageing.

**Conclusion:** This study highlights the increasing burden of non-liver comorbidities among the HBV-infected population. The integrated management of non-liver NCDs among this population should be implemented.

## Introduction

In 2019, approximately 296 million people globally had a hepatitis B virus (HBV) infection (World Health Organization, 2022). To achieve the goal of eliminating hepatitis B by 2030, the World Health Organization (WHO) has implemented a series of prevention and treatment strategies, such as increasing the coverage of the hepatitis B vaccine (HepB), which has prevented 210 million incident cases of HBV infection as of 2015 (Nayagam et al., 2016; World Health Organization, 2016). The availability of long-term suppressive treatment with effective and well-tolerated oral anti-HBV drugs has increased the life expectancy of people infected with HBV, but there is still no cure (Cox and Tillmann, 2011; Kim et al., 2011). The decrease in new infections and the prolongation of the lifespan of these patients has accelerated the population ageing of HBV-infected individuals (Kemp et al., 2019; Nguyen et al., 2019).

The ageing of HBV-infected individuals increases their vulnerability to age-associated noncommunicable diseases (NCDs). A large longitudinal study conducted in Hong Kong showed that the mean age of chronic HBV patients increased from 41 years in 2000–2004 to 55 years in 2014–2017, and the prevalence of several comorbidities, such as hypertension (25.5%–28.6%), diabetes mellitus (DM) (10.6%–20.1%), and cardiovascular diseases (12.5%–22.2%), increased significantly from 2000 to 2017 (Wong et al., 2020). A recent cohort study in a North American population also found that the HBV-infected population is ageing, and the prevalence of chronic kidney disease (CKD) increased from 36.1/1,000 in 2006 to 97.6/1,000 in 2015 in this population (Nguyen et al., 2019). Furthermore, previous studies have indicated that people infected with HBV have a higher risk of developing specific non-liver NCDs than those without HBV infection because of HBV-induced liver damage and persistent inflammatory activities, anti-HBV medication-induced adverse effects, and HBV replication in non-liver histocytes (Tang et al., 2018; Song et al., 2019). For example, two multi-country meta-analyses estimated that individuals infected with HBV had an approximately 1.33- and 1.41-fold increased risk of developing DM and CKD, respectively, compared to those without HBV (Cai et al., 2015; Fabrizi et al., 2019). Another meta-analysis of 53 studies found significant positive associations between HBV infection and the development of non-liver cancers such as oesophageal cancer [relative risk (RR) = 1.32], stomach cancer (RR = 1.46), and pancreatic cancer (RR = 1.37) (Tian et al., 2020). Song et al. also found a higher risk of stomach cancer and pancreatic cancer among the HBV-infected population, as well as HBV expression in stomach and pancreatic cancer tissue (Song et al., 2019). Ageing and the extra risk induced by HBV infection together increase the risk of developing non-liver comorbidities among the HBV-infected population.

The Western Pacific Region (WPR), one of the six WHO regions, had high HBV endemicity, with an HBV prevalence of more than 8% in 1990 (Wiesen et al., 2016). However, over the past three decades, the WPR has achieved considerable progress towards regional control of HBV, particularly the elimination of HBV mother-to-child transmission (MTCT). During 2005–2017, the regional coverage of the HepB birth dose increased from 63% to 85% in the WPR (Woodring et al., 2019). By 2019, 21 of the 37 countries/areas in the WPR had achieved the regional target of <1% hepatitis B surface antigen (HBsAg) prevalence among children under 5 years of age (World Health Organization, 2021). Despite the success of these prevention strategies, 116 million people in the WPR were living with chronic HBV infection in 2019, accounting for 40% of HBV cases worldwide (World Health Organization, 2022). The HBV burden has markedly varied among countries/areas within the WPR; for example, the HBV prevalence has generally been less than 2% in low-endemicity countries in 2019 (e.g., Australia and Japan) but greater than 8% in high-endemicity countries (e.g., Laos and Papua New Guinea) (Schweitzer et al., 2015; World Health Organization, 2021). Additionally, countries in the WPR substantially vary in their population age structure, socioeconomic development, and healthcare system. Given the large number of HBV infections, the long-term management and treatment of individuals with chronic HBV infection will be a major challenge, especially for more clinically complex patients, such as those with non-liver comorbidities (Schweitzer et al., 2015). Previous studies conducted in countries/areas with rapid population ageing (e.g., China, Hong Kong, and South Korea) have shown an increasing burden of non-liver NCDs among people infected with HBV (Song et al., 2019; Oh et al., 2020; Tian et al., 2020; Wong et al., 2020). However, these studies have been limited by their sample representativeness and short observational period, and there is little evidence from countries with a younger population structure (e.g., Laos and Vietnam). To the best of our knowledge, no studies have comprehensively investigated the burden and trend of non-liver NCDs among the HBV-infected population in the WPR from 1990 to 2019.

In the current study, we estimated the prevalence of 31 non-liver-related NCDs among the HBV-infected population in the WPR based on the Global Burden of Disease (GBD) 2019 database. First, we examined the age composition among the population with HBV infection from 1990 to 2019 and compared it with that of the general population. We then calculated the prevalence of non-liver-related comorbidities among the HBV-infected population in the past three decades. Our results describe the ageing of the HBV-infected population among different countries/areas in the WPR and the burden of non-liver comorbidities in this population.

## Methods

### Data sources

The data were obtained from the GBD 2019 study database, which estimated the global burden of 369 diseases and injuries across 204 countries and territories from 1990 to 2019 and was constructed by the Institute for Health Metrics and Evaluation (Vos et al., 2020). Details of the GBD 2019 database and its metrics, data sources, and general methodology are reported elsewhere (Vos et al., 2020). Schmit et al. found that the estimates of the global prevalence of HBV infection in the GBD database were comparable to those in the WHO dashboard, the CDA/Polaris Observatory, and a systematic review conducted by Schweitzer et al. (Schmit et al., 2021). We used the prevalence of ‘Cirrhosis and other chronic liver disease due to hepatitis B’ estimated in the GBD database to represent chronic HBV infection prevalence in accordance with Schmit et al. (Schmit et al., 2021). The GBD database contains 31 of the 37 countries/areas in the WPR as defined by WHO (see Supplementary Table S1). We evaluated all 31 countries/areas in the WPR included in the GBD database. The list of these countries/areas is provided in Supplementary Table S1. We extracted the number of annual all-age and age-specific chronic HBV infection cases, the prevalence rates of HBV infection, and the age-standardised infection rates in all of the 31 WPR countries/areas contained in the GBD database for the years 1990–2019 using the Global Health Data Exchange query tool (https://ghdx.healthdata.org/gbd-2019). The age-standardised rates in the GBD database were estimated based on the world population estimates in the GBD database. We also extracted the pooled disease burden for the WPR contained in the GBD database.

To examine comorbidities among the HBV-infected population, we selected NCDs that have been investigated in previous studies on comorbidities associated with HBV. We also included common NCDs contained in the GBD database. We excluded NCDs related to the liver (e.g., liver cancer and cirrhosis). In total, 31 of the 102 NCDs covered in the GBD database were included in our study (Table 1). We extracted the all-age and age-specific prevalence rates of the selected NCDs in all of the 31 countries/areas in the WPR for the years 1990–2019 from the GBD database.

**TABLE 1:** Prevalence of noncommunicable diseases among population with hepatitis B virus infection in the western pacific region, 1990–2019.

Noncommunicable diseases	Prevalence							Relative change, 1990–2019 (%)
	1990	1995	2000	2005	2010	2015	2019	
Neoplasms/Cancer (per 100,000 persons)								
Lip and oral cavity cancer	5.20	6.09	7.23	9.71	13.54	16.33	18.93	264.04
Colon and rectum cancer	65.73	80.08	98.83	147.70	211.49	260.24	329.99	402.04
Stomach cancer	66.96	68.98	75.31	101.79	119.66	127.04	149.15	122.74
Pancreatic cancer	3.42	3.90	4.70	6.47	8.55	10.17	12.42	263.16
Non-Hodgkin’s lymphoma	11.53	16.74	22.75	34.49	61.64	82.91	102.61	789.94
Gallbladder and biliary tract cancer	2.50	2.70	3.02	4.16	4.96	5.35	6.11	144.40
Kidney cancer	5.82	6.86	9.21	14.70	20.93	24.50	29.76	411.34
Ovarian cancer	6.82	8.37	10.00	12.19	14.93	17.32	20.39	198.97
Cervical cancer	20.28	22.07	26.59	35.42	43.27	49.60	53.51	163.86
Breast cancer	96.17	109.87	136.01	179.99	242.63	294.08	357.60	271.84
Oesophageal cancer	22.69	25.04	29.97	39.60	40.16	39.26	46.45	104.72
Tracheal, bronchus and lung cancer	27.07	31.30	38.33	52.43	69.64	82.80	101.06	273.33
Prostate cancer	21.53	28.70	38.90	60.74	85.11	105.29	131.85	512.40
Thyroid cancer	10.18	12.68	16.10	22.15	30.69	32.41	37.07	264.15
Leukaemia	91.30	87.71	81.64	67.35	73.05	83.58	99.90	9.42
Multiple myeloma	2.41	2.85	3.51	4.69	6.23	7.53	9.11	278.01
Cardiovascular diseases (%)								
Stroke	1.08	1.17	1.29	1.45	1.71	2.05	2.45	125.48
Ischemic heart disease	1.46	1.62	1.84	2.22	2.61	3.11	3.61	146.98
Chronic respiratory diseases (%)								
Chronic obstructive pulmonary disease	2.22	2.45	2.62	2.91	3.08	3.43	3.73	68.12
Asthma	3.07	2.99	2.60	2.15	1.85	1.72	2.00	−34.90
Digestive diseases (%)								
Gallbladder and biliary diseases	3.68	3.71	3.69	3.85	4.51	4.76	5.24	42.53
Upper digestive system diseases	5.41	5.49	5.74	6.25	6.57	7.24	8.18	51.18
Neurological disorders (%)								
Alzheimer’s disease and other dementias	0.31	0.38	0.46	0.58	0.73	0.94	1.16	278.52
Parkinson’s disease	0.07	0.08	0.11	0.13	0.16	0.19	0.23	246.50
Mental disorders (%)								
Depressive disorders	2.89	3.09	3.07	3.34	3.53	3.84	4.05	40.47
Anxiety disorder	3.57	3.59	3.77	3.64	3.72	3.58	3.67	2.63
Substance use disorders (%)								
Alcohol use disorders	1.28	1.49	1.36	1.32	1.45	1.58	1.70	32.62
Drug use disorders	0.80	0.81	0.75	0.82	0.74	0.76	0.79	−1.38
Diabetes and kidney diseases (%)								
Diabetes mellitus type 2	3.93	4.66	4.94	6.63	7.88	9.62	10.26	161.07
Chronic kidney disease	10.14	10.87	11.58	13.57	15.44	17.96	19.18	89.12
Musculoskeletal disorders (%)								
Osteoarthritis	4.82	5.31	5.84	7.38	8.86	10.45	11.78	144.36

### Statistical analysis

We first described the crude and age-standardised prevalence rates of HBV infection in all of the 31 countries/areas in the WPR from 1990 to 2019. We then analysed the age composition of the HBV-infected population in these countries/areas over the past three decades and compared the proportions of people aged 60 years or older in the HBV-infected population and in the general population.

To estimate the prevalence of NCDs among the HBV-infected population, we first estimated the number of NCD cases in each age group by multiplying the number of age-specific HBV cases (i.e., *n*_*k. i, j*_ in Eq. 1) by the NCD prevalence among the HBV-infected population in the corresponding age group (i.e., *p*_*x, k. i, j*_ in Eq.1). We then calculated the total number of NCD cases among the HBV-infected population by summing the cases in all of the age groups. Finally, we divided the total number of NCD cases among the HBV-infected population by the total HBV-infected population to estimate the total NCD prevalence among the HBV-infected population. The calculation is shown as Eq. 1:

$$P_{x,i,j}=\frac{\sum n_{k,i,j}\times p_{x,k,i,j}}{N_{i,j}}$$ (1)

where *P*_*x, i, j*_ = prevalence of NCD *x* among the HBV-infected population in country *i* in year *j*; *n*_*k, i, j*_ = HBV cases in age group *k* in country *i* in year *j*; *p*_*x, k, i, j*_ = prevalence of NCD *x* among the HBV-infected population in age group *k* in country *i* in year *j*; *N*_*i, j*_ = total HBV-infected population in country *i* in year *j.*

Previous evidence has shown that HBV infection may increase the risk of developing specific non-liver NCDs, such as stomach cancer, DM, and CKD (Cai et al., 2015; Fabrizi et al., 2019; Tian et al., 2020). For NCDs that have been proven to be associated with HBV infection by previous meta-analyses, we multiplied the RR extracted from the meta-analyses by the age-specific prevalence of the NCD among the general population (derived from the GBD database) to estimate the age-specific prevalence of the NCD among the HBV-infected population (i.e., *p*_*x, k. i, j*_ in Eq. (1)). The NCDs adjusted by the RR and details of the corresponding meta-analyses are provided in Supplementary Table S2. For NCDs without pooled evidence showing an association with HBV infection, we assumed that the prevalence of these NCDs were the same in the HBV-infected population and the general population within each age group.

We calculated the NCD prevalence among the HBV-infected population in all 31 of the countries/areas in the WPR from 1990 to 2019. We also calculated the relative changes in prevalence between 1990 and 2019 by subtracting the 1990 prevalence from the 2019 prevalence and dividing it by the 1990 prevalence. To compare the burden of NCDs among the HBV-infected population and the general population, we also analysed the prevalence of the corresponding NCDs among the general population from 1990 to 2019 in the WPR. The differences in NCD prevalence between the HBV-infected population and the general population were mainly attributable to differences in the population age composition and the extra NCD risk induced by HBV infection. Pooled results for the WPR and results for four selected countries (Australia, South Korea, China, and Laos, representing low, lower intermediate, higher intermediate, and high HBV endemicity areas, respectively.) are described in the main results (Schweitzer et al., 2015). The results for the other countries/areas in the WPR are presented in the appendix file. All of the analyses were performed using the R software package, version 4.1.1.

## Results

### HBV prevalence and age composition among the HBV-infected population

**FIGURE 1:**
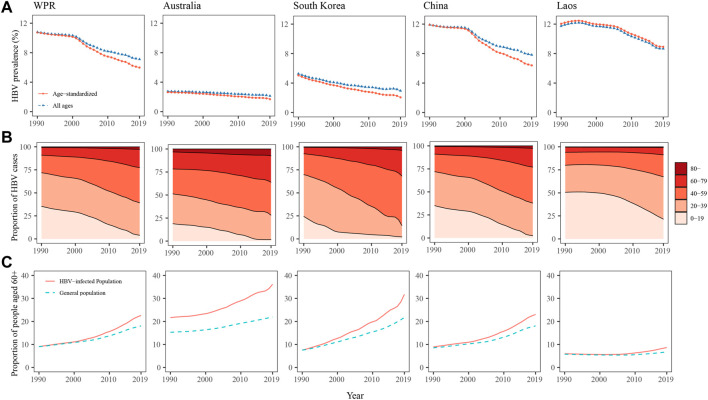
Hepatitis B virus prevalence **(A)**, age composition of the population with hepatitis B virus infection **(B)**, and proportions of people aged 60 years or older among the population with hepatitis B virus infection and the general population **(C)** in the western pacific region, Australia, South Korea, and Laos from 1990 to 2019.

The crude and age-standardised prevalence of HBV infection in the WPR declined from 10.80% to 10.75% in 1990 to 7.11% and 5.98% in 2019, respectively (Figure 1A). The age distribution of the HBV-infected population rapidly shifted to the older age groups. The proportion of individuals aged 0–19 years among the HBV-infected population decreased from 35.42% in 1990 to 3.94% in 2019, while the proportions of those aged 40–59, 60–79, and over 80 years increased from 19.04%, 8.36%, and 0.73% in 1990 to 38.19%, 19.83%, and 2.78% in 2019, respectively (Figure 1B). Over the past three decades, population ageing occurred more rapidly in the HBV-infected population than in the general population in the WPR. The proportion of individuals aged 60 or older in the HBV-infected population increased from 9.09% in 1990 to 22.61% in 2019, whereas this proportion in the general population increased from 9.04% to 18.05% (Figure 1C). The difference in the proportion of people aged 60 or older between the HBV-infected population and the general population has been increasing over the last 15 years (Figure 1C).

As a low-HBV-endemicity area, Australia had crude and age-standardised HBV prevalence of less than 3%, and they showed a decreasing trend from 1990 to 2019 (Figure 1A). The age composition of the HBV-infected population in Australia changed more slowly than in other countries over the past three decades (Figure 1B). HBV-infected individuals in Australia were older than the general population, but population ageing occurred at similar rates (Figure 1C). In South Korea, the crude and age-standardised prevalence of HBV infection declined from 5.26% to 5.03% in 1990 to 2.95% and 2.06% in 2019, respectively (Figure 1A). Over the past three decades, the population structure changed rapidly among those infected with HBV. The proportions of individuals aged 0–19 and 20–39 years among the HBV-infected population in South Korea decreased from 24.33% to 45.45% in 1990 to 2.17% and 12.29% in 2019, respectively, while the proportions of those aged 40–59 and 60–79 increased from 22.63% to 7.04%–53.80% and 27.89%, respectively (Figure 1B). In addition, in South Korea, the HBV-infected population underwent faster population ageing than the general population. The proportion of individuals aged 60 or older among the HBV-infected population increased from 7.59% in 1990 to 31.75% in 2019, while among the general population, this proportion increased from 7.54% to 21.71%. The differences in age composition between the HBV-infected population and the general population in South Korea increased from 1990 to 2019 (Figure 1C). In China, the HBV prevalence and the age composition of the HBV-infected population during the past three decades were similar to those in the pooled WPR results, showing a rapid decline in HBV prevalence and fast population ageing among the HBV-infected population. As a high-HBV-endemicity country, Laos had crude and age-standardised HBV prevalence of more than 8% from 1990 to 2019 (Figure 1A). The HBV-infected population was younger than in other countries. The age composition was similar in the HBV-infected population and the general population before 2005, but after 2005, the HBV-infected population began to age faster (Figure 1B, C).

### NCD prevalence among the HBV-infected population

**FIGURE 2:**
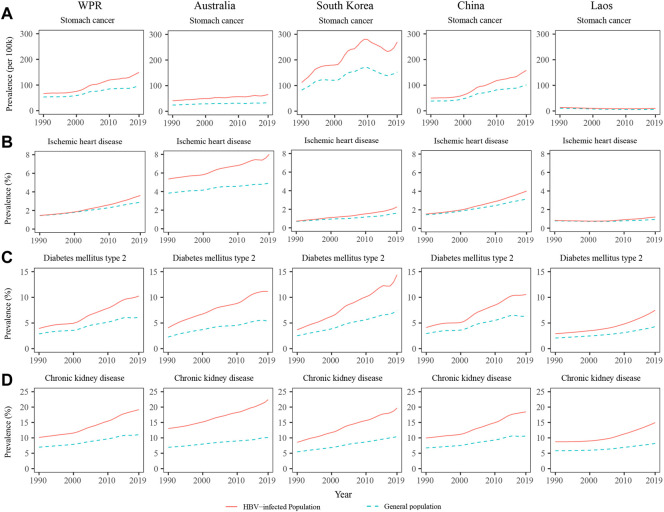
Prevalence of stomach cancer **(A)**, ischemic heart disease **(B)**, diabetes mellitus **(C)**, and chronic kidney disease **(D)** among the population with hepatitis B virus infection and the general population in the western pacific region, Australia, South Korea, and Laos from 1990 to 2019.

The NCD prevalence among the HBV-infected population in the WPR are shown in Table 1. Among the HBV-infected population, the prevalence of 29 of the 31 examined NCDs increased sharply over the past three decades in the WPR (Table 1). In the HBV-infected population, the prevalence of non-Hodgkin’s lymphoma, prostate cancer, and kidney cancer had the largest relative increases from 1990 to 2019, increasing from 11.53, 21.53, and 5.82 per 100,000 persons in 1990 to 102.61, 131.85, and 29.67 in 2019, respectively. However, the prevalence of asthma and drug use disorder showed decreasing trends from 1990 to 2019. The prevalence of NCDs among the general population in the WPR are shown in Supplementary Table S3. In the general population, most NCDs also showed increasing trends, except for asthma, leukaemia, anxiety disorder, and drug use disorder (Supplementary Table S3). The prevalence of most NCDs were higher in the HBV-infected population than in the general population. In addition, from 1990 to 2019, the relative increases in the prevalence of all NCDs except asthma were larger among the HBV-infected population than among the general population. For example, the prevalence of stomach cancer increased from 66.96 per 100,000 persons in 1990 to 149.15 in 2019 (122.74% increase) among the HBV-infected population, while it increased from 53.32 per 100,000 persons in 1990 to 96.26 in 2019 (80.53% increase) among the general population (Tables 1 and Supplementary Table S3). The prevalence of stomach cancer, ischemic heart disease, DM, and CKD among the HBV-infected population and the general population from 1990 to 2019 are presented in Figure 2. The differences in the prevalence of these four NCDs between the HBV-infected population and the general population increased over the past three decades in the WPR (Figure 2).

The prevalence of NCDs among the HBV-infected population and the general population in Australia, South Korea, China, and Laos are presented in Supplementary Tables S4, S5. In Australia, the HBV-infected population had a higher prevalence of NCDs than the general population, and the differences remained relatively stable from 1990 to 2019 (Figure 2). In South Korea and China, the differences in NCDs prevalence between the HBV-infected population and the general population rapidly increased from 1990 to 2019 (Figure 2). In countries with younger populations, such as Laos, the differences in NCD prevalence between the HBV-infected population and the general population were relatively small, but they showed an increasing trend over the past 10 years (Figure 2). The NCD prevalence among the HBV-infected population and the general population from 1990 to 2019 in other countries/areas of the WPR are shown in Supplementary Tables S6, S7.

## Discussion

In the current study, we used the GBD 2019 dataset to examine the age composition of HBV-infected populations and estimated the prevalence of non-liver-related NCDs among this population in the WPR from 1990 to 2019. We found that the HBV-infected population rapidly aged over this time period, and the prevalence of non-liver NCDs among HBV-infected individuals increased in the WPR over the past three decades. In addition, the age composition of the HBV-infected population and their comorbidity burden differed among the various countries/areas in the WPR.

The HBV prevalence showed a pronounced decreasing trend in the WPR from 1990 to 2019, which is consistent with a previous study (Sheena et al., 2022). The notable progress towards regional HBV control in the WPR has mainly been due to the increased coverage of the HepB birth dose and third HepB dose (Wiesen et al., 2016; Woodring et al., 2019; Le et al., 2022). In addition, the WPR has established the goal of eliminating HBV MTCT by 2030, which will be indicated as a 0.1% HBsAg seroprevalence among children under 5 years of age; this goal was established as part of the Regional Framework for the Triple Elimination of Mother-to-Child Transmission of Human Immunodeficiency Virus, Hepatitis B and Syphilis in Asia and the Pacific 2018–2030 (World Health Organization. Regional Office for the Western, 2018). Five countries/areas in the WPR, namely, South Korea, Malaysia, New Zealand, Singapore, and Taiwan, have attained the target of HBV prevalence of ≤ 0.1% among 5-year-olds (Polaris Observatory, 2021). In 2019, the HBV prevalence among children under 5 years of age was less than 1% in more than half of the countries/areas in the WPR (World Health Organization. Regional Office for the Western, 2018; World Health Organization, 2021). In our study, we found that the proportion of people younger than 19 years among the HBV-infected population decreased rapidly over the past three decades, which reflects the marked progress in eliminating HBV MTCT.

Our study found the difference in the proportion of people aged 60 or older between the HBV-infected population and the general population has been increasing over the last 15 years, which indicates the HBV-infected population is ageing at a faster rate than the general population. This phenomenon may be attributed to two factors. Firstly, the number of newly added young individuals in the HBV-infected population is smaller than that in the general population due to the advancements in eliminating HBV MTCT and the increased coverage of HepB among the younger generation in the WPR (Wiesen et al., 2016; Woodring et al., 2019). Additionally, the improvements in the HBV care cascade and the availability of effective anti-HBV drugs have reduced HBV-related deaths and prolonged the life expectancy of HBV-infected individuals (Nguyen et al., 2019; Nguyen et al., 2020). The rapid ageing of the HBV-infected population in the WPR implies a surge in demand for healthcare services among older individuals with HBV infection, who may have complex health conditions (Nguyen et al., 2019; Wong et al., 2020). Considering the large number of HBV-infected individuals in the WPR, this rapid population ageing could impose a substantial healthcare burden (Wiesen et al., 2016). Health policymakers need to consider the healthcare needs of a substantial number of older individuals with HBV infection in the WPR.

The rate of population ageing in the HBV-infected population varies from country to country. For countries with an older population structure (i.e., more than 14% of people are 65 years or older) and stable low HBV prevalence, such as Australia and Japan, the HBV-infected population was older than the general population, and the pace of population ageing was similar to that of the general population. However, countries with rapid population ageing, a significant decline in HBV incidence among the younger generation, and sufficient availability of effective management and treatments to prevent HBV-related deaths, such as South Korea and China, may experience a faster pace of population ageing among the HBV-infected population compared to other countries. These results indicate that the HBV-infected population is growing older, and strategies are needed to address the increasing health and social needs of older HBV-infected individuals, especially in countries with rapid population ageing.

We found that the prevalence of most non-liver NCDs among the HBV-infected population were higher and increased faster than in the general population. The differences in NCD prevalence between the HBV-infected population and the general population were mainly attributable to the different age compositions and the increased risk of developing an NCD, which is induced by HBV infection. In countries where the HBV-infected population is older than the general population, the difference in the prevalence of NCDs between the HBV-infected population and the general population is large as the risk for developing NCDs increases with age. In contrast, countries such as Laos, where the age compositions of the HBV-infected population and the general population are similar, show a relatively small difference in the prevalence of NCDs between the HBV-infected population and the general population. Previous studies have found that HBV infection increases the risk of developing certain non-liver NCDs (Oh et al., 2020; Wong et al., 2020). For example, a study conducted in China detected the expression of anti-HBc antibody and hepatitis B X protein in stomach cancer and pancreatic cancer tissue, and these proteins led to the development of non-liver cancer (Song et al., 2019). The liver damage and persistent inflammatory activities due to HBV infection can lead to glycometabolism disorder, which increases the risk of DM (Tappy and Minehira, 2001; Raddatz and Ramadori, 2007; Cai et al., 2015). Undergoing long-term anti-HBV therapy was found to increase the risk of developing CKD owing to the potential nephrotoxicity of some anti-HBV medications, such as tenofovir disoproxil fumarate (TDF) (European Association for the Study of the Liver, 2017). Our findings indicate that NCD comorbidities in the HBV-infected population will become an increasingly pressing public health issue in the WPR given the large number of HBV-infected cases and population ageing (Kasai, 2021; Sheena et al., 2022).

Our findings indicate that the HBV-infected population in the WPR will face more complex health needs as the burden of non-liver NCDs grows. The comorbidities and the associated medications will greatly impact the choice of oral nucleoside analogues and the long-term prognosis of HBV antiviral treatment. For example, TDF and adefovir dipivoxil are associated with renal impairment and fractures (Wong et al., 2015; European Association for the Study of the Liver, 2017; Wong et al., 2020). Comorbidity with some NCDs adversely impacts the prognosis of individuals infected with HBV. Previous studies conducted in China have shown that coincidental diabetes or metabolic syndrome increases the risk of liver fibrosis and cirrhosis progression in patients with chronic HBV infection (Huang et al., 2013; Wong et al., 2014). Management and treatment of NCDs may be more difficult and complex among people infected with HBV than among those without HBV. Effective community-based strategies for the prevention and control of NCDs have been implemented in the WPR, such as population-based dietary salt reduction, tobacco control actions, and community health education and promotion (Feigin and Krishnamurthi, 2011; Xiao et al., 2014; Jiang et al., 2022). However, HBV-related stigma and discrimination persist in some countries in the WPR, which could hinder people with HBV infection from participating in or accessing community-based NCD management measures (Smith-Palmer et al., 2020). Our results indicate that a two-pronged strategy targeting both HBV control and NCD management should be implemented.

Given the rising burden of non-liver NCDs among the HBV-infected population in the WPR, it is important to implement primary, secondary, and tertiary prevention strategies to address NCD comorbidity in this population. As primary prevention, tailored interventions that aim to prevent age-associated NCDs should be advocated for and implemented among the HBV-infected population, such as enhanced health education and adherence to a healthy diet and lifestyle (Asaria et al., 2007; Arena et al., 2015). This primary prevention is especially important in countries in which the HBV-infected population is rapidly ageing, such as South Korea and China. As secondary prevention, regular screening for common NCDs should be made readily available to middle-aged and older adults infected with HBV, especially in countries in which the HBV-infected population has aged, such as Australia and Japan. Finally, integrated management of HBV and NCDs should be provided to people with HBV–NCD comorbidity to improve their quality of life. Importantly, HBV-related discrimination should be eliminated, which would improve the availability of NCD management for those with HBV–NCD comorbidity.

Our study had several limitations. First, the accuracy of our estimates depended on the quality of data in the GBD database; these data may have been affected by the underreporting of certain diseases, especially in countries without well-functioning disease registry systems. Nevertheless, to the best of our knowledge, the GBD database is the most comprehensive data source that contains age-specific disease burden estimates for both HBV and NCDs. Second, we used the same RRs to adjust the NCD age-specific prevalence rates among the HBV-infected population for all countries/areas in the WPR, which may have resulted in bias in certain countries/areas. Although the pooled RRs used in our study were derived from previous meta-analyses, some countries/areas were not included in those studies, particularly the Pacific island countries. Third, in our study, we did not adjust for risk factors for comorbidities of NCDs among HBV-infected individuals due to the unavailability of prevalence data for NCDs attributable to risk factors in the GBD dataset.

## Conclusion

Our study examined the burden and trends of non-liver NCDs among people with HBV infection in the WPR from 1990 to 2019. Although the WPR has made strong progress towards HBV control over the past three decades, the burden of non-liver NCDs among the HBV-infected population is increasing as this population ages. The synergistic interaction between NCDs and HBV infection often leads to a poor prognosis and low quality of life. Our findings reveal the burden of NCDs and highlight the need for the integrated management of NCDs among the HBV-infected population in the WPR.

## Data Availability

Publicly available datasets were analyzed in this study. This data can be found here: https://ghdx.healthdata.org/gbd-2019.
