# Supplementary material for: Ageing and non-liver comorbidities in population with chronic hepatitis B infection in the western pacific region from 1990 to 2019
Source: Front Physiol. 2023 May 18;14:1176113. doi: 10.3389/fphys.2023.1176113 (PMC10232771; doi:10.3389/fphys.2023.1176113)
Supplement: Supplementary file 1 [file DataSheet1.docx]

**Supplementary file**

**Ageing and non-liver comorbidities in population with chronic hepatitis B infection in the western pacific region from 1990 to 2019**

**Contents**

[Table S1. Countries/areas of the western pacific region defined by World Health Organization and countries/areas included in Global Burden of Disease databases. 2](#_Toc128251128)

[Table S2. The noncommunicable diseases adjusted by relative risk values and details of the corresponding meta-analyses. 3](#_Toc128251129)

[Table S3. Prevalence of noncommunicable diseases among the general population in the western pacific region, 1990–2019. 4](#_Toc128251130)

[Table S4. Prevalence of noncommunicable diseases among the population with hepatitis B virus infection in Australia, South Korea, China and Laos, 1990-2019. 5](#_Toc128251131)

[Table S5. Prevalence of noncommunicable diseases among the general population in Australia, South Korea, China and Laos, 1990-2019. 10](#_Toc128251132)

[Table S6. Prevalence of noncommunicable diseases among population with hepatitis B virus infection in other countries/areas of the western pacific region, 1990-2019. 15](#_Toc128251133)

[Table S7. Prevalence of noncommunicable diseases among the general population in other countries/areas of the western pacific region, 1990-2019. 43](#_Toc128251134)

# ****Table S1. Countries/areas of the western pacific region defined by World Health Organization and countries/areas included in Global Burden of Disease databases.****

| Countries/areas of WPR defined by WHO | Whether included in GBD 2019 |
| --- | --- |
| American Samoa (USA) | Yes |
| New Caledonia (France) | No |
| Australia | Yes |
| New Zealand | Yes |
| Brunei Darussalam | Yes |
| Niue | Yes |
| Cambodia | Yes |
| Northern Mariana Islands, Commonwealth of the (USA) | Yes |
| China | Yes |
| Palau | Yes |
| Cook Islands | Yes |
| Papua New Guinea | Yes |
| Fiji | Yes |
| Philippines | Yes |
| French Polynesia (France) | No |
| Pitcairn Island (UK) | No |
| Guam (USA) | Yes |
| South Korea | Yes |
| Hong Kong SAR (China) | No |
| Samoa | Yes |
| Japan | Yes |
| Singapore | Yes |
| Kiribati | Yes |
| Solomon Islands | Yes |
| Lao People's Democratic Republic | Yes |
| Tokelau (New Zealand) | Yes |
| Macao SAR (China) | No |
| Tonga | Yes |
| Malaysia | Yes |
| Tuvalu | Yes |
| Marshall Islands | Yes |
| Vanuatu | Yes |
| Micronesia, Federated States of | Yes |
| Viet Nam | Yes |
| Mongolia | Yes |
| Wallis and Futuna (France) | No |
| Nauru | Yes |

GBD, global burden of disease; WHO, world health organization; WPR, western pacific region.

# Table S2. The noncommunicable diseases adjusted by relative risk values and details of the corresponding meta-analyses.

| NCDs | Pooled RR (95% CI) | Studies included | Countries/areas involved | Reference |
| --- | --- | --- | --- | --- |
| Stomach cancer | 1.23 (1.14-1.33) | 8 | China, Taiwan, Australia, U.S, South Korea | Tian et al [1] |
| Pancreatic cancer | 1.26(1.09-1.46) | 15 | China, Taiwan, Danmark, Sweden, Australia, U.S, South Korea, | Tian et al [1] |
| Non-Hodgkin's lymphoma | 2.50(2.20-2.83) | 58 | China, Taiwan, Danmark, Sweden, Australia, U.S, South Korea, France, Japan, Romania, Egypt, Italy, Saudi Arabia, Turkey, Singapore, Israel, Europe | Li et al [2] |
| Esophageal cancer | 1.19(1.01-1.36) | 10 | China, South Korea, U.S | Geng et al [3] |
| Leukemia | 1.54(1.26-1.88) | 5 | China, Taiwan, South Korea | Tian et al [1] |
| Multiple myeloma | 1.41(1.03-1.94) | 10 | China, South Korea, Australia, U.S, Turkey, Europe, Nigera, Sweden | Dalia et al[4] |
| Diabetes mellitus type 2 | 1.33 (1.09-1.62) | 15 | China, Taiwan, Japan, U.S, Iran, Pakistan, Israel | Cai et al [5] |
| Chronic kidney disease | 1.41(1.09-1.92) | 6 | China, Taiwan, South Korea, U.S | Fabrizi et al [6] |

NCD, noncommunicable disease; RR, relative risk.

# Table S3. Prevalence of noncommunicable diseases among the general population in the western pacific region, 1990–2019.

| Noncommunicable diseases | Prevalence | | | | | | | Relative change,1990-2019 (%) |
| --- | --- | --- | --- | --- | --- | --- | --- | --- |
|  | 1990 | 1995 | 2000 | 2005 | 2010 | 2015 | 2019 |  |
| **Neoplasms/Cancer (per 100,000 persons)** |  |  |  |  |  |  |  |  |
| Lip and oral cavity cancer | 5.04 | 5.84 | 6.93 | 8.85 | 11.76 | 13.65 | 14.98 | 197.22 |
| Colon and rectum cancer | 64.68 | 77.78 | 95.98 | 135.52 | 184.68 | 219.09 | 262.41 | 305.71 |
| Stomach cancer | 53.32 | 54.28 | 59.26 | 75.76 | 84.82 | 86.82 | 96.26 | 80.53 |
| Pancreatic cancer | 2.68 | 3.03 | 3.65 | 4.75 | 5.99 | 6.89 | 7.96 | 197.01 |
| Non-Hodgkin's lymphoma | 4.57 | 6.58 | 9.00 | 12.86 | 22.14 | 28.82 | 33.97 | 643.33 |
| Gallbladder and biliary tract cancer | 2.51 | 2.67 | 2.99 | 3.89 | 4.42 | 4.63 | 5.01 | 99.60 |
| Kidney cancer | 5.94 | 6.86 | 9.15 | 14.15 | 19.13 | 21.45 | 24.63 | 314.65 |
| Ovarian cancer | 6.57 | 8.01 | 9.54 | 11.13 | 13.06 | 14.58 | 16.37 | 149.16 |
| Cervical cancer | 19.41 | 20.91 | 25.06 | 32.01 | 37.37 | 41.04 | 42.00 | 116.38 |
| Breast cancer | 93.63 | 105.48 | 130.29 | 163.97 | 210.56 | 245.46 | 281.96 | 201.14 |
| Oesophageal cancer | 18.67 | 20.34 | 24.34 | 30.40 | 29.36 | 27.70 | 30.93 | 65.67 |
| Tracheal, bronchus and lung cancer | 26.54 | 30.32 | 37.17 | 48.09 | 60.84 | 69.84 | 80.45 | 203.13 |
| Prostate cancer | 21.64 | 28.35 | 38.33 | 56.14 | 74.78 | 89.49 | 105.72 | 388.54 |
| Thyroid cancer | 9.78 | 12.08 | 15.29 | 20.13 | 26.72 | 27.18 | 29.55 | 202.15 |
| Leukaemia | 67.27 | 64.08 | 59.11 | 51.62 | 53.58 | 57.56 | 64.80 | -3.67 |
| Multiple myeloma | 1.68 | 1.97 | 2.42 | 3.06 | 3.87 | 4.51 | 5.15 | 206.55 |
| **Cardiovascular diseases (%)** |  |  |  |  |  |  |  |  |
| Stroke | 1.06 | 1.13 | 1.26 | 1.34 | 1.51 | 1.74 | 1.97 | 84.97 |
| Ischemic heart disease | 1.45 | 1.59 | 1.80 | 2.05 | 2.30 | 2.64 | 2.90 | 100.20 |
| **Chronic respiratory diseases (%)** |  |  |  |  |  |  |  |  |
| Chronic obstructive pulmonary disease | 2.20 | 2.40 | 2.57 | 2.70 | 2.74 | 2.96 | 3.06 | 39.24 |
| Asthma | 3.11 | 3.04 | 2.65 | 2.21 | 2.03 | 2.03 | 2.32 | -25.36 |
| **Digestive diseases (%)** |  |  |  |  |  |  |  |  |
| Gallbladder and biliary diseases | 3.56 | 3.56 | 3.53 | 3.53 | 3.95 | 4.02 | 4.21 | 18.18 |
| Upper digestive system diseases | 5.21 | 5.25 | 5.48 | 5.74 | 5.79 | 6.14 | 6.65 | 27.60 |
| **Neurological disorders (%)** |  |  |  |  |  |  |  |  |
| Alzheimer's disease and other dementias | 0.32 | 0.40 | 0.47 | 0.56 | 0.68 | 0.86 | 1.00 | 209.00 |
| Parkinson's disease | 0.07 | 0.08 | 0.10 | 0.12 | 0.14 | 0.16 | 0.18 | 178.52 |
| **Mental disorders (%)** |  |  |  |  |  |  |  |  |
| Depressive disorders | 2.77 | 2.95 | 2.92 | 3.07 | 3.10 | 3.27 | 3.33 | 20.26 |
| Anxiety disorder | 3.46 | 3.48 | 3.66 | 3.45 | 3.45 | 3.33 | 3.41 | -1.64 |
| **Substance use disorders (%)** |  |  |  |  |  |  |  |  |
| Alcohol use disorders | 1.22 | 1.41 | 1.28 | 1.21 | 1.28 | 1.34 | 1.39 | 13.22 |
| Drug use disorders | 0.76 | 0.77 | 0.72 | 0.76 | 0.67 | 0.66 | 0.69 | -9.93 |
| **Diabetes and kidney diseases (%)** |  |  |  |  |  |  |  |  |
| Diabetes mellitus type 2 | 2.87 | 3.37 | 3.57 | 4.55 | 5.16 | 6.06 | 6.11 | 112.56 |
| Chronic kidney disease | 6.99 | 7.42 | 7.90 | 8.86 | 9.66 | 10.84 | 11.04 | 57.96 |
| **Musculoskeletal disorders (%)** |  |  |  |  |  |  |  |  |
| Osteoarthritis | 4.72 | 5.13 | 5.64 | 6.75 | 7.70 | 8.74 | 9.30 | 97.18 |

# Table S4. Prevalence of noncommunicable diseases among the population with hepatitis B virus infection in Australia, South Korea, China and Laos, 1990-2019.

| Noncommunicable diseases | Prevalence | | | | | | | Relative change,1990-2019 (%) |
| --- | --- | --- | --- | --- | --- | --- | --- | --- |
|  | 1990 | 1995 | 2000 | 2005 | 2010 | 2015 | 2019 |  |
| **Australia** |  |  |  |  |  |  |  |  |
| **Neoplasms/Cancer (per 100,000 persons)** |  |  |  |  |  |  |  |  |
| Lip and oral cavity cancer | 34.08 | 35.59 | 35.29 | 33.24 | 36.18 | 33.62 | 31.89 | -6.43 |
| Colon and rectum cancer | 436.00 | 504.20 | 584.17 | 636.57 | 678.30 | 716.38 | 788.27 | 80.80 |
| Stomach cancer | 41.36 | 45.26 | 49.57 | 53.07 | 56.94 | 61.92 | 65.30 | 57.88 |
| Pancreatic cancer | 13.72 | 15.36 | 17.56 | 20.45 | 23.77 | 26.64 | 29.45 | 114.65 |
| Non-Hodgkin's lymphoma | 246.27 | 321.80 | 371.87 | 352.61 | 368.97 | 393.59 | 433.34 | 75.96 |
| Gallbladder and biliary tract cancer | 5.35 | 5.61 | 5.95 | 6.16 | 6.53 | 6.63 | 6.97 | 30.28 |
| Kidney cancer | 47.21 | 61.44 | 75.73 | 86.91 | 96.18 | 101.89 | 108.93 | 130.74 |
| Ovarian cancer | 32.15 | 33.38 | 34.18 | 35.48 | 35.24 | 34.90 | 37.25 | 15.86 |
| Cervical cancer | 52.31 | 48.05 | 43.62 | 43.25 | 49.66 | 56.27 | 56.44 | 7.90 |
| Breast cancer | 701.25 | 778.55 | 882.36 | 936.32 | 1005.91 | 1071.72 | 1126.66 | 60.66 |
| Oesophageal cancer | 14.72 | 16.75 | 18.71 | 20.61 | 21.88 | 23.29 | 25.16 | 70.92 |
| Tracheal, bronchus and lung cancer | 87.80 | 95.14 | 104.60 | 123.30 | 140.07 | 148.33 | 157.19 | 79.03 |
| Prostate cancer | 440.46 | 667.29 | 750.41 | 989.92 | 1081.40 | 1116.38 | 1247.98 | 183.34 |
| Thyroid cancer | 27.07 | 32.43 | 44.69 | 57.19 | 71.63 | 77.14 | 77.40 | 185.93 |
| Leukaemia | 74.35 | 91.76 | 114.26 | 121.55 | 125.29 | 151.62 | 167.63 | 125.46 |
| Multiple myeloma | 29.45 | 38.11 | 49.30 | 57.64 | 62.92 | 69.81 | 76.79 | 160.75 |
| **Cardiovascular diseases (%)** |  |  |  |  |  |  |  |  |
| Stroke | 1.42 | 1.31 | 1.28 | 1.31 | 1.34 | 1.44 | 1.53 | 7.87 |
| Ischemic heart disease | 5.35 | 5.62 | 5.81 | 6.47 | 6.80 | 7.44 | 7.99 | 49.46 |
| **Chronic respiratory diseases (%)** |  |  |  |  |  |  |  |  |
| Chronic obstructive pulmonary disease | 5.52 | 6.03 | 6.53 | 7.10 | 7.38 | 7.80 | 8.45 | 53.27 |
| Asthma | 10.94 | 11.30 | 11.54 | 10.73 | 7.45 | 7.20 | 7.45 | -31.86 |
| **Digestive diseases (%)** |  |  |  |  |  |  |  |  |
| Gallbladder and biliary diseases | 2.42 | 2.46 | 2.52 | 2.64 | 2.70 | 2.84 | 3.06 | 26.44 |
| Upper digestive system diseases | 11.26 | 11.48 | 10.93 | 11.52 | 12.09 | 13.80 | 14.27 | 26.73 |
| **Neurological disorders (%)** |  |  |  |  |  |  |  |  |
| Alzheimer's disease and other dementias | 1.09 | 1.21 | 1.39 | 1.59 | 1.77 | 2.01 | 2.24 | 105.88 |
| Parkinson's disease | 0.22 | 0.27 | 0.31 | 0.38 | 0.35 | 0.37 | 0.41 | 86.10 |
| **Mental disorders (%)** |  |  |  |  |  |  |  |  |
| Depressive disorders | 5.20 | 5.38 | 5.52 | 6.06 | 6.02 | 5.80 | 5.37 | 3.25 |
| Anxiety disorder | 6.42 | 5.99 | 5.82 | 6.35 | 6.33 | 6.16 | 6.38 | -0.68 |
| **Substance use disorders (%)** |  |  |  |  |  |  |  |  |
| Alcohol use disorders | 1.96 | 2.10 | 2.20 | 2.09 | 2.23 | 2.43 | 2.36 | 20.26 |
| Drug use disorders | 2.35 | 2.23 | 2.09 | 2.06 | 2.17 | 2.01 | 1.79 | -23.83 |
| **Diabetes and kidney diseases (%)** |  |  |  |  |  |  |  |  |
| Diabetes mellitus type 2 | 4.06 | 5.62 | 6.75 | 8.11 | 8.80 | 10.73 | 11.15 | 174.67 |
| Chronic kidney disease | 13.03 | 13.87 | 15.17 | 16.87 | 18.26 | 20.06 | 22.42 | 72.11 |
| **Musculoskeletal disorders (%)** |  |  |  |  |  |  |  |  |
| Osteoarthritis | 11.81 | 12.64 | 13.99 | 15.63 | 16.65 | 18.33 | 20.49 | 73.55 |
| **South Korea** |  |  |  |  |  |  |  |  |
| **Neoplasms/Cancer (per 100,000 persons)** |  |  |  |  |  |  |  |  |
| Lip and oral cavity cancer | 3.24 | 5.39 | 7.87 | 9.66 | 11.63 | 11.90 | 13.58 | 319.14 |
| Colon and rectum cancer | 59.73 | 117.00 | 161.49 | 266.32 | 394.71 | 446.52 | 546.12 | 814.31 |
| Stomach cancer | 111.79 | 169.19 | 179.91 | 241.17 | 279.31 | 240.39 | 269.76 | 141.31 |
| Pancreatic cancer | 5.60 | 8.49 | 9.06 | 11.29 | 16.15 | 20.81 | 26.41 | 371.61 |
| Non-Hodgkin's lymphoma | 10.63 | 41.84 | 58.28 | 86.46 | 125.99 | 162.35 | 196.45 | 1748.07 |
| Gallbladder and biliary tract cancer | 8.16 | 10.40 | 12.05 | 14.92 | 17.60 | 20.16 | 25.08 | 207.35 |
| Kidney cancer | 4.28 | 9.46 | 16.14 | 26.85 | 39.48 | 41.22 | 46.86 | 994.86 |
| Ovarian cancer | 6.28 | 10.38 | 13.78 | 17.75 | 22.19 | 26.28 | 29.18 | 364.65 |
| Cervical cancer | 51.19 | 63.06 | 65.56 | 61.28 | 62.82 | 57.44 | 56.84 | 11.04 |
| Breast cancer | 111.92 | 142.37 | 199.02 | 306.38 | 430.36 | 523.07 | 600.43 | 436.48 |
| Oesophageal cancer | 9.07 | 13.95 | 13.94 | 17.63 | 22.93 | 27.92 | 35.49 | 291.29 |
| Tracheal, bronchus and lung cancer | 22.85 | 39.64 | 57.14 | 81.79 | 118.99 | 149.34 | 186.33 | 715.45 |
| Prostate cancer | 14.55 | 25.65 | 42.79 | 84.23 | 141.54 | 186.18 | 233.99 | 1508.18 |
| Thyroid cancer | 14.01 | 28.70 | 65.77 | 152.36 | 224.14 | 129.06 | 111.78 | 697.86 |
| Leukaemia | 42.04 | 43.82 | 41.12 | 51.98 | 71.46 | 108.43 | 121.30 | 188.53 |
| Multiple myeloma | 2.82 | 3.54 | 5.23 | 8.66 | 13.01 | 16.51 | 20.39 | 623.05 |
| **Cardiovascular diseases (%)** |  |  |  |  |  |  |  |  |
| Stroke | 1.75 | 1.97 | 2.11 | 2.21 | 2.31 | 2.54 | 2.74 | 56.74 |
| Ischemic heart disease | 0.73 | 0.91 | 1.11 | 1.26 | 1.51 | 1.77 | 2.25 | 210.53 |
| **Chronic respiratory diseases (%)** |  |  |  |  |  |  |  |  |
| Chronic obstructive pulmonary disease | 1.29 | 1.75 | 2.23 | 2.90 | 3.40 | 3.94 | 4.20 | 224.50 |
| Asthma | 3.56 | 3.86 | 3.59 | 3.72 | 3.61 | 3.45 | 3.57 | 0.26 |
| **Digestive diseases (%)** |  |  |  |  |  |  |  |  |
| Gallbladder and biliary diseases | 4.50 | 4.90 | 4.99 | 5.24 | 5.55 | 6.03 | 6.56 | 45.64 |
| Upper digestive system diseases | 7.55 | 8.11 | 8.79 | 8.54 | 10.70 | 12.55 | 11.70 | 55.00 |
| **Neurological disorders (%)** |  |  |  |  |  |  |  |  |
| Alzheimer's disease and other dementias | 0.28 | 0.37 | 0.49 | 0.67 | 0.91 | 1.21 | 1.46 | 419.20 |
| Parkinson's disease | 0.04 | 0.05 | 0.06 | 0.09 | 0.12 | 0.16 | 0.19 | 442.17 |
| **Mental disorders (%)** |  |  |  |  |  |  |  |  |
| Depressive disorders | 2.36 | 2.45 | 2.55 | 2.63 | 2.80 | 2.92 | 2.86 | 21.32 |
| Anxiety disorder | 3.80 | 3.83 | 3.93 | 3.54 | 3.73 | 4.09 | 3.64 | -3.98 |
| **Substance use disorders (%)** |  |  |  |  |  |  |  |  |
| Alcohol use disorders | 3.86 | 4.02 | 3.91 | 4.91 | 4.59 | 4.53 | 3.48 | -10.01 |
| Drug use disorders | 1.09 | 1.12 | 1.04 | 0.86 | 0.71 | 0.60 | 0.55 | -49.68 |
| **Diabetes and kidney diseases (%)** |  |  |  |  |  |  |  |  |
| Diabetes mellitus type 2 | 3.69 | 4.96 | 6.30 | 8.61 | 10.11 | 12.29 | 14.38 | 290.33 |
| Chronic kidney disease | 8.57 | 10.15 | 11.73 | 14.11 | 15.64 | 17.78 | 19.66 | 129.50 |
| **Musculoskeletal disorders (%)** |  |  |  |  |  |  |  |  |
| Osteoarthritis | 7.27 | 8.92 | 11.00 | 13.96 | 17.82 | 22.15 | 23.71 | 225.89 |
| **China** |  |  |  |  |  |  |  |  |
| **Neoplasms/Cancer (per 100,000 persons)** |  |  |  |  |  |  |  |  |
| Lip and oral cavity cancer | 3.70 | 4.14 | 4.84 | 7.03 | 10.83 | 13.47 | 15.72 | 324.86 |
| Colon and rectum cancer | 39.56 | 45.21 | 62.23 | 111.39 | 178.53 | 228.34 | 304.46 | 669.62 |
| Stomach cancer | 49.56 | 51.17 | 62.23 | 95.47 | 118.17 | 130.48 | 157.60 | 218.00 |
| Pancreatic cancer | 2.40 | 2.66 | 3.43 | 4.92 | 6.60 | 7.88 | 10.14 | 322.50 |
| Non-Hodgkin's lymphoma | 0.80 | 1.85 | 4.82 | 14.32 | 44.23 | 66.87 | 87.35 | 10818.75 |
| Gallbladder and biliary tract cancer | 1.22 | 1.25 | 1.54 | 2.63 | 3.30 | 3.61 | 4.21 | 245.08 |
| Kidney cancer | 4.13 | 4.35 | 6.27 | 11.76 | 18.15 | 21.55 | 27.13 | 556.90 |
| Ovarian cancer | 4.81 | 5.96 | 7.42 | 9.24 | 11.72 | 13.90 | 17.00 | 253.43 |
| Cervical cancer | 13.96 | 15.73 | 21.33 | 31.74 | 39.90 | 46.70 | 50.25 | 259.96 |
| Breast cancer | 68.92 | 78.68 | 101.61 | 139.68 | 197.84 | 246.13 | 312.48 | 353.40 |
| Oesophageal cancer | 27.03 | 29.32 | 35.03 | 46.65 | 46.17 | 44.27 | 53.00 | 96.08 |
| Tracheal, bronchus and lung cancer | 24.96 | 28.19 | 35.16 | 48.63 | 65.85 | 80.34 | 101.57 | 306.93 |
| Prostate cancer | 12.22 | 15.85 | 22.60 | 36.00 | 56.44 | 76.16 | 101.63 | 731.67 |
| Thyroid cancer | 6.16 | 7.86 | 10.17 | 12.94 | 18.99 | 22.33 | 26.78 | 334.74 |
| Leukaemia | 103.50 | 98.80 | 90.26 | 67.13 | 72.99 | 84.70 | 105.34 | 1.78 |
| Multiple myeloma | 1.55 | 1.81 | 2.23 | 2.98 | 4.22 | 5.49 | 6.97 | 349.68 |
| **Cardiovascular diseases (%)** |  |  |  |  |  |  |  |  |
| Stroke | 1.03 | 1.12 | 1.25 | 1.43 | 1.72 | 2.10 | 2.54 | 146.89 |
| Ischemic heart disease | 1.55 | 1.74 | 1.98 | 2.44 | 2.90 | 3.48 | 4.02 | 159.57 |
| **Chronic respiratory diseases (%)** |  |  |  |  |  |  |  |  |
| Chronic obstructive pulmonary disease | 2.45 | 2.71 | 2.89 | 3.20 | 3.35 | 3.71 | 3.98 | 62.30 |
| Asthma | 2.13 | 2.09 | 1.80 | 1.42 | 1.22 | 1.13 | 1.47 | -31.23 |
| **Digestive diseases (%)** |  |  |  |  |  |  |  |  |
| Gallbladder and biliary diseases | 4.05 | 4.08 | 4.03 | 4.21 | 5.01 | 5.25 | 5.77 | 42.35 |
| Upper digestive system diseases | 5.21 | 5.24 | 5.49 | 5.99 | 6.13 | 6.76 | 7.85 | 50.58 |
| **Neurological disorders (%)** |  |  |  |  |  |  |  |  |
| Alzheimer's disease and other dementias | 0.29 | 0.38 | 0.45 | 0.57 | 0.72 | 0.94 | 1.16 | 301.38 |
| Parkinson's disease | 0.07 | 0.09 | 0.12 | 0.15 | 0.18 | 0.21 | 0.25 | 264.99 |
| **Mental disorders (%)** |  |  |  |  |  |  |  |  |
| Depressive disorders | 3.02 | 3.27 | 3.19 | 3.48 | 3.67 | 4.04 | 4.28 | 41.74 |
| Anxiety disorder | 3.62 | 3.63 | 3.83 | 3.64 | 3.71 | 3.48 | 3.57 | -1.44 |
| **Substance use disorders (%)** |  |  |  |  |  |  |  |  |
| Alcohol use disorders | 1.33 | 1.59 | 1.41 | 1.33 | 1.46 | 1.60 | 1.77 | 33.13 |
| Drug use disorders | 0.79 | 0.79 | 0.73 | 0.81 | 0.70 | 0.73 | 0.79 | 0.70 |
| **Diabetes and kidney diseases (%)** |  |  |  |  |  |  |  |  |
| Diabetes mellitus type 2 | 4.11 | 4.92 | 5.09 | 7.13 | 8.49 | 10.33 | 10.57 | 157.03 |
| Chronic kidney disease | 9.96 | 10.58 | 11.14 | 13.14 | 14.94 | 17.66 | 18.45 | 85.27 |
| **Musculoskeletal disorders (%)** |  |  |  |  |  |  |  |  |
| Osteoarthritis | 4.64 | 5.11 | 5.59 | 7.27 | 8.68 | 10.36 | 11.82 | 154.97 |
| **Laos** |  |  |  |  |  |  |  |  |
| **Neoplasms/Cancer (per 100,000 persons)** |  |  |  |  |  |  |  |  |
| Lip and oral cavity cancer | 5.95 | 5.72 | 5.31 | 5.33 | 6.27 | 7.66 | 9.33 | 56.81 |
| Colon and rectum cancer | 16.84 | 17.44 | 18.29 | 20.29 | 25.12 | 32.49 | 41.58 | 146.91 |
| Stomach cancer | 13.89 | 12.07 | 10.18 | 8.94 | 8.76 | 9.02 | 9.92 | -28.58 |
| Pancreatic cancer | 1.19 | 1.19 | 1.20 | 1.33 | 1.61 | 2.03 | 2.54 | 113.45 |
| Non-Hodgkin's lymphoma | N.A | N.A | N.A | N.A | N.A | N.A | N.A | N.A |
| Gallbladder and biliary tract cancer | 0.96 | 0.87 | 0.76 | 0.71 | 0.76 | 0.87 | 1.01 | 5.21 |
| Kidney cancer | 3.18 | 3.20 | 3.28 | 3.41 | 4.03 | 5.02 | 6.31 | 98.43 |
| Ovarian cancer | 5.49 | 6.16 | 7.06 | 9.09 | 12.11 | 15.66 | 18.73 | 241.17 |
| Cervical cancer | 33.25 | 32.12 | 30.06 | 29.43 | 31.75 | 35.73 | 40.75 | 22.56 |
| Breast cancer | 55.73 | 56.68 | 58.34 | 64.01 | 78.71 | 97.76 | 119.20 | 113.89 |
| Oesophageal cancer | 3.61 | 3.23 | 2.81 | 2.56 | 2.66 | 2.93 | 3.35 | -7.20 |
| Tracheal, bronchus and lung cancer | 13.83 | 13.15 | 12.40 | 11.97 | 13.23 | 15.32 | 18.26 | 32.03 |
| Prostate cancer | 7.18 | 7.61 | 7.98 | 8.90 | 11.60 | 15.58 | 20.80 | 189.69 |
| Thyroid cancer | 6.82 | 7.49 | 7.92 | 8.89 | 11.18 | 14.11 | 17.50 | 156.60 |
| Leukaemia | 113.16 | 99.25 | 84.85 | 68.35 | 54.11 | 44.74 | 41.32 | -63.49 |
| Multiple myeloma | 1.24 | 1.19 | 1.11 | 1.11 | 1.29 | 1.57 | 1.91 | 54.03 |
| **Cardiovascular diseases (%)** |  |  |  |  |  |  |  |  |
| Stroke | 1.16 | 1.10 | 1.08 | 1.07 | 1.16 | 1.29 | 1.44 | 24.02 |
| Ischemic heart disease | 0.84 | 0.79 | 0.76 | 0.77 | 0.91 | 1.04 | 1.20 | 43.13 |
| **Chronic respiratory diseases (%)** |  |  |  |  |  |  |  | 73.10 |
| Chronic obstructive pulmonary disease | 1.35 | 1.35 | 1.37 | 1.46 | 1.69 | 2.01 | 2.33 | 73.10 |
| Asthma | 2.47 | 2.43 | 2.22 | 2.07 | 1.96 | 1.85 | 1.90 | -22.85 |
| **Digestive diseases (%)** |  |  |  |  |  |  |  |  |
| Gallbladder and biliary diseases | 1.37 | 1.33 | 1.29 | 1.30 | 1.38 | 1.50 | 1.66 | 21.45 |
| Upper digestive system diseases | 4.45 | 4.41 | 4.44 | 4.68 | 5.25 | 5.95 | 6.54 | 46.91 |
| **Neurological disorders (%)** |  |  |  |  |  |  |  |  |
| Alzheimer's disease and other dementias | 0.18 | 0.18 | 0.19 | 0.19 | 0.22 | 0.26 | 0.30 | 65.50 |
| Parkinson's disease | 0.04 | 0.03 | 0.04 | 0.04 | 0.04 | 0.05 | 0.06 | 65.57 |
| **Mental disorders (%)** |  |  |  |  |  |  |  |  |
| Depressive disorders | 2.40 | 2.40 | 2.31 | 2.47 | 2.67 | 3.07 | 3.27 | 35.81 |
| Anxiety disorder | 3.80 | 3.83 | 3.89 | 4.13 | 4.62 | 5.02 | 5.23 | 37.90 |
| **Substance use disorders (%)** |  |  |  |  |  |  |  |  |
| Alcohol use disorders | 0.74 | 0.76 | 0.78 | 0.81 | 0.89 | 1.00 | 1.09 | 47.03 |
| Drug use disorders | 0.46 | 0.47 | 0.50 | 0.54 | 0.60 | 0.64 | 0.66 | 42.35 |
| **Diabetes and kidney diseases (%)** |  |  |  |  |  |  |  |  |
| Diabetes mellitus type 2 | 2.91 | 3.17 | 3.49 | 3.95 | 4.81 | 6.09 | 7.48 | 157.20 |
| Chronic kidney disease | 8.75 | 8.77 | 9.00 | 9.69 | 11.26 | 13.13 | 14.96 | 70.99 |
| **Musculoskeletal disorders (%)** |  |  |  |  |  |  |  |  |
| Osteoarthritis | 2.00 | 1.94 | 1.96 | 2.11 | 2.46 | 2.93 | 3.42 | 71.02 |

# Table S5. Prevalence of noncommunicable diseases among the general population in Australia, South Korea, China and Laos, 1990-2019.

| Noncommunicable diseases | Prevalence | | | | | | | Relative change,1990-2019 (%) |
| --- | --- | --- | --- | --- | --- | --- | --- | --- |
|  | 1990 | 1995 | 2000 | 2005 | 2010 | 2015 | 2019 |  |
| **Australia** |  |  |  |  |  |  |  |  |
| **Neoplasms/Cancer (per 100,000 persons)** |  |  |  |  |  |  |  |  |
| Lip and oral cavity cancer | 26.18 | 27.50 | 27.17 | 24.88 | 26.10 | 23.19 | 21.06 | -19.56 |
| Colon and rectum cancer | 316.31 | 366.24 | 421.57 | 445.25 | 459.12 | 464.24 | 488.80 | 54.53 |
| Stomach cancer | 24.45 | 26.78 | 29.18 | 30.28 | 31.48 | 32.75 | 33.00 | 34.97 |
| Pancreatic cancer | 7.80 | 8.72 | 9.94 | 11.22 | 12.59 | 13.46 | 14.23 | 82.44 |
| Non-Hodgkin's lymphoma | 73.75 | 95.77 | 109.02 | 99.13 | 100.55 | 101.04 | 106.70 | 44.68 |
| Gallbladder and biliary tract cancer | 3.80 | 3.98 | 4.21 | 4.23 | 4.34 | 4.20 | 4.23 | 11.32 |
| Kidney cancer | 36.26 | 47.18 | 57.87 | 64.72 | 69.34 | 70.20 | 71.72 | 97.79 |
| Ovarian cancer | 25.13 | 26.18 | 26.57 | 26.81 | 25.76 | 24.48 | 25.17 | 0.16 |
| Cervical cancer | 42.98 | 39.52 | 35.58 | 34.07 | 37.76 | 41.22 | 39.71 | -7.61 |
| Breast cancer | 521.71 | 584.79 | 660.05 | 680.28 | 703.86 | 715.39 | 719.13 | 37.84 |
| Oesophageal cancer | 8.93 | 10.17 | 11.32 | 12.11 | 12.45 | 12.65 | 13.07 | 46.36 |
| Tracheal, bronchus and lung cancer | 63.99 | 69.27 | 75.62 | 86.40 | 94.77 | 95.98 | 97.42 | 52.24 |
| Prostate cancer | 308.42 | 470.71 | 530.18 | 682.18 | 721.21 | 710.19 | 760.26 | 146.50 |
| Thyroid cancer | 22.01 | 26.47 | 36.20 | 44.83 | 54.15 | 55.91 | 54.10 | 145.80 |
| Leukaemia | 38.67 | 47.38 | 58.40 | 60.67 | 60.78 | 69.60 | 73.07 | 88.96 |
| Multiple myeloma | 15.18 | 19.72 | 25.40 | 28.82 | 30.33 | 32.07 | 33.74 | 122.27 |
| **Cardiovascular diseases (%)** |  |  |  |  |  |  |  |  |
| Stroke | 1.05 | 0.97 | 0.95 | 0.94 | 0.94 | 0.97 | 0.99 | -5.87 |
| Ischemic heart disease | 3.83 | 4.04 | 4.16 | 4.50 | 4.57 | 4.77 | 4.91 | 28.19 |
| **Chronic respiratory diseases (%)** |  |  |  |  |  |  |  |  |
| Chronic obstructive pulmonary disease | 4.01 | 4.39 | 4.72 | 4.96 | 4.99 | 5.03 | 5.23 | 30.28 |
| Asthma | 11.43 | 11.89 | 12.07 | 11.04 | 8.14 | 7.96 | 8.33 | -27.07 |
| **Digestive diseases (%)** |  |  |  |  |  |  |  |  |
| Gallbladder and biliary diseases | 1.96 | 2.00 | 2.03 | 2.07 | 2.05 | 2.08 | 2.16 | 10.14 |
| Upper digestive system diseases | 9.42 | 9.63 | 9.08 | 9.31 | 9.43 | 10.40 | 10.55 | 12.06 |
| **Neurological disorders (%)** |  |  |  |  |  |  |  |  |
| Alzheimer's disease and other dementias | 0.74 | 0.83 | 0.94 | 1.04 | 1.13 | 1.22 | 1.30 | 75.14 |
| Parkinson's disease | 0.16 | 0.19 | 0.21 | 0.26 | 0.23 | 0.23 | 0.25 | 57.55 |
| **Mental disorders (%)** |  |  |  |  |  |  |  |  |
| Depressive disorders | 4.71 | 4.86 | 4.92 | 5.26 | 5.11 | 4.94 | 4.72 | 0.36 |
| Anxiety disorder | 6.03 | 5.64 | 5.41 | 5.82 | 5.81 | 5.71 | 5.99 | -0.79 |
| **Substance use disorders (%)** |  |  |  |  |  |  |  |  |
| Alcohol use disorders | 1.78 | 1.92 | 1.97 | 1.81 | 1.88 | 2.01 | 2.01 | 13.28 |
| Drug use disorders | 2.39 | 2.28 | 2.10 | 2.02 | 2.07 | 2.01 | 2.03 | -14.99 |
| **Diabetes and kidney diseases (%)** |  |  |  |  |  |  |  |  |
| Diabetes mellitus type 2 | 2.25 | 3.12 | 3.71 | 4.34 | 4.54 | 5.32 | 5.32 | 136.38 |
| Chronic kidney disease | 6.95 | 7.37 | 7.98 | 8.57 | 8.99 | 9.44 | 10.12 | 45.64 |
| **Musculoskeletal disorders (%)** |  |  |  |  |  |  |  |  |
| Osteoarthritis | 8.68 | 9.34 | 10.30 | 11.21 | 11.54 | 12.14 | 13.01 | 49.80 |
| **South Korea** |  |  |  |  |  |  |  |  |
| **Neoplasms/Cancer (per 100,000 persons)** |  |  |  |  |  |  |  |  |
| Lip and oral cavity cancer | 2.92 | 4.63 | 6.46 | 7.49 | 8.71 | 8.60 | 9.50 | 225.34 |
| Colon and rectum cancer | 55.45 | 102.67 | 134.98 | 210.03 | 299.01 | 323.55 | 375.45 | 577.10 |
| Stomach cancer | 82.29 | 118.85 | 120.60 | 152.70 | 169.95 | 141.08 | 151.99 | 84.70 |
| Pancreatic cancer | 4.17 | 6.00 | 6.14 | 7.25 | 9.99 | 12.29 | 14.74 | 253.48 |
| Non-Hodgkin's lymphoma | 4.33 | 15.74 | 20.87 | 28.99 | 40.55 | 49.40 | 57.01 | 1216.63 |
| Gallbladder and biliary tract cancer | 7.75 | 9.36 | 10.38 | 12.19 | 13.89 | 15.22 | 17.86 | 130.45 |
| Kidney cancer | 4.29 | 8.75 | 13.81 | 21.35 | 29.78 | 29.79 | 32.45 | 656.41 |
| Ovarian cancer | 5.49 | 8.69 | 11.10 | 13.80 | 16.86 | 19.32 | 20.89 | 280.51 |
| Cervical cancer | 43.12 | 50.81 | 50.49 | 45.16 | 45.33 | 41.64 | 42.75 | -0.86 |
| Breast cancer | 101.38 | 120.40 | 159.34 | 232.14 | 314.21 | 367.50 | 408.43 | 302.87 |
| Oesophageal cancer | 7.03 | 10.30 | 9.86 | 11.77 | 14.62 | 16.83 | 20.12 | 186.20 |
| Tracheal, bronchus and lung cancer | 21.16 | 35.26 | 48.52 | 65.38 | 91.23 | 108.81 | 127.99 | 504.87 |
| Prostate cancer | 14.34 | 23.83 | 37.52 | 69.13 | 110.63 | 136.90 | 161.38 | 1025.38 |
| Thyroid cancer | 12.20 | 23.85 | 51.62 | 113.20 | 163.93 | 93.82 | 82.54 | 576.56 |
| Leukaemia | 35.86 | 36.59 | 30.36 | 35.24 | 44.38 | 60.94 | 67.09 | 87.09 |
| Multiple myeloma | 1.87 | 2.22 | 3.11 | 4.85 | 6.97 | 8.39 | 9.77 | 422.46 |
| **Cardiovascular diseases (%)** |  |  |  |  |  |  |  |  |
| Stroke | 1.64 | 1.76 | 1.79 | 1.77 | 1.78 | 1.86 | 1.91 | 16.41 |
| Ischemic heart disease | 0.69 | 0.82 | 0.94 | 1.01 | 1.17 | 1.31 | 1.57 | 126.41 |
| **Chronic respiratory diseases (%)** |  |  |  |  |  |  |  |  |
| Chronic obstructive pulmonary disease | 1.21 | 1.58 | 1.92 | 2.40 | 2.74 | 3.06 | 3.13 | 157.53 |
| Asthma | 3.78 | 3.97 | 3.71 | 3.84 | 3.74 | 3.51 | 3.59 | -4.96 |
| **Digestive diseases (%)** |  |  |  |  |  |  |  |  |
| Gallbladder and biliary diseases | 4.05 | 4.21 | 4.12 | 4.17 | 4.30 | 4.54 | 4.83 | 19.27 |
| Upper digestive system diseases | 6.49 | 6.67 | 6.99 | 6.72 | 8.42 | 9.85 | 9.21 | 41.95 |
| **Neurological disorders (%)** |  |  |  |  |  |  |  |  |
| Alzheimer's disease and other dementias | 0.30 | 0.38 | 0.47 | 0.60 | 0.79 | 0.99 | 1.12 | 269.81 |
| Parkinson's disease | 0.03 | 0.04 | 0.06 | 0.07 | 0.10 | 0.12 | 0.14 | 289.48 |
| **Mental disorders (%)** |  |  |  |  |  |  |  |  |
| Depressive disorders | 2.02 | 2.04 | 2.11 | 2.21 | 2.42 | 2.55 | 2.52 | 25.03 |
| Anxiety disorder | 3.38 | 3.42 | 3.68 | 3.42 | 3.61 | 3.83 | 3.49 | 3.03 |
| **Substance use disorders (%)** |  |  |  |  |  |  |  |  |
| Alcohol use disorders | 3.24 | 3.23 | 3.06 | 3.85 | 3.71 | 3.78 | 2.95 | -8.99 |
| Drug use disorders | 0.92 | 0.91 | 0.88 | 0.84 | 0.81 | 0.78 | 0.76 | -17.65 |
| **Diabetes and kidney diseases (%)** |  |  |  |  |  |  |  |  |
| Diabetes mellitus type 2 | 2.52 | 3.20 | 3.86 | 4.98 | 5.66 | 6.60 | 7.45 | 195.84 |
| Chronic kidney disease | 5.46 | 6.18 | 6.85 | 7.94 | 8.68 | 9.63 | 10.36 | 89.89 |
| **Musculoskeletal disorders (%)** |  |  |  |  |  |  |  |  |
| Osteoarthritis | 6.73 | 7.80 | 9.09 | 10.85 | 13.23 | 15.56 | 15.79 | 134.64 |
| **China** |  |  |  |  |  |  |  |  |
| **Neoplasms/Cancer (per 100,000 persons)** |  |  |  |  |  |  |  |  |
| Lip and oral cavity cancer | 3.50 | 3.89 | 4.58 | 6.31 | 9.31 | 11.22 | 12.50 | 257.14 |
| Colon and rectum cancer | 37.50 | 42.36 | 58.61 | 99.32 | 152.31 | 189.37 | 240.70 | 541.87 |
| Stomach cancer | 38.11 | 38.83 | 47.52 | 69.05 | 81.85 | 87.88 | 101.21 | 165.57 |
| Pancreatic cancer | 1.80 | 1.97 | 2.56 | 3.47 | 4.45 | 5.18 | 6.35 | 252.78 |
| Non-Hodgkin's lymphoma | 0.32 | 0.75 | 1.93 | 5.22 | 15.69 | 22.98 | 28.85 | 8915.63 |
| Gallbladder and biliary tract cancer | 1.16 | 1.17 | 1.45 | 2.33 | 2.79 | 2.99 | 3.32 | 186.21 |
| Kidney cancer | 4.24 | 4.35 | 6.21 | 11.45 | 16.84 | 19.18 | 22.89 | 439.86 |
| Ovarian cancer | 4.58 | 5.66 | 7.10 | 8.46 | 10.33 | 11.80 | 13.80 | 201.31 |
| Cervical cancer | 13.19 | 14.88 | 20.34 | 29.12 | 35.17 | 39.51 | 40.44 | 206.60 |
| Breast cancer | 65.02 | 73.57 | 95.89 | 125.71 | 170.62 | 205.38 | 248.05 | 281.50 |
| Oesophageal cancer | 21.47 | 22.94 | 27.59 | 34.74 | 32.86 | 30.70 | 35.04 | 63.20 |
| Tracheal, bronchus and lung cancer | 23.60 | 26.29 | 32.98 | 43.10 | 55.79 | 66.33 | 80.00 | 238.98 |
| Prostate cancer | 11.65 | 14.82 | 21.17 | 31.58 | 47.24 | 62.37 | 79.52 | 582.58 |
| Thyroid cancer | 5.85 | 7.47 | 9.73 | 11.85 | 16.78 | 19.06 | 21.82 | 272.99 |
| Leukaemia | 76.31 | 71.66 | 64.56 | 53.48 | 55.77 | 60.58 | 70.36 | -7.80 |
| Multiple myeloma | 1.04 | 1.20 | 1.49 | 1.88 | 2.56 | 3.23 | 3.91 | 275.96 |
| **Cardiovascular diseases (%)** |  |  |  |  |  |  |  |  |
| Stroke | 0.98 | 1.05 | 1.18 | 1.28 | 1.47 | 1.75 | 2.02 | 105.99 |
| Ischemic heart disease | 1.47 | 1.63 | 1.87 | 2.17 | 2.46 | 2.88 | 3.18 | 115.45 |
| **Chronic respiratory diseases (%)** |  |  |  |  |  |  |  |  |
| Chronic obstructive pulmonary disease | 2.34 | 2.56 | 2.73 | 2.86 | 2.86 | 3.09 | 3.18 | 35.44 |
| Asthma | 2.16 | 2.12 | 1.81 | 1.43 | 1.34 | 1.40 | 1.74 | -19.36 |
| **Digestive diseases (%)** |  |  |  |  |  |  |  |  |
| Gallbladder and biliary diseases | 3.85 | 3.85 | 3.83 | 3.84 | 4.38 | 4.45 | 4.67 | 21.20 |
| Upper digestive system diseases | 4.98 | 4.99 | 5.25 | 5.53 | 5.43 | 5.78 | 6.43 | 29.12 |
| **Neurological disorders (%)** |  |  |  |  |  |  |  |  |
| Alzheimer's disease and other dementias | 0.28 | 0.36 | 0.43 | 0.50 | 0.61 | 0.78 | 0.92 | 226.92 |
| Parkinson's disease | 0.07 | 0.08 | 0.11 | 0.13 | 0.15 | 0.18 | 0.20 | 199.57 |
| **Mental disorders (%)** |  |  |  |  |  |  |  |  |
| Depressive disorders | 2.88 | 3.12 | 3.05 | 3.21 | 3.25 | 3.45 | 3.52 | 22.01 |
| Anxiety disorder | 3.52 | 3.54 | 3.76 | 3.47 | 3.46 | 3.27 | 3.36 | -4.49 |
| **Substance use disorders (%)** |  |  |  |  |  |  |  |  |
| Alcohol use disorders | 1.27 | 1.52 | 1.36 | 1.24 | 1.32 | 1.38 | 1.46 | 14.68 |
| Drug use disorders | 0.76 | 0.77 | 0.71 | 0.77 | 0.65 | 0.64 | 0.68 | -10.67 |
| **Diabetes and kidney diseases (%)** |  |  |  |  |  |  |  |  |
| Diabetes mellitus type 2 | 2.93 | 3.46 | 3.61 | 4.82 | 5.51 | 6.51 | 6.33 | 116.09 |
| Chronic kidney disease | 6.74 | 7.12 | 7.54 | 8.52 | 9.30 | 10.61 | 10.58 | 56.96 |
| **Musculoskeletal disorders (%)** |  |  |  |  |  |  |  |  |
| Osteoarthritis | 4.37 | 4.76 | 5.24 | 6.48 | 7.42 | 8.60 | 9.34 | 113.53 |
| **Laos** |  |  |  |  |  |  |  |  |
| **Neoplasms/Cancer (per 100,000 persons)** |  |  |  |  |  |  |  |  |
| Lip and oral cavity cancer | 5.60 | 5.43 | 5.00 | 4.92 | 5.42 | 6.23 | 7.17 | 28.04 |
| Colon and rectum cancer | 15.92 | 16.61 | 17.29 | 18.78 | 21.77 | 26.43 | 31.91 | 100.44 |
| Stomach cancer | 10.67 | 9.35 | 7.82 | 6.73 | 6.17 | 5.97 | 6.19 | -41.99 |
| Pancreatic cancer | 0.90 | 0.90 | 0.90 | 0.98 | 1.11 | 1.32 | 1.55 | 72.22 |
| Non-Hodgkin's lymphoma | N.A | N.A | N.A | N.A | N.A | N.A | N.A | N.A |
| Gallbladder and biliary tract cancer | 0.91 | 0.83 | 0.73 | 0.66 | 0.66 | 0.71 | 0.78 | -14.29 |
| Kidney cancer | 3.41 | 3.42 | 3.48 | 3.71 | 4.29 | 4.99 | 5.76 | 68.91 |
| Ovarian cancer | 5.13 | 5.80 | 6.61 | 8.29 | 10.43 | 12.71 | 14.47 | 182.07 |
| Cervical cancer | 30.80 | 30.00 | 27.81 | 26.59 | 26.94 | 28.35 | 30.46 | -1.10 |
| Breast cancer | 52.30 | 53.63 | 54.68 | 58.67 | 67.52 | 78.56 | 90.12 | 72.31 |
| Oesophageal cancer | 2.87 | 2.59 | 2.24 | 1.99 | 1.94 | 2.00 | 2.16 | -24.74 |
| Tracheal, bronchus and lung cancer | 13.13 | 12.60 | 11.79 | 11.16 | 11.51 | 12.52 | 14.07 | 7.16 |
| Prostate cancer | 6.89 | 7.35 | 7.66 | 8.40 | 10.20 | 12.89 | 16.18 | 134.83 |
| Thyroid cancer | 6.35 | 7.02 | 7.37 | 8.08 | 9.57 | 11.34 | 13.29 | 109.29 |
| Leukaemia | 81.42 | 70.68 | 60.47 | 51.22 | 43.63 | 36.59 | 32.50 | -60.08 |
| Multiple myeloma | 0.84 | 0.80 | 0.75 | 0.73 | 0.79 | 0.91 | 1.04 | 23.81 |
| **Cardiovascular diseases (%)** |  |  |  |  |  |  |  |  |
| Stroke | 1.10 | 1.05 | 1.02 | 0.99 | 1.01 | 1.05 | 1.11 | 1.37 |
| Ischemic heart disease | 0.80 | 0.76 | 0.73 | 0.73 | 0.79 | 0.85 | 0.93 | 15.98 |
| **Chronic respiratory diseases (%)** |  |  |  |  |  |  |  |  |
| Chronic obstructive pulmonary disease | 1.28 | 1.29 | 1.30 | 1.36 | 1.48 | 1.66 | 1.82 | 42.50 |
| Asthma | 2.50 | 2.46 | 2.25 | 2.11 | 2.05 | 1.98 | 2.06 | -17.84 |
| **Digestive diseases (%)** |  |  |  |  |  |  |  |  |
| Gallbladder and biliary diseases | 1.28 | 1.25 | 1.21 | 1.19 | 1.19 | 1.23 | 1.29 | 0.30 |
| Upper digestive system diseases | 4.16 | 4.15 | 4.15 | 4.28 | 4.52 | 4.81 | 5.04 | 20.95 |
| **Neurological disorders (%)** |  |  |  |  |  |  |  |  |
| Alzheimer's disease and other dementias | 0.18 | 0.18 | 0.18 | 0.19 | 0.20 | 0.22 | 0.24 | 34.65 |
| Parkinson's disease | 0.03 | 0.03 | 0.04 | 0.03 | 0.04 | 0.04 | 0.05 | 34.40 |
| **Mental disorders (%)** |  |  |  |  |  |  |  |  |
| Depressive disorders | 2.24 | 2.25 | 2.15 | 2.25 | 2.29 | 2.48 | 2.56 | 14.00 |
| Anxiety disorder | 3.58 | 3.63 | 3.68 | 3.80 | 4.07 | 4.29 | 4.42 | 23.48 |
| **Substance use disorders (%)** |  |  |  |  |  |  |  |  |
| Alcohol use disorders | 0.69 | 0.71 | 0.72 | 0.73 | 0.76 | 0.79 | 0.83 | 20.37 |
| Drug use disorders | 0.43 | 0.44 | 0.46 | 0.49 | 0.51 | 0.52 | 0.52 | 21.42 |
| **Diabetes and kidney diseases (%)** |  |  |  |  |  |  |  |  |
| Diabetes mellitus type 2 | 2.06 | 2.27 | 2.47 | 2.74 | 3.12 | 3.71 | 4.29 | 108.20 |
| Chronic kidney disease | 5.82 | 5.88 | 5.99 | 6.31 | 6.91 | 7.59 | 8.19 | 40.69 |
| **Musculoskeletal disorders (%)** |  |  |  |  |  |  |  |  |
| Osteoarthritis | 1.90 | 1.86 | 1.86 | 1.96 | 2.13 | 2.38 | 2.62 | 37.97 |

N.A, not available.

# Table S6. Prevalence of noncommunicable diseases among population with hepatitis B virus infection in other countries/areas of the western pacific region, 1990-2019.

| Noncommunicable diseases | Prevalence | | | | | | | Relative change,1990-2019 (%) |
| --- | --- | --- | --- | --- | --- | --- | --- | --- |
|  | 1990 | 1995 | 2000 | 2005 | 2010 | 2015 | 2019 |  |
| **American Samoa (USA)** |  |  |  |  |  |  |  |  |
| **Neoplasms/Cancer (per 100,000 persons)** |  |  |  |  |  |  |  |  |
| Lip and oral cavity cancer | 4.22 | 4.46 | 4.01 | 5.28 | 5.89 | 6.52 | 6.49 | 53.79 |
| Colon and rectum cancer | 44.24 | 48.89 | 56.05 | 59.53 | 71.37 | 83.30 | 87.10 | 96.88 |
| Stomach cancer | 20.98 | 21.46 | 22.76 | 21.73 | 24.99 | 27.32 | 27.87 | 32.84 |
| Pancreatic cancer | 1.93 | 2.52 | 3.20 | 4.37 | 4.46 | 5.09 | 5.31 | 175.13 |
| Non-Hodgkin's lymphoma | 0.15 | 0.18 | 0.18 | 2.77 | 4.73 | 7.55 | 7.13 | 4653.33 |
| Gallbladder and biliary tract cancer | 0.92 | 0.98 | 1.13 | 1.07 | 1.13 | 1.01 | 1.07 | 16.30 |
| Kidney cancer | 5.32 | 6.32 | 7.27 | 8.62 | 8.31 | 8.48 | 8.50 | 59.77 |
| Ovarian cancer | 15.54 | 20.62 | 26.28 | 33.67 | 35.07 | 38.39 | 38.32 | 146.59 |
| Cervical cancer | 33.37 | 37.78 | 40.90 | 43.41 | 49.23 | 54.75 | 54.39 | 62.99 |
| Breast cancer | 108.27 | 120.89 | 154.27 | 174.08 | 211.29 | 258.06 | 265.90 | 145.59 |
| Oesophageal cancer | 1.35 | 1.53 | 1.68 | 2.08 | 2.37 | 2.58 | 2.62 | 94.07 |
| Tracheal, bronchus and lung cancer | 18.35 | 18.92 | 20.20 | 22.59 | 25.69 | 28.01 | 29.67 | 61.69 |
| Prostate cancer | 68.50 | 77.68 | 93.49 | 114.81 | 137.82 | 152.27 | 161.58 | 135.88 |
| Thyroid cancer | 11.48 | 13.92 | 16.49 | 22.96 | 25.10 | 27.61 | 28.40 | 147.39 |
| Leukaemia | 18.28 | 18.09 | 18.39 | 18.59 | 19.00 | 20.38 | 20.99 | 14.82 |
| Multiple myeloma | 3.02 | 3.38 | 3.62 | 3.59 | 4.20 | 4.50 | 4.74 | 56.95 |
| **Cardiovascular diseases (%)** |  |  |  |  |  |  |  |  |
| Stroke | 1.39 | 1.42 | 1.53 | 1.61 | 1.79 | 1.85 | 1.97 | 41.44 |
| Ischemic heart disease | 1.54 | 1.70 | 1.88 | 2.13 | 2.43 | 2.64 | 2.80 | 81.41 |
| **Chronic respiratory diseases (%)** |  |  |  |  |  |  |  |  |
| Chronic obstructive pulmonary disease | 1.12 | 1.15 | 1.21 | 1.27 | 1.33 | 1.38 | 1.38 | 23.69 |
| Asthma | 3.32 | 2.53 | 2.53 | 2.46 | 2.81 | 2.56 | 3.22 | -2.96 |
| **Digestive diseases (%)** |  |  |  |  |  |  |  |  |
| Gallbladder and biliary diseases | 0.85 | 0.85 | 0.90 | 0.98 | 1.05 | 1.10 | 1.10 | 29.79 |
| Upper digestive system diseases | 5.34 | 5.49 | 5.62 | 5.79 | 6.01 | 6.10 | 6.25 | 17.00 |
| **Neurological disorders (%)** |  |  |  |  |  |  |  |  |
| Alzheimer's disease and other dementias | 0.24 | 0.27 | 0.31 | 0.36 | 0.44 | 0.48 | 0.52 | 113.52 |
| Parkinson's disease | 0.08 | 0.08 | 0.09 | 0.11 | 0.12 | 0.13 | 0.14 | 78.43 |
| **Mental disorders (%)** |  |  |  |  |  |  |  |  |
| Depressive disorders | 2.65 | 2.67 | 2.70 | 2.75 | 2.84 | 2.90 | 2.98 | 12.71 |
| Anxiety disorder | 4.03 | 4.05 | 4.10 | 4.11 | 4.25 | 4.35 | 4.40 | 9.11 |
| **Substance use disorders (%)** |  |  |  |  |  |  |  |  |
| Alcohol use disorders | 0.71 | 0.72 | 0.71 | 0.70 | 0.70 | 0.69 | 0.70 | -1.69 |
| Drug use disorders | 0.84 | 0.82 | 0.80 | 0.76 | 0.74 | 0.75 | 0.78 | -7.30 |
| **Diabetes and kidney diseases (%)** |  |  |  |  |  |  |  |  |
| Diabetes mellitus type 2 | 12.46 | 14.99 | 19.11 | 22.93 | 25.85 | 28.31 | 30.47 | 144.45 |
| Chronic kidney disease | 12.05 | 13.10 | 14.25 | 15.46 | 16.85 | 17.70 | 18.44 | 53.04 |
| **Musculoskeletal disorders (%)** |  |  |  |  |  |  |  |  |
| Osteoarthritis | 3.76 | 4.15 | 4.64 | 5.38 | 5.98 | 6.40 | 6.78 | 80.24 |
| **New Zealand** |  |  |  |  |  |  |  |  |
| **Neoplasms/Cancer (per 100,000 persons)** |  |  |  |  |  |  |  |  |
| Lip and oral cavity cancer | 41.05 | 42.10 | 43.52 | 46.55 | 53.02 | 61.35 | 65.70 | 60.05 |
| Colon and rectum cancer | 433.00 | 490.21 | 521.15 | 592.56 | 657.61 | 783.25 | 896.56 | 107.06 |
| Stomach cancer | 30.94 | 34.71 | 36.79 | 38.22 | 40.27 | 45.93 | 50.69 | 63.83 |
| Pancreatic cancer | 10.93 | 12.50 | 13.22 | 16.18 | 20.11 | 24.30 | 26.97 | 146.75 |
| Non-Hodgkin's lymphoma | 164.62 | 244.94 | 279.47 | 299.03 | 305.59 | 349.80 | 397.31 | 141.35 |
| Gallbladder and biliary tract cancer | 5.26 | 5.66 | 6.30 | 7.47 | 8.69 | 10.52 | 11.39 | 116.54 |
| Kidney cancer | 54.01 | 59.85 | 65.97 | 84.25 | 103.28 | 122.62 | 135.38 | 150.66 |
| Ovarian cancer | 32.86 | 37.90 | 37.62 | 37.98 | 38.96 | 40.54 | 42.36 | 28.91 |
| Cervical cancer | 54.59 | 44.64 | 36.58 | 33.99 | 36.22 | 37.14 | 37.14 | -31.97 |
| Breast cancer | 770.68 | 827.96 | 879.70 | 992.67 | 1131.19 | 1321.80 | 1406.89 | 82.55 |
| Oesophageal cancer | 12.81 | 15.84 | 17.78 | 21.37 | 24.69 | 29.28 | 32.89 | 156.75 |
| Tracheal, bronchus and lung cancer | 64.17 | 75.44 | 80.48 | 96.82 | 114.46 | 136.45 | 147.95 | 130.56 |
| Prostate cancer | 550.33 | 671.91 | 770.85 | 814.36 | 908.55 | 1114.15 | 1311.13 | 138.24 |
| Thyroid cancer | 12.53 | 17.37 | 19.55 | 25.09 | 30.28 | 34.11 | 36.06 | 187.79 |
| Leukaemia | 83.12 | 102.39 | 113.77 | 138.20 | 145.75 | 179.29 | 193.92 | 133.30 |
| Multiple myeloma | 33.06 | 41.44 | 50.90 | 60.71 | 73.25 | 88.98 | 100.15 | 202.93 |
| **Cardiovascular diseases (%)** |  |  |  |  |  |  |  |  |
| Stroke | 1.08 | 1.05 | 1.12 | 1.15 | 1.28 | 1.39 | 1.49 | 37.53 |
| Ischemic heart disease | 3.91 | 4.09 | 4.15 | 4.50 | 5.00 | 5.83 | 6.29 | 60.97 |
| **Chronic respiratory diseases (%)** |  |  |  |  |  |  |  |  |
| Chronic obstructive pulmonary disease | 4.71 | 5.34 | 5.87 | 6.49 | 7.21 | 8.18 | 8.68 | 84.29 |
| Asthma | 15.03 | 13.98 | 10.24 | 7.60 | 6.05 | 5.52 | 5.29 | -64.81 |
| **Digestive diseases (%)** |  |  |  |  |  |  |  |  |
| Gallbladder and biliary diseases | 2.96 | 3.89 | 4.70 | 5.31 | 5.66 | 5.82 | 5.60 | 89.35 |
| Upper digestive system diseases | 11.97 | 12.78 | 13.60 | 14.41 | 15.07 | 15.70 | 16.05 | 34.05 |
| **Neurological disorders (%)** |  |  |  |  |  |  |  |  |
| Alzheimer's disease and other dementias | 0.85 | 0.98 | 1.13 | 1.29 | 1.53 | 1.84 | 2.06 | 143.03 |
| Parkinson's disease | 0.15 | 0.16 | 0.18 | 0.22 | 0.24 | 0.31 | 0.35 | 142.44 |
| **Mental disorders (%)** |  |  |  |  |  |  |  |  |
| Depressive disorders | 4.33 | 4.55 | 4.52 | 4.50 | 4.27 | 4.08 | 4.01 | -7.51 |
| Anxiety disorder | 8.13 | 8.94 | 8.88 | 8.77 | 8.42 | 8.08 | 7.95 | -2.27 |
| **Substance use disorders (%)** |  |  |  |  |  |  |  |  |
| Alcohol use disorders | 2.76 | 2.96 | 2.05 | 1.99 | 2.65 | 3.88 | 3.82 | 38.44 |
| Drug use disorders | 1.89 | 1.96 | 1.94 | 1.74 | 1.41 | 1.18 | 1.10 | -41.87 |
| **Diabetes and kidney diseases (%)** |  |  |  |  |  |  |  |  |
| Diabetes mellitus type 2 | 3.01 | 3.73 | 4.10 | 4.04 | 5.42 | 7.60 | 9.73 | 222.94 |
| Chronic kidney disease | 11.31 | 12.62 | 14.10 | 15.64 | 17.71 | 20.13 | 21.58 | 90.73 |
| **Musculoskeletal disorders (%)** |  |  |  |  |  |  |  |  |
| Osteoarthritis | 9.94 | 11.03 | 12.43 | 14.26 | 16.56 | 19.49 | 21.05 | 111.83 |
| **Brunei Darussalam** |  |  |  |  |  |  |  |  |
| **Neoplasms/Cancer (per 100,000 persons)** |  |  |  |  |  |  |  |  |
| Lip and oral cavity cancer | 10.85 | 11.28 | 12.15 | 13.91 | 16.48 | 21.67 | 26.46 | 143.87 |
| Colon and rectum cancer | 84.47 | 90.23 | 102.19 | 152.45 | 210.00 | 287.53 | 384.92 | 355.69 |
| Stomach cancer | 38.20 | 38.63 | 35.22 | 38.85 | 41.88 | 50.03 | 65.62 | 71.78 |
| Pancreatic cancer | 2.86 | 3.16 | 3.59 | 5.03 | 7.11 | 10.38 | 14.19 | 396.15 |
| Non-Hodgkin's lymphoma | 45.14 | 49.10 | 43.89 | 65.41 | 83.02 | 105.93 | 129.21 | 186.24 |
| Gallbladder and biliary tract cancer | 2.45 | 2.39 | 2.53 | 3.26 | 4.06 | 5.50 | 7.16 | 192.24 |
| Kidney cancer | 7.78 | 9.67 | 11.48 | 17.84 | 25.54 | 35.83 | 45.40 | 483.55 |
| Ovarian cancer | 15.81 | 20.09 | 25.17 | 34.86 | 44.44 | 54.63 | 62.57 | 295.76 |
| Cervical cancer | 94.62 | 97.76 | 92.51 | 109.27 | 120.47 | 133.19 | 138.79 | 46.68 |
| Breast cancer | 155.75 | 175.09 | 207.90 | 300.23 | 414.22 | 558.09 | 680.88 | 337.16 |
| Oesophageal cancer | 4.23 | 4.39 | 4.44 | 5.16 | 6.18 | 8.22 | 11.43 | 170.21 |
| Tracheal, bronchus and lung cancer | 22.63 | 23.31 | 26.80 | 36.26 | 47.02 | 63.02 | 85.82 | 279.23 |
| Prostate cancer | 18.43 | 20.61 | 27.05 | 43.50 | 61.37 | 82.75 | 122.10 | 562.51 |
| Thyroid cancer | 20.33 | 23.77 | 27.74 | 41.07 | 53.39 | 62.48 | 73.74 | 262.72 |
| Leukaemia | 32.61 | 30.73 | 29.88 | 36.83 | 45.54 | 62.73 | 76.68 | 135.14 |
| Multiple myeloma | 3.45 | 3.55 | 4.47 | 7.32 | 10.97 | 15.90 | 21.09 | 511.30 |
| **Cardiovascular diseases (%)** |  |  |  |  |  |  |  |  |
| Stroke | 1.27 | 1.32 | 1.40 | 1.52 | 1.75 | 2.03 | 2.67 | 110.31 |
| Ischemic heart disease | 0.71 | 0.79 | 0.90 | 1.04 | 1.30 | 1.58 | 2.08 | 191.75 |
| **Chronic respiratory diseases (%)** |  |  |  |  |  |  |  |  |
| Chronic obstructive pulmonary disease | 1.73 | 2.01 | 2.19 | 2.50 | 2.79 | 3.26 | 4.02 | 131.84 |
| Asthma | 4.22 | 4.33 | 4.12 | 4.10 | 4.10 | 4.36 | 4.86 | 15.12 |
| **Digestive diseases (%)** |  |  |  |  |  |  |  |  |
| Gallbladder and biliary diseases | 4.04 | 4.58 | 4.94 | 5.52 | 6.17 | 7.04 | 7.78 | 92.36 |
| Upper digestive system diseases | 6.66 | 7.59 | 8.34 | 9.01 | 9.48 | 9.91 | 10.37 | 55.67 |
| **Neurological disorders (%)** |  |  |  |  |  |  |  |  |
| Alzheimer's disease and other dementias | 0.15 | 0.17 | 0.20 | 0.24 | 0.30 | 0.40 | 0.55 | 259.17 |
| Parkinson's disease | 0.03 | 0.04 | 0.04 | 0.05 | 0.07 | 0.09 | 0.12 | 302.10 |
| **Mental disorders (%)** |  |  |  |  |  |  |  |  |
| Depressive disorders | 1.85 | 1.94 | 1.99 | 2.00 | 1.96 | 2.03 | 2.11 | 14.32 |
| Anxiety disorder | 3.36 | 3.43 | 3.42 | 3.39 | 3.34 | 3.30 | 3.25 | -3.41 |
| **Substance use disorders (%)** |  |  |  |  |  |  |  |  |
| Alcohol use disorders | 1.22 | 1.30 | 1.36 | 1.36 | 1.31 | 1.23 | 1.20 | -1.91 |
| Drug use disorders | 1.22 | 1.30 | 1.24 | 1.02 | 0.82 | 0.70 | 0.63 | -48.44 |
| **Diabetes and kidney diseases (%)** |  |  |  |  |  |  |  |  |
| Diabetes mellitus type 2 | 8.29 | 10.44 | 12.74 | 17.11 | 21.53 | 26.23 | 30.92 | 273.12 |
| Chronic kidney disease | 9.47 | 11.02 | 12.58 | 14.59 | 16.31 | 18.39 | 20.68 | 118.51 |
| **Musculoskeletal disorders (%)** |  |  |  |  |  |  |  |  |
| Osteoarthritis | 4.38 | 5.23 | 6.31 | 8.12 | 10.30 | 13.35 | 16.90 | 286.00 |
| **Niue** |  |  |  |  |  |  |  |  |
| **Neoplasms/Cancer (per 100,000 persons)** |  |  |  |  |  |  |  |  |
| Lip and oral cavity cancer | 15.69 | 18.59 | 21.66 | 25.05 | 28.27 | 30.45 | 33.22 | 111.73 |
| Colon and rectum cancer | 69.51 | 85.98 | 109.86 | 133.94 | 160.80 | 175.91 | 197.65 | 184.35 |
| Stomach cancer | 26.31 | 30.81 | 35.82 | 39.81 | 41.15 | 41.19 | 44.02 | 67.31 |
| Pancreatic cancer | 4.45 | 5.89 | 7.95 | 10.49 | 11.63 | 12.90 | 14.52 | 226.29 |
| Non-Hodgkin's lymphoma | 1.69 | 4.67 | 9.70 | 12.57 | 26.63 | 29.27 | 32.95 | 1849.70 |
| Gallbladder and biliary tract cancer | 1.88 | 2.13 | 2.44 | 2.70 | 2.86 | 2.91 | 3.12 | 65.96 |
| Kidney cancer | 10.30 | 13.41 | 17.51 | 21.00 | 23.70 | 26.11 | 29.41 | 185.53 |
| Ovarian cancer | 11.69 | 15.86 | 21.15 | 26.75 | 30.96 | 34.23 | 37.33 | 219.33 |
| Cervical cancer | 80.27 | 99.10 | 106.35 | 109.02 | 123.52 | 127.45 | 131.48 | 63.80 |
| Breast cancer | 236.69 | 289.26 | 369.83 | 451.10 | 549.39 | 598.27 | 651.51 | 175.26 |
| Oesophageal cancer | 5.19 | 6.30 | 7.67 | 8.76 | 8.87 | 9.26 | 10.21 | 96.72 |
| Tracheal, bronchus and lung cancer | 33.81 | 39.84 | 49.43 | 58.35 | 64.37 | 67.94 | 74.97 | 121.74 |
| Prostate cancer | 90.29 | 117.23 | 172.57 | 232.18 | 282.50 | 292.72 | 330.26 | 265.78 |
| Thyroid cancer | 17.47 | 24.66 | 32.46 | 38.86 | 45.32 | 49.12 | 52.59 | 201.03 |
| Leukaemia | 40.96 | 40.48 | 41.02 | 43.19 | 44.92 | 44.21 | 46.21 | 12.82 |
| Multiple myeloma | 4.97 | 6.03 | 7.22 | 8.19 | 8.84 | 9.50 | 10.55 | 112.27 |
| **Cardiovascular diseases (%)** |  |  |  |  |  |  |  |  |
| Stroke | 2.26 | 2.46 | 2.89 | 3.19 | 3.41 | 3.51 | 3.82 | 69.14 |
| Ischemic heart disease | 3.14 | 3.51 | 4.18 | 4.94 | 5.30 | 5.58 | 6.00 | 90.84 |
| **Chronic respiratory diseases (%)** |  |  |  |  |  |  |  |  |
| Chronic obstructive pulmonary disease | 4.09 | 4.50 | 5.23 | 6.10 | 6.61 | 6.88 | 7.23 | 76.76 |
| Asthma | 3.63 | 2.44 | 2.37 | 2.39 | 2.57 | 2.56 | 3.47 | -4.47 |
| **Digestive diseases (%)** |  |  |  |  |  |  |  |  |
| Gallbladder and biliary diseases | 1.07 | 1.15 | 1.30 | 1.42 | 1.48 | 1.55 | 1.63 | 53.24 |
| Upper digestive system diseases | 6.42 | 7.15 | 7.95 | 8.58 | 8.89 | 9.10 | 9.35 | 45.70 |
| **Neurological disorders (%)** |  |  |  |  |  |  |  |  |
| Alzheimer's disease and other dementias | 0.90 | 0.89 | 0.98 | 1.16 | 1.27 | 1.35 | 1.45 | 62.33 |
| Parkinson's disease | 0.20 | 0.21 | 0.25 | 0.31 | 0.34 | 0.34 | 0.37 | 82.81 |
| **Mental disorders (%)** |  |  |  |  |  |  |  |  |
| Depressive disorders | 3.52 | 3.93 | 4.26 | 4.46 | 4.56 | 4.62 | 4.69 | 33.43 |
| Anxiety disorder | 4.37 | 4.64 | 4.70 | 4.64 | 4.68 | 4.73 | 4.79 | 9.80 |
| **Substance use disorders (%)** |  |  |  |  |  |  |  |  |
| Alcohol use disorders | 0.79 | 0.90 | 0.98 | 1.05 | 1.08 | 1.07 | 1.03 | 29.47 |
| Drug use disorders | 0.76 | 0.81 | 0.82 | 0.74 | 0.63 | 0.51 | 0.46 | -39.34 |
| **Diabetes and kidney diseases (%)** |  |  |  |  |  |  |  |  |
| Diabetes mellitus type 2 | 14.12 | 18.35 | 24.71 | 31.28 | 36.61 | 42.02 | 46.89 | 231.97 |
| Chronic kidney disease | 16.76 | 19.33 | 22.97 | 26.65 | 29.12 | 30.88 | 32.80 | 95.78 |
| **Musculoskeletal disorders (%)** |  |  |  |  |  |  |  |  |
| Osteoarthritis | 6.44 | 7.50 | 9.12 | 10.80 | 11.58 | 12.60 | 13.94 | 116.26 |
| **Cambodia** |  |  |  |  |  |  |  |  |
| **Neoplasms/Cancer (per 100,000 persons)** |  |  |  |  |  |  |  |  |
| Lip and oral cavity cancer | 5.71 | 5.89 | 6.29 | 7.70 | 9.71 | 13.17 | 16.80 | 194.22 |
| Colon and rectum cancer | 14.89 | 16.02 | 18.61 | 25.13 | 33.87 | 48.35 | 64.98 | 336.40 |
| Stomach cancer | 12.24 | 11.07 | 10.35 | 10.40 | 11.19 | 12.95 | 15.06 | 23.04 |
| Pancreatic cancer | 1.03 | 1.06 | 1.18 | 1.50 | 2.00 | 2.76 | 3.58 | 247.57 |
| Non-Hodgkin's lymphoma | 0.00 | 0.00 | 0.00 | 0.00 | 0.00 | 1.73 | 0.53 | N.A |
| Gallbladder and biliary tract cancer | 0.84 | 0.79 | 0.78 | 0.85 | 1.02 | 1.32 | 1.62 | 92.86 |
| Kidney cancer | 2.82 | 2.86 | 3.06 | 3.79 | 4.74 | 6.67 | 8.94 | 217.02 |
| Ovarian cancer | 5.33 | 6.43 | 8.35 | 11.94 | 15.54 | 20.97 | 25.57 | 379.74 |
| Cervical cancer | 34.88 | 35.26 | 35.65 | 39.90 | 43.24 | 52.24 | 61.85 | 77.32 |
| Breast cancer | 39.13 | 41.48 | 48.16 | 60.83 | 76.30 | 102.20 | 130.18 | 232.69 |
| Oesophageal cancer | 3.11 | 2.93 | 2.85 | 3.02 | 3.53 | 4.38 | 5.27 | 69.45 |
| Tracheal, bronchus and lung cancer | 12.05 | 11.77 | 12.13 | 13.57 | 16.70 | 21.42 | 26.34 | 118.59 |
| Prostate cancer | 7.36 | 7.76 | 9.23 | 13.45 | 18.55 | 27.63 | 39.51 | 436.82 |
| Thyroid cancer | 7.68 | 8.73 | 10.22 | 14.06 | 17.24 | 24.02 | 31.34 | 308.07 |
| Leukaemia | 96.21 | 84.43 | 70.30 | 58.93 | 46.41 | 44.75 | 45.89 | -52.30 |
| Multiple myeloma | 1.09 | 1.10 | 1.17 | 1.41 | 1.83 | 2.57 | 3.33 | 205.50 |
| **Cardiovascular diseases (%)** |  |  |  |  |  |  |  |  |
| Stroke | 0.84 | 0.81 | 0.87 | 0.94 | 1.10 | 1.28 | 1.51 | 80.41 |
| Ischemic heart disease | 0.79 | 0.77 | 0.85 | 0.95 | 1.12 | 1.36 | 1.61 | 104.57 |
| **Chronic respiratory diseases (%)** |  |  |  |  |  |  |  |  |
| Chronic obstructive pulmonary disease | 1.03 | 1.04 | 1.12 | 1.32 | 1.54 | 1.94 | 2.34 | 125.76 |
| Asthma | 2.14 | 2.03 | 1.97 | 1.99 | 1.83 | 1.77 | 1.93 | -9.50 |
| **Digestive diseases (%)** |  |  |  |  |  |  |  |  |
| Gallbladder and biliary diseases | 1.41 | 1.39 | 1.38 | 1.43 | 1.53 | 1.71 | 1.89 | 34.15 |
| Upper digestive system diseases | 4.55 | 4.55 | 4.74 | 5.23 | 5.98 | 6.91 | 7.62 | 67.58 |
| **Neurological disorders (%)** |  |  |  |  |  |  |  |  |
| Alzheimer's disease and other dementias | 0.18 | 0.18 | 0.20 | 0.23 | 0.28 | 0.36 | 0.43 | 141.76 |
| Parkinson's disease | 0.03 | 0.03 | 0.04 | 0.04 | 0.05 | 0.07 | 0.08 | 137.73 |
| **Mental disorders (%)** |  |  |  |  |  |  |  |  |
| Depressive disorders | 2.73 | 2.73 | 2.82 | 3.03 | 3.34 | 3.71 | 3.91 | 43.44 |
| Anxiety disorder | 4.04 | 4.09 | 4.26 | 4.65 | 5.13 | 5.45 | 5.57 | 38.00 |
| **Substance use disorders (%)** |  |  |  |  |  |  |  |  |
| Alcohol use disorders | 0.64 | 0.65 | 0.68 | 0.75 | 0.88 | 1.02 | 1.13 | 78.38 |
| Drug use disorders | 0.48 | 0.47 | 0.45 | 0.51 | 0.59 | 0.64 | 0.64 | 33.59 |
| **Diabetes and kidney diseases (%)** |  |  |  |  |  |  |  |  |
| Diabetes mellitus type 2 | 1.75 | 1.90 | 2.32 | 2.89 | 4.00 | 5.98 | 8.37 | 377.07 |
| Chronic kidney disease | 8.08 | 8.23 | 8.76 | 9.95 | 11.69 | 14.06 | 16.32 | 101.98 |
| **Musculoskeletal disorders (%)** |  |  |  |  |  |  |  |  |
| Osteoarthritis | 1.93 | 1.93 | 2.11 | 2.46 | 3.00 | 3.73 | 4.42 | 128.76 |
| **Northern Mariana Islands, Commonwealth of the (USA)** |  |  |  |  |  |  |  |  |
| **Neoplasms/Cancer (per 100,000 persons)** |  |  |  |  |  |  |  |  |
| Lip and oral cavity cancer | 21.95 | 23.44 | 23.95 | 55.16 | 69.43 | 80.57 | 87.06 | 296.63 |
| Colon and rectum cancer | 75.91 | 86.86 | 83.51 | 107.14 | 141.21 | 175.59 | 221.85 | 192.25 |
| Stomach cancer | 23.28 | 25.85 | 24.58 | 23.78 | 28.65 | 34.23 | 38.77 | 66.54 |
| Pancreatic cancer | 1.92 | 2.33 | 2.48 | 5.99 | 7.83 | 9.99 | 11.70 | 509.38 |
| Non-Hodgkin's lymphoma | 8.18 | 14.75 | 15.80 | 30.94 | 34.98 | 39.27 | 48.91 | 497.92 |
| Gallbladder and biliary tract cancer | 0.75 | 0.78 | 0.74 | 1.09 | 1.35 | 1.38 | 1.65 | 120.00 |
| Kidney cancer | 17.84 | 22.17 | 23.93 | 35.34 | 34.69 | 30.92 | 33.24 | 86.32 |
| Ovarian cancer | 7.94 | 11.33 | 13.07 | 21.35 | 23.30 | 27.45 | 27.49 | 246.22 |
| Cervical cancer | 121.51 | 137.02 | 137.06 | 154.43 | 150.69 | 162.66 | 158.45 | 30.40 |
| Breast cancer | 178.66 | 223.66 | 230.89 | 301.09 | 358.42 | 464.95 | 490.77 | 174.69 |
| Oesophageal cancer | 1.68 | 1.84 | 1.88 | 3.69 | 6.01 | 7.95 | 8.83 | 425.60 |
| Tracheal, bronchus and lung cancer | 33.84 | 33.40 | 31.14 | 38.05 | 49.97 | 62.93 | 77.16 | 128.01 |
| Prostate cancer | 48.46 | 60.83 | 58.63 | 98.61 | 139.13 | 187.38 | 251.04 | 418.04 |
| Thyroid cancer | 13.51 | 18.03 | 20.51 | 31.41 | 38.95 | 44.09 | 48.91 | 262.03 |
| Leukaemia | 35.54 | 36.76 | 38.21 | 38.46 | 37.81 | 40.72 | 46.30 | 30.28 |
| Multiple myeloma | 6.03 | 6.64 | 5.93 | 6.85 | 9.56 | 11.02 | 13.92 | 130.85 |
| **Cardiovascular diseases (%)** |  |  |  |  |  |  |  |  |
| Stroke | 1.40 | 1.38 | 1.30 | 1.52 | 1.85 | 2.18 | 2.55 | 81.42 |
| Ischemic heart disease | 1.22 | 1.32 | 1.22 | 1.66 | 2.24 | 2.94 | 3.54 | 190.10 |
| **Chronic respiratory diseases (%)** |  |  |  |  |  |  |  |  |
| Chronic obstructive pulmonary disease | 0.95 | 1.00 | 0.90 | 0.97 | 1.04 | 1.18 | 1.35 | 42.37 |
| Asthma | 3.53 | 2.71 | 2.55 | 2.97 | 2.91 | 2.76 | 3.21 | -9.06 |
| **Digestive diseases (%)** |  |  |  |  |  |  |  |  |
| Gallbladder and biliary diseases | 0.91 | 0.93 | 0.93 | 1.08 | 1.22 | 1.38 | 1.45 | 59.51 |
| Upper digestive system diseases | 6.13 | 6.23 | 6.40 | 6.59 | 6.98 | 7.33 | 7.68 | 25.13 |
| **Neurological disorders (%)** |  |  |  |  |  |  |  |  |
| Alzheimer's disease and other dementias | 0.16 | 0.19 | 0.16 | 0.23 | 0.33 | 0.46 | 0.59 | 264.74 |
| Parkinson's disease | 0.07 | 0.07 | 0.06 | 0.09 | 0.12 | 0.16 | 0.20 | 186.08 |
| **Mental disorders (%)** |  |  |  |  |  |  |  |  |
| Depressive disorders | 3.06 | 3.04 | 3.15 | 3.23 | 3.41 | 3.60 | 3.73 | 21.76 |
| Anxiety disorder | 4.21 | 4.19 | 4.43 | 4.26 | 4.39 | 4.58 | 4.63 | 9.95 |
| **Substance use disorders (%)** |  |  |  |  |  |  |  |  |
| Alcohol use disorders | 1.34 | 1.34 | 1.31 | 1.32 | 1.27 | 1.17 | 1.16 | -13.54 |
| Drug use disorders | 0.97 | 0.94 | 1.04 | 0.85 | 0.76 | 0.73 | 0.75 | -23.18 |
| **Diabetes and kidney diseases (%)** |  |  |  |  |  |  |  |  |
| Diabetes mellitus type 2 | 6.81 | 7.67 | 8.60 | 11.51 | 15.03 | 18.48 | 21.75 | 219.46 |
| Chronic kidney disease | 13.59 | 14.81 | 15.25 | 17.35 | 19.63 | 21.95 | 23.32 | 71.55 |
| **Musculoskeletal disorders (%)** |  |  |  |  |  |  |  |  |
| Osteoarthritis | 3.39 | 3.56 | 3.35 | 4.86 | 6.51 | 8.36 | 9.71 | 186.47 |
| **Palau** |  |  |  |  |  |  |  |  |
| **Neoplasms/Cancer (per 100,000 persons)** |  |  |  |  |  |  |  |  |
| Lip and oral cavity cancer | 111.84 | 127.15 | 133.85 | 147.94 | 173.18 | 197.86 | 222.28 | 98.75 |
| Colon and rectum cancer | 55.44 | 65.47 | 71.72 | 86.13 | 108.37 | 129.91 | 156.93 | 183.06 |
| Stomach cancer | 22.35 | 24.97 | 28.79 | 31.33 | 36.00 | 39.80 | 44.11 | 97.36 |
| Pancreatic cancer | 6.29 | 7.77 | 8.64 | 11.32 | 13.53 | 16.36 | 19.76 | 214.15 |
| Non-Hodgkin's lymphoma | 1.50 | 4.71 | 7.18 | 7.09 | 7.81 | 8.79 | 13.37 | 791.33 |
| Gallbladder and biliary tract cancer | 0.82 | 0.88 | 0.93 | 1.08 | 1.31 | 1.51 | 1.74 | 112.20 |
| Kidney cancer | 5.62 | 7.25 | 9.20 | 11.25 | 12.56 | 14.61 | 17.51 | 211.57 |
| Ovarian cancer | 6.06 | 8.16 | 9.47 | 12.50 | 14.65 | 16.89 | 19.14 | 215.84 |
| Cervical cancer | 208.55 | 251.46 | 244.88 | 268.39 | 308.11 | 328.00 | 340.63 | 63.33 |
| Breast cancer | 220.61 | 269.28 | 294.83 | 371.01 | 467.23 | 544.89 | 623.00 | 182.40 |
| Oesophageal cancer | 3.33 | 3.91 | 4.51 | 5.29 | 6.16 | 7.19 | 8.40 | 152.25 |
| Tracheal, bronchus and lung cancer | 36.80 | 40.32 | 44.16 | 51.34 | 63.75 | 75.67 | 89.02 | 141.90 |
| Prostate cancer | 98.01 | 114.85 | 128.91 | 142.63 | 181.94 | 225.83 | 283.92 | 189.68 |
| Thyroid cancer | 30.12 | 40.04 | 52.16 | 63.24 | 70.02 | 78.80 | 87.97 | 192.07 |
| Leukaemia | 14.26 | 13.89 | 14.21 | 15.91 | 18.43 | 20.14 | 22.59 | 58.42 |
| Multiple myeloma | 1.86 | 2.08 | 2.26 | 2.70 | 3.31 | 3.99 | 4.83 | 159.68 |
| **Cardiovascular diseases (%)** |  |  |  |  |  |  |  |  |
| Stroke | 1.73 | 1.86 | 2.02 | 2.26 | 2.68 | 3.01 | 3.47 | 100.95 |
| Ischemic heart disease | 1.94 | 2.16 | 2.28 | 2.66 | 3.20 | 3.74 | 4.27 | 120.79 |
| **Chronic respiratory diseases (%)** |  |  |  |  |  |  |  |  |
| Chronic obstructive pulmonary disease | 2.84 | 3.11 | 3.23 | 3.67 | 4.21 | 4.75 | 5.29 | 86.09 |
| Asthma | 3.89 | 3.36 | 3.18 | 2.99 | 2.82 | 2.81 | 3.12 | -19.80 |
| **Digestive diseases (%)** |  |  |  |  |  |  |  |  |
| Gallbladder and biliary diseases | 0.93 | 1.02 | 1.05 | 1.18 | 1.29 | 1.41 | 1.53 | 63.52 |
| Upper digestive system diseases | 6.14 | 6.88 | 7.38 | 7.93 | 8.38 | 8.75 | 9.05 | 47.30 |
| **Neurological disorders (%)** |  |  |  |  |  |  |  |  |
| Alzheimer's disease and other dementias | 0.34 | 0.37 | 0.37 | 0.43 | 0.52 | 0.60 | 0.68 | 98.88 |
| Parkinson's disease | 0.12 | 0.13 | 0.13 | 0.15 | 0.18 | 0.21 | 0.24 | 97.49 |
| **Mental disorders (%)** |  |  |  |  |  |  |  |  |
| Depressive disorders | 3.50 | 3.88 | 4.07 | 4.30 | 4.45 | 4.52 | 4.59 | 31.45 |
| Anxiety disorder | 4.34 | 4.52 | 4.47 | 4.49 | 4.55 | 4.58 | 4.64 | 6.96 |
| **Substance use disorders (%)** |  |  |  |  |  |  |  |  |
| Alcohol use disorders | 0.90 | 1.03 | 1.21 | 1.26 | 1.24 | 1.22 | 1.18 | 31.78 |
| Drug use disorders | 0.92 | 0.94 | 0.97 | 0.91 | 0.76 | 0.60 | 0.51 | -44.13 |
| **Diabetes and kidney diseases (%)** |  |  |  |  |  |  |  |  |
| Diabetes mellitus type 2 | 9.03 | 11.68 | 14.98 | 19.59 | 24.29 | 29.72 | 34.52 | 282.36 |
| Chronic kidney disease | 14.49 | 17.27 | 19.73 | 22.29 | 25.07 | 26.64 | 28.75 | 98.32 |
| **Musculoskeletal disorders (%)** |  |  |  |  |  |  |  |  |
| Osteoarthritis | 4.46 | 5.11 | 5.64 | 7.07 | 8.42 | 10.00 | 11.65 | 161.13 |
| **Cook Islands** |  |  |  |  |  |  |  |  |
| **Neoplasms/Cancer (per 100,000 persons)** |  |  |  |  |  |  |  |  |
| Lip and oral cavity cancer | 10.41 | 11.11 | 12.00 | 13.54 | 17.37 | 21.50 | 24.69 | 137.18 |
| Colon and rectum cancer | 40.58 | 48.54 | 58.58 | 63.42 | 82.09 | 106.13 | 130.73 | 222.15 |
| Stomach cancer | 17.46 | 18.65 | 20.69 | 20.94 | 24.57 | 29.38 | 32.36 | 85.34 |
| Pancreatic cancer | 3.25 | 4.22 | 5.58 | 6.22 | 7.34 | 9.61 | 11.33 | 248.62 |
| Non-Hodgkin's lymphoma | 0.49 | 2.86 | 4.86 | 9.16 | 18.22 | 27.62 | 37.35 | 7522.45 |
| Gallbladder and biliary tract cancer | 1.61 | 1.71 | 2.00 | 1.77 | 2.07 | 2.24 | 2.54 | 57.76 |
| Kidney cancer | 7.10 | 8.28 | 9.89 | 11.68 | 13.92 | 16.95 | 20.27 | 185.49 |
| Ovarian cancer | 5.46 | 6.92 | 8.91 | 12.14 | 16.02 | 20.30 | 22.85 | 318.50 |
| Cervical cancer | 31.63 | 30.26 | 29.16 | 34.72 | 41.87 | 46.32 | 48.52 | 53.40 |
| Breast cancer | 301.73 | 349.94 | 417.23 | 476.49 | 625.34 | 762.08 | 862.24 | 185.77 |
| Oesophageal cancer | 4.96 | 5.85 | 6.84 | 7.69 | 8.58 | 9.96 | 11.33 | 128.43 |
| Tracheal, bronchus and lung cancer | 32.95 | 36.51 | 41.98 | 46.85 | 53.94 | 62.88 | 73.36 | 122.64 |
| Prostate cancer | 235.79 | 310.93 | 389.74 | 462.31 | 577.88 | 680.52 | 830.02 | 252.02 |
| Thyroid cancer | 19.76 | 23.29 | 29.90 | 26.46 | 26.00 | 33.29 | 38.08 | 92.71 |
| Leukaemia | 22.59 | 20.64 | 20.01 | 20.68 | 23.10 | 25.85 | 28.64 | 26.78 |
| Multiple myeloma | 2.51 | 2.89 | 3.36 | 3.82 | 4.74 | 5.85 | 6.87 | 173.71 |
| **Cardiovascular diseases (%)** |  |  |  |  |  |  |  |  |
| Stroke | 1.68 | 1.86 | 2.18 | 2.40 | 2.84 | 3.27 | 3.76 | 123.84 |
| Ischemic heart disease | 2.18 | 2.61 | 3.15 | 3.67 | 4.48 | 5.35 | 6.04 | 177.31 |
| **Chronic respiratory diseases (%)** |  |  |  |  |  |  |  |  |
| Chronic obstructive pulmonary disease | 3.11 | 3.60 | 4.18 | 4.78 | 5.69 | 6.64 | 7.43 | 138.70 |
| Asthma | 3.84 | 2.64 | 2.65 | 2.60 | 2.70 | 2.71 | 3.32 | -13.60 |
| **Digestive diseases (%)** |  |  |  |  |  |  |  |  |
| Gallbladder and biliary diseases | 0.86 | 0.92 | 1.03 | 1.13 | 1.25 | 1.38 | 1.48 | 71.40 |
| Upper digestive system diseases | 5.88 | 6.48 | 7.23 | 7.81 | 8.42 | 8.90 | 9.22 | 56.98 |
| **Neurological disorders (%)** |  |  |  |  |  |  |  |  |
| Alzheimer's disease and other dementias | 0.41 | 0.49 | 0.59 | 0.72 | 0.93 | 1.17 | 1.37 | 230.62 |
| Parkinson's disease | 0.13 | 0.14 | 0.17 | 0.21 | 0.26 | 0.31 | 0.35 | 177.07 |
| **Mental disorders (%)** |  |  |  |  |  |  |  |  |
| Depressive disorders | 3.38 | 3.70 | 4.02 | 4.25 | 4.46 | 4.59 | 4.66 | 38.04 |
| Anxiety disorder | 4.25 | 4.45 | 4.57 | 4.59 | 4.69 | 4.77 | 4.83 | 13.55 |
| **Substance use disorders (%)** |  |  |  |  |  |  |  |  |
| Alcohol use disorders | 0.83 | 0.91 | 1.02 | 1.09 | 1.11 | 1.08 | 1.03 | 24.43 |
| Drug use disorders | 0.87 | 0.90 | 0.91 | 0.86 | 0.73 | 0.59 | 0.52 | -40.01 |
| **Diabetes and kidney diseases (%)** |  |  |  |  |  |  |  |  |
| Diabetes mellitus type 2 | 9.06 | 11.95 | 15.95 | 20.13 | 25.49 | 31.22 | 35.84 | 295.49 |
| Chronic kidney disease | 13.41 | 15.68 | 18.69 | 21.42 | 24.98 | 28.34 | 31.24 | 133.01 |
| **Musculoskeletal disorders (%)** |  |  |  |  |  |  |  |  |
| Osteoarthritis | 4.90 | 5.90 | 7.24 | 8.57 | 10.18 | 12.12 | 13.83 | 182.28 |
| **Papua New Guinea** |  |  |  |  |  |  |  |  |
| **Neoplasms/Cancer (per 100,000 persons)** |  |  |  |  |  |  |  |  |
| Lip and oral cavity cancer | 4.47 | 4.58 | 4.86 | 5.36 | 6.16 | 7.14 | 7.78 | 74.05 |
| Colon and rectum cancer | 12.15 | 12.84 | 13.86 | 14.95 | 17.13 | 20.18 | 22.74 | 87.16 |
| Stomach cancer | 13.25 | 13.40 | 14.27 | 15.73 | 17.24 | 18.40 | 19.05 | 43.77 |
| Pancreatic cancer | 0.70 | 0.76 | 0.85 | 0.98 | 1.11 | 1.30 | 1.47 | 110.00 |
| Non-Hodgkin's lymphoma | 0.00 | 0.00 | 0.00 | 0.00 | 0.00 | 0.00 | 0.00 | N.A |
| Gallbladder and biliary tract cancer | 0.58 | 0.58 | 0.61 | 0.65 | 0.72 | 0.80 | 0.85 | 46.55 |
| Kidney cancer | 1.83 | 1.91 | 1.97 | 1.94 | 2.11 | 2.45 | 2.85 | 55.74 |
| Ovarian cancer | 3.05 | 3.49 | 4.23 | 5.04 | 5.86 | 7.03 | 7.79 | 155.41 |
| Cervical cancer | 34.27 | 35.58 | 39.86 | 46.13 | 52.31 | 58.09 | 61.34 | 78.99 |
| Breast cancer | 125.72 | 136.12 | 153.47 | 174.22 | 202.50 | 235.47 | 258.75 | 105.81 |
| Oesophageal cancer | 1.86 | 1.87 | 1.96 | 2.10 | 2.30 | 2.55 | 2.72 | 46.24 |
| Tracheal, bronchus and lung cancer | 11.32 | 11.47 | 12.24 | 13.39 | 15.23 | 17.38 | 18.88 | 66.78 |
| Prostate cancer | 13.74 | 15.23 | 17.15 | 19.07 | 22.37 | 27.22 | 31.37 | 128.31 |
| Thyroid cancer | 3.49 | 4.02 | 4.95 | 5.78 | 6.47 | 7.44 | 8.31 | 138.11 |
| Leukaemia | 43.29 | 40.34 | 38.89 | 36.03 | 34.94 | 34.48 | 36.46 | -15.78 |
| Multiple myeloma | 1.35 | 1.34 | 1.38 | 1.48 | 1.68 | 1.93 | 2.13 | 57.78 |
| **Cardiovascular diseases (%)** |  |  |  |  |  |  |  |  |
| Stroke | 1.20 | 1.21 | 1.27 | 1.37 | 1.51 | 1.65 | 1.72 | 43.62 |
| Ischemic heart disease | 1.33 | 1.39 | 1.48 | 1.60 | 1.76 | 1.93 | 2.05 | 54.05 |
| **Chronic respiratory diseases (%)** |  |  |  |  |  |  |  |  |
| Chronic obstructive pulmonary disease | 1.92 | 1.97 | 2.05 | 2.17 | 2.35 | 2.56 | 2.69 | 40.40 |
| Asthma | 5.52 | 5.44 | 5.36 | 5.10 | 4.63 | 4.50 | 4.51 | -18.40 |
| **Digestive diseases (%)** |  |  |  |  |  |  |  |  |
| Gallbladder and biliary diseases | 0.87 | 0.86 | 0.90 | 0.97 | 1.04 | 1.11 | 1.14 | 31.42 |
| Upper digestive system diseases | 5.01 | 5.11 | 5.36 | 5.76 | 6.20 | 6.55 | 6.64 | 32.55 |
| **Neurological disorders (%)** |  |  |  |  |  |  |  |  |
| Alzheimer's disease and other dementias | 0.20 | 0.21 | 0.23 | 0.25 | 0.26 | 0.28 | 0.29 | 48.27 |
| Parkinson's disease | 0.05 | 0.05 | 0.06 | 0.06 | 0.06 | 0.07 | 0.07 | 41.36 |
| **Mental disorders (%)** |  |  |  |  |  |  |  |  |
| Depressive disorders | 2.98 | 3.05 | 3.17 | 3.37 | 3.55 | 3.65 | 3.60 | 20.87 |
| Anxiety disorder | 3.88 | 3.97 | 4.11 | 4.24 | 4.35 | 4.33 | 4.27 | 9.85 |
| **Substance use disorders (%)** |  |  |  |  |  |  |  |  |
| Alcohol use disorders | 0.74 | 0.76 | 0.81 | 0.87 | 0.94 | 0.99 | 0.99 | 33.33 |
| Drug use disorders | 0.75 | 0.77 | 0.81 | 0.86 | 0.88 | 0.83 | 0.75 | 0.35 |
| **Diabetes and kidney diseases (%)** |  |  |  |  |  |  |  |  |
| Diabetes mellitus type 2 | 6.29 | 7.02 | 8.27 | 9.59 | 11.50 | 13.78 | 15.20 | 141.75 |
| Chronic kidney disease | 8.80 | 9.18 | 9.81 | 10.67 | 11.74 | 12.82 | 13.44 | 52.76 |
| **Musculoskeletal disorders (%)** |  |  |  |  |  |  |  |  |
| Osteoarthritis | 2.89 | 2.94 | 3.09 | 3.38 | 3.76 | 4.26 | 4.61 | 59.27 |
| **Fiji** |  |  |  |  |  |  |  |  |
| **Neoplasms/Cancer (per 100,000 persons)** |  |  |  |  |  |  |  |  |
| Lip and oral cavity cancer | 10.10 | 10.36 | 10.83 | 12.70 | 14.59 | 17.38 | 20.22 | 100.20 |
| Colon and rectum cancer | 23.03 | 25.81 | 29.58 | 35.62 | 47.71 | 60.81 | 74.06 | 221.58 |
| Stomach cancer | 8.56 | 8.91 | 9.43 | 10.28 | 12.99 | 14.99 | 16.69 | 94.98 |
| Pancreatic cancer | 1.54 | 1.89 | 2.42 | 3.13 | 3.90 | 4.98 | 6.18 | 301.30 |
| Non-Hodgkin's lymphoma | 0.00 | 0.00 | 0.05 | 0.06 | 0.08 | 0.12 | 0.53 | N.A |
| Gallbladder and biliary tract cancer | 0.88 | 0.95 | 1.05 | 1.27 | 1.71 | 2.02 | 2.33 | 164.77 |
| Kidney cancer | 3.69 | 4.03 | 4.65 | 5.02 | 5.93 | 7.15 | 8.86 | 140.11 |
| Ovarian cancer | 3.67 | 4.90 | 6.29 | 7.30 | 8.05 | 8.77 | 9.97 | 171.66 |
| Cervical cancer | 96.87 | 98.39 | 99.69 | 115.72 | 142.14 | 155.07 | 166.04 | 71.40 |
| Breast cancer | 135.85 | 153.73 | 176.78 | 213.91 | 285.03 | 338.71 | 395.30 | 190.98 |
| Oesophageal cancer | 2.41 | 2.61 | 2.88 | 3.58 | 4.96 | 5.97 | 6.90 | 186.31 |
| Tracheal, bronchus and lung cancer | 6.55 | 7.15 | 8.04 | 9.21 | 11.20 | 13.70 | 16.06 | 145.19 |
| Prostate cancer | 24.18 | 26.03 | 28.94 | 38.86 | 52.79 | 67.69 | 85.00 | 251.53 |
| Thyroid cancer | 18.78 | 20.86 | 23.55 | 25.32 | 30.64 | 35.93 | 42.41 | 125.83 |
| Leukaemia | 37.02 | 35.89 | 35.84 | 30.58 | 29.77 | 30.06 | 31.51 | -14.88 |
| Multiple myeloma | 1.78 | 2.02 | 2.32 | 2.77 | 3.68 | 4.36 | 5.04 | 183.15 |
| **Cardiovascular diseases (%)** |  |  |  |  |  |  |  |  |
| Stroke | 1.28 | 1.42 | 1.64 | 1.81 | 2.08 | 2.32 | 2.65 | 106.66 |
| Ischemic heart disease | 1.52 | 1.76 | 2.02 | 2.45 | 2.97 | 3.51 | 4.00 | 163.33 |
| **Chronic respiratory diseases (%)** |  |  |  |  |  |  |  |  |
| Chronic obstructive pulmonary disease | 0.62 | 0.63 | 0.64 | 0.67 | 0.70 | 0.76 | 0.86 | 38.39 |
| Asthma | 4.40 | 4.32 | 4.09 | 3.85 | 3.52 | 3.43 | 3.66 | -16.86 |
| **Digestive diseases (%)** |  |  |  |  |  |  |  |  |
| Gallbladder and biliary diseases | 0.85 | 0.92 | 1.01 | 1.14 | 1.26 | 1.40 | 1.50 | 76.26 |
| Upper digestive system diseases | 5.39 | 5.96 | 6.58 | 7.22 | 7.67 | 8.00 | 8.27 | 53.36 |
| **Neurological disorders (%)** |  |  |  |  |  |  |  |  |
| Alzheimer's disease and other dementias | 0.21 | 0.25 | 0.27 | 0.33 | 0.41 | 0.51 | 0.62 | 191.13 |
| Parkinson's disease | 0.07 | 0.08 | 0.09 | 0.11 | 0.13 | 0.16 | 0.18 | 159.47 |
| **Mental disorders (%)** |  |  |  |  |  |  |  |  |
| Depressive disorders | 2.92 | 3.24 | 3.56 | 3.80 | 3.89 | 3.99 | 4.09 | 40.05 |
| Anxiety disorder | 4.16 | 4.43 | 4.53 | 4.51 | 4.54 | 4.62 | 4.68 | 12.53 |
| **Substance use disorders (%)** |  |  |  |  |  |  |  |  |
| Alcohol use disorders | 0.64 | 0.69 | 0.77 | 0.82 | 0.85 | 0.79 | 0.79 | 24.65 |
| Drug use disorders | 0.84 | 0.89 | 0.95 | 0.89 | 0.76 | 0.63 | 0.59 | -30.00 |
| **Diabetes and kidney diseases (%)** |  |  |  |  |  |  |  |  |
| Diabetes mellitus type 2 | 9.04 | 12.38 | 15.99 | 21.39 | 26.78 | 31.43 | 35.86 | 296.78 |
| Chronic kidney disease | 12.03 | 13.86 | 15.75 | 17.91 | 20.26 | 22.47 | 24.29 | 101.90 |
| **Musculoskeletal disorders (%)** |  |  |  |  |  |  |  |  |
| Osteoarthritis | 3.32 | 4.02 | 4.84 | 5.93 | 7.01 | 8.28 | 9.48 | 185.81 |
| **Philippines** |  |  |  |  |  |  |  |  |
| **Neoplasms/Cancer (per 100,000 persons)** |  |  |  |  |  |  |  |  |
| Lip and oral cavity cancer | 9.11 | 10.01 | 9.27 | 8.94 | 9.72 | 11.06 | 13.60 | 49.29 |
| Colon and rectum cancer | 29.82 | 34.39 | 36.02 | 39.94 | 50.07 | 65.76 | 84.11 | 182.06 |
| Stomach cancer | 6.14 | 6.39 | 5.85 | 5.91 | 6.42 | 7.05 | 8.07 | 31.43 |
| Pancreatic cancer | 1.62 | 1.94 | 2.04 | 2.23 | 2.59 | 3.53 | 4.51 | 178.40 |
| Non-Hodgkin's lymphoma | 3.89 | 3.32 | 2.92 | 2.72 | 2.78 | 3.58 | 5.04 | 29.56 |
| Gallbladder and biliary tract cancer | 0.49 | 0.53 | 0.52 | 0.55 | 0.63 | 0.73 | 0.88 | 79.59 |
| Kidney cancer | 6.81 | 6.89 | 7.25 | 7.54 | 8.64 | 11.47 | 13.76 | 102.06 |
| Ovarian cancer | 12.20 | 14.63 | 15.99 | 17.95 | 21.29 | 25.27 | 29.94 | 145.41 |
| Cervical cancer | 30.51 | 32.35 | 31.96 | 34.15 | 39.57 | 44.88 | 50.93 | 66.93 |
| Breast cancer | 85.75 | 97.50 | 103.92 | 115.74 | 140.33 | 178.60 | 219.36 | 155.81 |
| Oesophageal cancer | 1.45 | 1.61 | 1.59 | 1.69 | 1.92 | 2.25 | 2.67 | 84.14 |
| Tracheal, bronchus and lung cancer | 12.14 | 14.08 | 13.84 | 13.41 | 14.33 | 16.38 | 20.29 | 67.13 |
| Prostate cancer | 17.56 | 21.92 | 23.00 | 24.76 | 30.25 | 38.47 | 50.23 | 186.05 |
| Thyroid cancer | 19.59 | 22.47 | 22.98 | 24.12 | 27.98 | 33.90 | 41.05 | 109.55 |
| Leukaemia | 63.69 | 51.11 | 44.56 | 40.43 | 37.84 | 39.32 | 36.35 | -42.93 |
| Multiple myeloma | 1.03 | 1.16 | 1.15 | 1.25 | 1.53 | 1.89 | 2.32 | 125.24 |
| **Cardiovascular diseases (%)** |  |  |  |  |  |  |  |  |
| Stroke | 0.90 | 0.94 | 1.00 | 1.13 | 1.32 | 1.46 | 1.72 | 90.97 |
| Ischemic heart disease | 1.12 | 1.25 | 1.49 | 1.66 | 1.83 | 2.08 | 2.52 | 124.83 |
| **Chronic respiratory diseases (%)** |  |  |  |  |  |  |  |  |
| Chronic obstructive pulmonary disease | 1.19 | 1.34 | 1.49 | 1.69 | 1.98 | 2.21 | 2.58 | 116.92 |
| Asthma | 5.99 | 5.54 | 4.92 | 4.64 | 4.21 | 4.10 | 3.89 | -35.10 |
| **Digestive diseases (%)** |  |  |  |  |  |  |  |  |
| Gallbladder and biliary diseases | 1.16 | 1.21 | 1.23 | 1.33 | 1.71 | 1.84 | 2.06 | 76.71 |
| Upper digestive system diseases | 4.88 | 5.15 | 5.45 | 5.95 | 6.59 | 6.95 | 7.70 | 57.91 |
| **Neurological disorders (%)** |  |  |  |  |  |  |  |  |
| Alzheimer's disease and other dementias | 0.19 | 0.21 | 0.23 | 0.26 | 0.31 | 0.36 | 0.45 | 140.34 |
| Parkinson's disease | 0.04 | 0.04 | 0.04 | 0.05 | 0.06 | 0.07 | 0.09 | 151.99 |
| **Mental disorders (%)** |  |  |  |  |  |  |  |  |
| Depressive disorders | 2.65 | 2.71 | 2.70 | 2.84 | 3.11 | 3.26 | 3.59 | 35.19 |
| Anxiety disorder | 4.25 | 4.47 | 4.68 | 4.87 | 5.15 | 5.16 | 5.47 | 28.88 |
| **Substance use disorders (%)** |  |  |  |  |  |  |  |  |
| Alcohol use disorders | 0.94 | 0.94 | 0.89 | 0.88 | 0.96 | 0.98 | 1.08 | 14.45 |
| Drug use disorders | 0.66 | 0.67 | 0.67 | 0.69 | 0.71 | 0.67 | 0.67 | 0.85 |
| **Diabetes and kidney diseases (%)** |  |  |  |  |  |  |  |  |
| Diabetes mellitus type 2 | 2.73 | 3.11 | 3.47 | 4.08 | 4.39 | 5.10 | 6.75 | 147.60 |
| Chronic kidney disease | 9.45 | 10.40 | 11.43 | 12.86 | 14.79 | 16.44 | 19.23 | 103.45 |
| **Musculoskeletal disorders (%)** |  |  |  |  |  |  |  |  |
| Osteoarthritis | 1.85 | 2.03 | 2.29 | 2.66 | 3.17 | 3.70 | 4.52 | 144.06 |
| **Guam (USA)** |  |  |  |  |  |  |  |  |
| **Neoplasms/Cancer (per 100,000 persons)** |  |  |  |  |  |  |  |  |
| Lip and oral cavity cancer | 8.96 | 10.65 | 11.16 | 12.99 | 14.93 | 17.49 | 18.07 | 101.67 |
| Colon and rectum cancer | 70.49 | 78.08 | 94.22 | 92.06 | 104.88 | 120.91 | 132.75 | 88.32 |
| Stomach cancer | 11.05 | 12.46 | 14.15 | 12.48 | 13.88 | 15.30 | 16.06 | 45.34 |
| Pancreatic cancer | 2.97 | 3.14 | 4.11 | 5.15 | 5.99 | 6.85 | 7.45 | 150.84 |
| Non-Hodgkin's lymphoma | 22.14 | 34.43 | 30.38 | 23.06 | 26.53 | 32.85 | 35.96 | 62.42 |
| Gallbladder and biliary tract cancer | 1.02 | 1.09 | 1.13 | 0.97 | 0.99 | 0.88 | 0.97 | -4.90 |
| Kidney cancer | 17.49 | 20.42 | 21.76 | 26.60 | 24.64 | 20.55 | 21.17 | 21.04 |
| Ovarian cancer | 8.28 | 10.12 | 15.80 | 18.62 | 19.37 | 20.28 | 20.05 | 142.15 |
| Cervical cancer | 36.94 | 47.56 | 45.65 | 44.32 | 46.36 | 48.18 | 48.13 | 30.29 |
| Breast cancer | 116.17 | 134.04 | 172.78 | 179.93 | 204.88 | 228.86 | 239.88 | 106.49 |
| Oesophageal cancer | 3.51 | 3.67 | 3.65 | 4.56 | 5.57 | 6.72 | 6.94 | 97.72 |
| Tracheal, bronchus and lung cancer | 30.31 | 31.58 | 32.48 | 34.91 | 40.11 | 44.94 | 48.38 | 59.62 |
| Prostate cancer | 59.74 | 74.57 | 87.01 | 93.91 | 103.80 | 113.21 | 125.37 | 109.86 |
| Thyroid cancer | 9.38 | 11.50 | 21.51 | 27.77 | 27.89 | 31.09 | 32.55 | 247.01 |
| Leukaemia | 25.13 | 26.73 | 27.62 | 27.15 | 28.85 | 31.14 | 32.96 | 31.16 |
| Multiple myeloma | 3.85 | 4.32 | 4.57 | 4.26 | 4.95 | 5.06 | 5.72 | 48.57 |
| **Cardiovascular diseases (%)** |  |  |  |  |  |  |  |  |
| Stroke | 1.46 | 1.59 | 1.78 | 1.96 | 2.23 | 2.38 | 2.52 | 73.04 |
| Ischemic heart disease | 1.59 | 1.91 | 2.27 | 2.66 | 3.11 | 3.47 | 3.70 | 132.12 |
| **Chronic respiratory diseases (%)** |  |  |  |  |  |  |  |  |
| Chronic obstructive pulmonary disease | 0.94 | 1.05 | 1.18 | 1.24 | 1.26 | 1.30 | 1.37 | 45.18 |
| Asthma | 3.69 | 3.18 | 3.20 | 3.03 | 2.78 | 2.67 | 3.11 | -15.72 |
| **Digestive diseases (%)** |  |  |  |  |  |  |  |  |
| Gallbladder and biliary diseases | 0.76 | 0.76 | 0.79 | 0.83 | 0.86 | 0.89 | 0.94 | 23.88 |
| Upper digestive system diseases | 6.03 | 6.23 | 6.37 | 6.56 | 6.78 | 6.86 | 7.00 | 16.06 |
| **Neurological disorders (%)** |  |  |  |  |  |  |  |  |
| Alzheimer's disease and other dementias | 0.28 | 0.34 | 0.42 | 0.52 | 0.66 | 0.78 | 0.87 | 210.88 |
| Parkinson's disease | 0.09 | 0.10 | 0.12 | 0.14 | 0.15 | 0.17 | 0.18 | 92.31 |
| **Mental disorders (%)** |  |  |  |  |  |  |  |  |
| Depressive disorders | 3.55 | 3.58 | 3.56 | 3.64 | 3.78 | 3.82 | 3.84 | 7.98 |
| Anxiety disorder | 4.16 | 4.19 | 4.25 | 4.29 | 4.39 | 4.42 | 4.44 | 6.72 |
| **Substance use disorders (%)** |  |  |  |  |  |  |  |  |
| Alcohol use disorders | 0.89 | 0.90 | 0.88 | 0.90 | 0.91 | 0.91 | 0.91 | 1.82 |
| Drug use disorders | 0.94 | 0.89 | 0.84 | 0.81 | 0.78 | 0.77 | 0.76 | -18.80 |
| **Diabetes and kidney diseases (%)** |  |  |  |  |  |  |  |  |
| Diabetes mellitus type 2 | 6.46 | 7.26 | 8.29 | 9.62 | 11.28 | 12.89 | 14.67 | 127.06 |
| Chronic kidney disease | 13.05 | 14.44 | 16.00 | 17.31 | 18.57 | 19.47 | 20.54 | 57.41 |
| **Musculoskeletal disorders (%)** |  |  |  |  |  |  |  |  |
| Osteoarthritis | 4.38 | 4.94 | 5.65 | 6.47 | 7.35 | 7.98 | 8.45 | 92.82 |
| **Samoa** |  |  |  |  |  |  |  |  |
| **Neoplasms/Cancer (per 100,000 persons)** |  |  |  |  |  |  |  |  |
| Lip and oral cavity cancer | 7.12 | 7.50 | 7.85 | 8.41 | 8.95 | 8.95 | 9.77 | 37.22 |
| Colon and rectum cancer | 31.67 | 35.32 | 39.67 | 44.08 | 47.85 | 47.80 | 53.60 | 69.25 |
| Stomach cancer | 18.76 | 19.61 | 19.91 | 20.69 | 21.66 | 21.33 | 21.74 | 15.88 |
| Pancreatic cancer | 3.01 | 3.51 | 3.94 | 4.29 | 4.05 | 4.05 | 4.47 | 48.50 |
| Non-Hodgkin's lymphoma | 0.07 | 0.08 | 0.10 | 0.14 | 0.41 | 0.39 | 1.48 | 2014.29 |
| Gallbladder and biliary tract cancer | 1.11 | 1.20 | 1.26 | 1.34 | 1.41 | 1.38 | 1.41 | 27.03 |
| Kidney cancer | 5.32 | 5.79 | 6.64 | 7.12 | 7.05 | 6.91 | 8.04 | 51.13 |
| Ovarian cancer | 12.97 | 16.93 | 22.23 | 26.74 | 28.51 | 29.29 | 31.46 | 142.56 |
| Cervical cancer | 52.06 | 58.81 | 65.94 | 69.84 | 77.40 | 75.20 | 78.38 | 50.56 |
| Breast cancer | 108.15 | 121.99 | 143.76 | 166.31 | 190.33 | 194.94 | 213.21 | 97.14 |
| Oesophageal cancer | 2.60 | 2.78 | 2.84 | 2.95 | 2.93 | 2.91 | 3.04 | 16.92 |
| Tracheal, bronchus and lung cancer | 8.86 | 9.38 | 9.94 | 10.86 | 11.66 | 11.96 | 12.65 | 42.78 |
| Prostate cancer | 32.60 | 35.03 | 37.89 | 41.34 | 44.19 | 44.31 | 50.96 | 56.32 |
| Thyroid cancer | 15.34 | 18.92 | 24.24 | 26.88 | 27.83 | 26.29 | 29.79 | 94.20 |
| Leukaemia | 34.30 | 33.10 | 32.41 | 33.28 | 34.75 | 34.13 | 34.50 | 0.58 |
| Multiple myeloma | 2.31 | 2.54 | 2.72 | 2.89 | 2.96 | 2.95 | 3.21 | 38.96 |
| **Cardiovascular diseases (%)** |  |  |  |  |  |  |  |  |
| Stroke | 1.59 | 1.68 | 1.80 | 1.90 | 2.00 | 1.98 | 2.03 | 27.38 |
| Ischemic heart disease | 1.86 | 2.04 | 2.23 | 2.47 | 2.61 | 2.63 | 2.66 | 43.10 |
| **Chronic respiratory diseases (%)** |  |  |  |  |  |  |  |  |
| Chronic obstructive pulmonary disease | 1.33 | 1.37 | 1.40 | 1.42 | 1.34 | 1.27 | 1.29 | -3.42 |
| Asthma | 3.40 | 3.18 | 3.01 | 2.90 | 2.73 | 2.64 | 2.90 | -14.69 |
| **Digestive diseases (%)** |  |  |  |  |  |  |  |  |
| Gallbladder and biliary diseases | 0.95 | 0.97 | 1.02 | 1.10 | 1.13 | 1.14 | 1.15 | 21.85 |
| Upper digestive system diseases | 5.20 | 5.55 | 5.87 | 6.18 | 6.33 | 6.28 | 6.32 | 21.45 |
| **Neurological disorders (%)** |  |  |  |  |  |  |  |  |
| Alzheimer's disease and other dementias | 0.34 | 0.37 | 0.41 | 0.47 | 0.52 | 0.53 | 0.52 | 51.71 |
| Parkinson's disease | 0.09 | 0.10 | 0.11 | 0.12 | 0.13 | 0.13 | 0.13 | 39.88 |
| **Mental disorders (%)** |  |  |  |  |  |  |  |  |
| Depressive disorders | 2.89 | 3.02 | 3.09 | 3.16 | 3.14 | 3.11 | 3.15 | 8.83 |
| Anxiety disorder | 4.07 | 4.19 | 4.27 | 4.24 | 4.27 | 4.33 | 4.38 | 7.48 |
| **Substance use disorders (%)** |  |  |  |  |  |  |  |  |
| Alcohol use disorders | 0.63 | 0.67 | 0.71 | 0.74 | 0.74 | 0.71 | 0.71 | 13.27 |
| Drug use disorders | 0.97 | 1.14 | 1.20 | 1.13 | 1.03 | 0.97 | 1.00 | 3.90 |
| **Diabetes and kidney diseases (%)** |  |  |  |  |  |  |  |  |
| Diabetes mellitus type 2 | 7.10 | 8.66 | 10.74 | 13.15 | 14.68 | 15.84 | 17.12 | 140.93 |
| Chronic kidney disease | 11.63 | 12.91 | 14.39 | 15.81 | 16.74 | 16.80 | 17.27 | 48.47 |
| **Musculoskeletal disorders (%)** |  |  |  |  |  |  |  |  |
| Osteoarthritis | 3.97 | 4.35 | 4.75 | 5.37 | 5.73 | 5.91 | 6.10 | 53.58 |
| **Japan** |  |  |  |  |  |  |  |  |
| **Neoplasms/Cancer (per 100,000 persons)** |  |  |  |  |  |  |  |  |
| Lip and oral cavity cancer | 9.86 | 12.05 | 14.21 | 16.44 | 19.00 | 19.25 | 18.98 | 92.49 |
| Colon and rectum cancer | 390.27 | 497.81 | 564.38 | 649.12 | 729.97 | 809.00 | 845.91 | 116.75 |
| Stomach cancer | 364.06 | 363.77 | 346.01 | 346.15 | 340.06 | 319.74 | 314.95 | -13.49 |
| Pancreatic cancer | 17.75 | 20.70 | 23.95 | 30.75 | 38.42 | 44.16 | 46.65 | 162.82 |
| Non-Hodgkin's lymphoma | 105.31 | 145.01 | 193.84 | 241.14 | 286.55 | 330.53 | 349.91 | 232.27 |
| Gallbladder and biliary tract cancer | 18.28 | 20.11 | 21.54 | 24.47 | 27.36 | 28.74 | 30.01 | 64.17 |
| Kidney cancer | 20.53 | 26.93 | 33.39 | 41.80 | 46.90 | 50.10 | 50.78 | 147.35 |
| Ovarian cancer | 23.38 | 27.94 | 30.86 | 33.65 | 35.06 | 34.84 | 35.15 | 50.34 |
| Cervical cancer | 49.97 | 45.48 | 46.52 | 52.51 | 59.63 | 60.92 | 58.33 | 16.73 |
| Breast cancer | 361.79 | 412.30 | 486.59 | 580.09 | 694.25 | 757.18 | 774.60 | 114.10 |
| Oesophageal cancer | 31.82 | 40.79 | 51.52 | 64.28 | 69.95 | 70.05 | 71.02 | 123.19 |
| Tracheal, bronchus and lung cancer | 84.24 | 103.02 | 125.05 | 167.24 | 208.75 | 223.35 | 224.42 | 166.41 |
| Prostate cancer | 92.35 | 118.77 | 175.67 | 282.47 | 355.05 | 387.68 | 407.89 | 341.68 |
| Thyroid cancer | 42.94 | 49.46 | 55.51 | 65.10 | 70.89 | 67.96 | 66.39 | 54.61 |
| Leukaemia | 74.51 | 93.25 | 116.09 | 136.78 | 145.57 | 148.73 | 148.77 | 99.66 |
| Multiple myeloma | 10.98 | 13.10 | 16.16 | 21.05 | 25.40 | 26.60 | 27.89 | 154.01 |
| **Cardiovascular diseases (%)** |  |  |  |  |  |  |  |  |
| Stroke | 2.15 | 2.35 | 2.67 | 2.82 | 3.09 | 3.35 | 3.53 | 64.46 |
| Ischemic heart disease | 1.99 | 2.17 | 2.35 | 2.50 | 2.69 | 3.00 | 3.26 | 63.90 |
| **Chronic respiratory diseases (%)** |  |  |  |  |  |  |  |  |
| Chronic obstructive pulmonary disease | 2.75 | 3.05 | 3.23 | 3.43 | 3.51 | 3.92 | 4.20 | 52.61 |
| Asthma | 9.44 | 8.93 | 6.96 | 5.06 | 4.25 | 3.76 | 4.00 | -57.67 |
| **Digestive diseases (%)** |  |  |  |  |  |  |  |  |
| Gallbladder and biliary diseases | 5.19 | 5.29 | 5.44 | 5.60 | 5.85 | 6.16 | 6.63 | 27.83 |
| Upper digestive system diseases | 8.06 | 8.33 | 8.43 | 8.74 | 9.22 | 9.38 | 9.62 | 19.30 |
| **Neurological disorders (%)** |  |  |  |  |  |  |  |  |
| Alzheimer's disease and other dementias | 0.90 | 1.10 | 1.43 | 1.83 | 2.27 | 3.01 | 3.47 | 284.99 |
| Parkinson's disease | 0.10 | 0.12 | 0.13 | 0.16 | 0.19 | 0.21 | 0.27 | 173.81 |
| **Mental disorders (%)** |  |  |  |  |  |  |  |  |
| Depressive disorders | 2.54 | 2.44 | 2.77 | 2.88 | 2.74 | 2.66 | 2.72 | 7.07 |
| Anxiety disorder | 2.98 | 2.96 | 2.96 | 2.92 | 2.71 | 2.53 | 2.53 | -15.05 |
| **Substance use disorders (%)** |  |  |  |  |  |  |  |  |
| Alcohol use disorders | 0.72 | 0.71 | 0.69 | 0.67 | 0.65 | 0.66 | 0.68 | -5.42 |
| Drug use disorders | 0.76 | 0.76 | 0.74 | 0.70 | 0.66 | 0.62 | 0.62 | -18.92 |
| **Diabetes and kidney diseases (%)** |  |  |  |  |  |  |  |  |
| Diabetes mellitus type 2 | 5.94 | 6.68 | 7.46 | 7.15 | 7.71 | 8.80 | 10.18 | 71.38 |
| Chronic kidney disease | 17.82 | 19.40 | 21.01 | 23.51 | 25.83 | 27.95 | 30.42 | 70.73 |
| **Musculoskeletal disorders (%)** |  |  |  |  |  |  |  |  |
| Osteoarthritis | 12.10 | 13.58 | 15.11 | 16.74 | 19.33 | 20.54 | 20.25 | 67.36 |
| **Singapore** |  |  |  |  |  |  |  |  |
| **Neoplasms/Cancer (per 100,000 persons)** |  |  |  |  |  |  |  |  |
| Lip and oral cavity cancer | 9.45 | 9.68 | 9.43 | 11.10 | 11.60 | 11.78 | 12.34 | 30.58 |
| Colon and rectum cancer | 212.21 | 260.42 | 284.41 | 342.06 | 415.20 | 488.82 | 551.29 | 159.79 |
| Stomach cancer | 68.17 | 71.56 | 68.16 | 72.38 | 82.23 | 85.69 | 89.29 | 30.98 |
| Pancreatic cancer | 5.42 | 6.16 | 6.93 | 9.12 | 11.76 | 14.60 | 16.31 | 200.92 |
| Non-Hodgkin's lymphoma | 45.32 | 70.05 | 82.95 | 105.15 | 139.31 | 169.50 | 185.79 | 309.95 |
| Gallbladder and biliary tract cancer | 2.07 | 2.52 | 2.71 | 3.26 | 3.46 | 3.73 | 4.14 | 100.00 |
| Kidney cancer | 12.25 | 16.74 | 21.03 | 28.88 | 38.88 | 46.63 | 49.76 | 306.20 |
| Ovarian cancer | 19.13 | 22.12 | 24.38 | 31.42 | 33.19 | 34.20 | 36.31 | 89.81 |
| Cervical cancer | 72.79 | 69.49 | 58.22 | 53.33 | 50.16 | 51.21 | 51.17 | -29.70 |
| Breast cancer | 291.44 | 343.53 | 400.75 | 509.47 | 628.00 | 713.79 | 756.24 | 159.48 |
| Oesophageal cancer | 12.98 | 12.73 | 12.23 | 13.20 | 16.55 | 19.37 | 22.27 | 71.57 |
| Tracheal, bronchus and lung cancer | 54.37 | 61.23 | 65.90 | 81.82 | 102.71 | 122.27 | 137.63 | 153.14 |
| Prostate cancer | 41.21 | 57.47 | 76.71 | 108.42 | 145.86 | 195.14 | 236.41 | 473.67 |
| Thyroid cancer | 35.33 | 39.66 | 47.64 | 60.89 | 65.08 | 64.18 | 67.08 | 89.87 |
| Leukaemia | 26.51 | 31.52 | 35.97 | 50.80 | 76.19 | 93.28 | 99.13 | 273.93 |
| Multiple myeloma | 4.43 | 5.17 | 5.76 | 7.44 | 9.35 | 11.72 | 13.62 | 207.45 |
| **Cardiovascular diseases (%)** |  |  |  |  |  |  |  |  |
| Stroke | 1.93 | 1.91 | 1.84 | 1.88 | 1.91 | 2.11 | 2.27 | 17.73 |
| Ischemic heart disease | 1.59 | 1.65 | 1.70 | 1.95 | 2.22 | 2.65 | 2.94 | 85.07 |
| **Chronic respiratory diseases (%)** |  |  |  |  |  |  |  |  |
| Chronic obstructive pulmonary disease | 2.65 | 2.92 | 2.86 | 2.86 | 2.68 | 3.05 | 3.10 | 16.84 |
| Asthma | 6.76 | 6.26 | 5.03 | 3.82 | 3.37 | 3.51 | 3.53 | -47.81 |
| **Digestive diseases (%)** |  |  |  |  |  |  |  |  |
| Gallbladder and biliary diseases | 4.48 | 4.62 | 4.82 | 5.11 | 5.37 | 5.78 | 6.10 | 36.13 |
| Upper digestive system diseases | 9.02 | 9.54 | 10.22 | 10.98 | 11.48 | 12.02 | 12.40 | 37.53 |
| **Neurological disorders (%)** |  |  |  |  |  |  |  |  |
| Alzheimer's disease and other dementias | 0.40 | 0.46 | 0.51 | 0.65 | 0.76 | 0.96 | 1.25 | 211.63 |
| Parkinson's disease | 0.06 | 0.07 | 0.08 | 0.09 | 0.11 | 0.14 | 0.16 | 170.05 |
| **Mental disorders (%)** |  |  |  |  |  |  |  |  |
| Depressive disorders | 3.54 | 3.59 | 3.50 | 2.78 | 2.53 | 2.43 | 2.57 | -27.52 |
| Anxiety disorder | 3.24 | 3.26 | 3.26 | 3.24 | 3.11 | 3.02 | 2.99 | -7.74 |
| **Substance use disorders (%)** |  |  |  |  |  |  |  |  |
| Alcohol use disorders | 0.87 | 0.90 | 0.94 | 0.87 | 0.87 | 0.82 | 0.86 | -1.09 |
| Drug use disorders | 1.02 | 1.04 | 1.02 | 0.89 | 0.77 | 0.62 | 0.54 | -46.57 |
| **Diabetes and kidney diseases (%)** |  |  |  |  |  |  |  |  |
| Diabetes mellitus type 2 | 7.57 | 9.07 | 9.94 | 11.50 | 13.10 | 15.81 | 15.63 | 106.41 |
| Chronic kidney disease | 14.90 | 16.13 | 17.87 | 19.99 | 21.96 | 24.90 | 26.91 | 80.59 |
| **Musculoskeletal disorders (%)** |  |  |  |  |  |  |  |  |
| Osteoarthritis | 8.68 | 9.55 | 10.58 | 12.71 | 14.96 | 17.94 | 19.73 | 127.44 |
| **Kiribati** |  |  |  |  |  |  |  |  |
| **Neoplasms/Cancer (per 100,000 persons)** |  |  |  |  |  |  |  |  |
| Lip and oral cavity cancer | 35.88 | 34.03 | 38.02 | 41.34 | 50.77 | 54.27 | 60.56 | 68.78 |
| Colon and rectum cancer | 22.93 | 23.63 | 26.07 | 26.65 | 29.41 | 31.00 | 35.24 | 53.69 |
| Stomach cancer | 20.89 | 21.21 | 22.99 | 22.66 | 24.75 | 25.48 | 26.22 | 25.51 |
| Pancreatic cancer | 1.30 | 1.51 | 1.77 | 1.98 | 1.92 | 2.01 | 2.38 | 83.08 |
| Non-Hodgkin's lymphoma | 0.00 | 0.00 | 0.03 | 0.03 | 0.04 | 0.04 | 0.04 | N.A |
| Gallbladder and biliary tract cancer | 1.05 | 1.03 | 1.09 | 1.10 | 1.26 | 1.33 | 1.43 | 36.19 |
| Kidney cancer | 8.48 | 9.40 | 10.89 | 12.54 | 12.87 | 12.96 | 15.48 | 82.55 |
| Ovarian cancer | 3.56 | 4.50 | 5.83 | 6.99 | 7.72 | 8.38 | 9.40 | 164.04 |
| Cervical cancer | 219.04 | 228.29 | 248.69 | 243.81 | 280.27 | 283.33 | 304.39 | 38.97 |
| Breast cancer | 109.97 | 114.75 | 133.87 | 143.87 | 167.64 | 179.78 | 202.61 | 84.24 |
| Oesophageal cancer | 8.74 | 9.12 | 10.10 | 10.79 | 11.28 | 11.69 | 12.59 | 44.05 |
| Tracheal, bronchus and lung cancer | 13.40 | 13.49 | 14.55 | 14.91 | 16.78 | 18.10 | 19.72 | 47.16 |
| Prostate cancer | 13.05 | 12.57 | 12.61 | 12.51 | 15.08 | 16.23 | 19.19 | 47.05 |
| Thyroid cancer | 2.11 | 2.41 | 2.93 | 3.07 | 3.39 | 3.37 | 3.89 | 84.36 |
| Leukaemia | 45.55 | 43.28 | 41.44 | 38.94 | 39.29 | 37.90 | 38.62 | -15.21 |
| Multiple myeloma | 1.57 | 1.60 | 1.72 | 1.76 | 1.92 | 2.04 | 2.24 | 42.68 |
| **Cardiovascular diseases (%)** |  |  |  |  |  |  |  |  |
| Stroke | 1.91 | 1.97 | 2.05 | 2.13 | 2.25 | 2.41 | 2.45 | 28.36 |
| Ischemic heart disease | 1.35 | 1.40 | 1.53 | 1.62 | 1.82 | 1.98 | 2.12 | 56.82 |
| **Chronic respiratory diseases (%)** |  |  |  |  |  |  |  |  |
| Chronic obstructive pulmonary disease | 0.74 | 0.74 | 0.79 | 0.82 | 0.87 | 0.90 | 0.95 | 28.55 |
| Asthma | 6.50 | 6.32 | 6.13 | 5.76 | 5.42 | 5.05 | 5.04 | -22.44 |
| **Digestive diseases (%)** |  |  |  |  |  |  |  |  |
| Gallbladder and biliary diseases | 1.29 | 1.32 | 1.40 | 1.43 | 1.54 | 1.64 | 1.68 | 29.92 |
| Upper digestive system diseases | 5.41 | 5.54 | 5.87 | 6.15 | 6.61 | 6.84 | 6.97 | 28.85 |
| **Neurological disorders (%)** |  |  |  |  |  |  |  |  |
| Alzheimer's disease and other dementias | 0.20 | 0.20 | 0.22 | 0.22 | 0.24 | 0.26 | 0.28 | 44.72 |
| Parkinson's disease | 0.06 | 0.06 | 0.06 | 0.06 | 0.07 | 0.08 | 0.08 | 37.47 |
| **Mental disorders (%)** |  |  |  |  |  |  |  |  |
| Depressive disorders | 2.99 | 3.01 | 3.13 | 3.28 | 3.46 | 3.51 | 3.53 | 18.14 |
| Anxiety disorder | 3.94 | 4.07 | 4.29 | 4.34 | 4.41 | 4.44 | 4.44 | 12.56 |
| **Substance use disorders (%)** |  |  |  |  |  |  |  |  |
| Alcohol use disorders | 0.60 | 0.62 | 0.65 | 0.68 | 0.73 | 0.73 | 0.75 | 23.60 |
| Drug use disorders | 0.75 | 0.74 | 0.80 | 0.87 | 0.87 | 0.78 | 0.72 | -4.17 |
| **Diabetes and kidney diseases (%)** |  |  |  |  |  |  |  |  |
| Diabetes mellitus type 2 | 7.77 | 9.14 | 11.41 | 13.55 | 15.96 | 18.28 | 20.12 | 158.97 |
| Chronic kidney disease | 10.66 | 11.22 | 12.31 | 13.09 | 14.47 | 15.52 | 16.46 | 54.33 |
| **Musculoskeletal disorders (%)** |  |  |  |  |  |  |  |  |
| Osteoarthritis | 3.17 | 3.32 | 3.71 | 4.10 | 4.53 | 4.99 | 5.38 | 69.98 |
| **Solomon Islands** |  |  |  |  |  |  |  |  |
| **Neoplasms/Cancer (per 100,000 persons)** |  |  |  |  |  |  |  |  |
| Lip and oral cavity cancer | 6.68 | 7.81 | 9.27 | 10.41 | 12.12 | 15.60 | 17.04 | 155.09 |
| Colon and rectum cancer | 17.49 | 21.52 | 26.31 | 28.40 | 32.59 | 42.70 | 47.49 | 171.53 |
| Stomach cancer | 23.95 | 27.21 | 31.23 | 35.27 | 38.82 | 44.14 | 44.88 | 87.39 |
| Pancreatic cancer | 0.94 | 1.24 | 1.63 | 2.07 | 2.16 | 2.71 | 3.00 | 219.15 |
| Non-Hodgkin's lymphoma | 7.79 | 9.56 | 9.74 | 5.44 | 4.37 | 6.83 | 12.81 | 64.44 |
| Gallbladder and biliary tract cancer | 0.91 | 1.06 | 1.23 | 1.35 | 1.47 | 1.79 | 1.86 | 104.40 |
| Kidney cancer | 2.66 | 3.22 | 4.18 | 4.67 | 4.99 | 6.57 | 7.42 | 178.95 |
| Ovarian cancer | 3.59 | 4.95 | 7.12 | 9.55 | 11.54 | 15.32 | 17.05 | 374.93 |
| Cervical cancer | 86.08 | 100.68 | 124.04 | 143.45 | 172.73 | 211.74 | 220.61 | 156.28 |
| Breast cancer | 52.42 | 60.52 | 87.69 | 183.44 | 256.02 | 355.20 | 629.03 | 1099.98 |
| Oesophageal cancer | 3.18 | 3.69 | 4.28 | 4.88 | 5.04 | 5.90 | 6.12 | 92.45 |
| Tracheal, bronchus and lung cancer | 15.72 | 18.31 | 21.22 | 23.14 | 25.70 | 31.71 | 33.57 | 113.55 |
| Prostate cancer | 19.85 | 24.16 | 30.15 | 32.22 | 36.03 | 47.60 | 53.17 | 167.86 |
| Thyroid cancer | 8.14 | 10.91 | 15.44 | 18.31 | 21.36 | 28.30 | 31.50 | 286.98 |
| Leukaemia | 49.10 | 48.58 | 52.25 | 53.33 | 54.18 | 56.38 | 55.57 | 13.18 |
| Multiple myeloma | 2.13 | 2.49 | 2.92 | 3.27 | 3.49 | 4.37 | 4.66 | 118.78 |
| **Cardiovascular diseases (%)** |  |  |  |  |  |  |  |  |
| Stroke | 1.17 | 1.32 | 1.46 | 1.58 | 1.75 | 1.95 | 2.02 | 72.90 |
| Ischemic heart disease | 1.02 | 1.21 | 1.36 | 1.51 | 1.68 | 1.92 | 2.00 | 96.04 |
| **Chronic respiratory diseases (%)** |  |  |  |  |  |  |  |  |
| Chronic obstructive pulmonary disease | 0.89 | 1.00 | 1.08 | 1.11 | 1.13 | 1.25 | 1.30 | 45.30 |
| Asthma | 3.64 | 3.28 | 3.26 | 3.08 | 2.98 | 3.03 | 3.44 | -5.56 |
| **Digestive diseases (%)** |  |  |  |  |  |  |  |  |
| Gallbladder and biliary diseases | 0.92 | 1.02 | 1.13 | 1.24 | 1.34 | 1.45 | 1.48 | 61.00 |
| Upper digestive system diseases | 4.56 | 5.18 | 5.78 | 6.30 | 6.70 | 6.99 | 7.20 | 57.92 |
| **Neurological disorders (%)** |  |  |  |  |  |  |  |  |
| Alzheimer's disease and other dementias | 0.13 | 0.15 | 0.18 | 0.20 | 0.23 | 0.26 | 0.27 | 107.21 |
| Parkinson's disease | 0.05 | 0.06 | 0.07 | 0.07 | 0.08 | 0.09 | 0.09 | 93.21 |
| **Mental disorders (%)** |  |  |  |  |  |  |  |  |
| Depressive disorders | 2.68 | 3.05 | 3.34 | 3.52 | 3.59 | 3.69 | 3.78 | 40.93 |
| Anxiety disorder | 3.92 | 4.26 | 4.37 | 4.28 | 4.34 | 4.42 | 4.48 | 14.30 |
| **Substance use disorders (%)** |  |  |  |  |  |  |  |  |
| Alcohol use disorders | 0.60 | 0.70 | 0.79 | 0.88 | 0.93 | 0.94 | 0.96 | 58.49 |
| Drug use disorders | 0.81 | 0.93 | 1.05 | 0.98 | 0.84 | 0.73 | 0.73 | -10.82 |
| **Diabetes and kidney diseases (%)** |  |  |  |  |  |  |  |  |
| Diabetes mellitus type 2 | 4.51 | 5.75 | 7.36 | 9.46 | 11.99 | 15.32 | 17.32 | 284.05 |
| Chronic kidney disease | 8.84 | 10.21 | 11.68 | 13.06 | 14.58 | 16.01 | 16.75 | 89.57 |
| **Musculoskeletal disorders (%)** |  |  |  |  |  |  |  |  |
| Osteoarthritis | 2.35 | 2.77 | 3.17 | 3.66 | 4.10 | 4.83 | 5.20 | 121.05 |
| **Tokelau (New Zealand)** |  |  |  |  |  |  |  |  |
| **Neoplasms/Cancer (per 100,000 persons)** |  |  |  |  |  |  |  |  |
| Lip and oral cavity cancer | 9.41 | 9.43 | 9.53 | 10.95 | 12.14 | 12.36 | 13.52 | 43.68 |
| Colon and rectum cancer | 38.03 | 38.80 | 40.36 | 49.04 | 54.74 | 57.96 | 65.06 | 71.08 |
| Stomach cancer | 21.57 | 20.82 | 19.20 | 20.64 | 20.05 | 18.59 | 18.65 | -13.54 |
| Pancreatic cancer | 2.66 | 2.91 | 3.14 | 4.14 | 4.31 | 4.46 | 5.02 | 88.72 |
| Non-Hodgkin's lymphoma | 0.00 | 0.00 | 0.00 | 0.00 | 0.27 | 1.10 | 3.45 | N.A |
| Gallbladder and biliary tract cancer | 1.60 | 1.53 | 1.45 | 1.55 | 1.49 | 1.37 | 1.40 | -12.50 |
| Kidney cancer | 4.64 | 5.00 | 5.66 | 6.80 | 7.37 | 8.22 | 9.42 | 103.02 |
| Ovarian cancer | 6.94 | 8.07 | 10.14 | 12.74 | 13.80 | 14.71 | 16.30 | 134.87 |
| Cervical cancer | 91.79 | 92.00 | 93.93 | 96.46 | 100.46 | 97.67 | 104.62 | 13.98 |
| Breast cancer | 176.87 | 182.41 | 207.43 | 249.75 | 279.09 | 287.99 | 312.05 | 76.43 |
| Oesophageal cancer | 3.92 | 3.75 | 3.38 | 3.97 | 3.76 | 3.75 | 3.79 | -3.32 |
| Tracheal, bronchus and lung cancer | 23.45 | 23.09 | 22.04 | 26.36 | 27.49 | 27.92 | 29.67 | 26.52 |
| Prostate cancer | 47.20 | 51.17 | 51.35 | 67.58 | 75.82 | 81.66 | 95.66 | 102.67 |
| Thyroid cancer | 10.84 | 12.32 | 14.60 | 17.88 | 20.53 | 21.42 | 24.67 | 127.58 |
| Leukaemia | 40.72 | 38.87 | 37.92 | 38.05 | 34.77 | 32.89 | 32.10 | -21.17 |
| Multiple myeloma | 3.70 | 3.49 | 3.34 | 3.81 | 3.95 | 4.09 | 4.34 | 17.30 |
| **Cardiovascular diseases (%)** |  |  |  |  |  |  |  |  |
| Stroke | 1.86 | 1.82 | 1.80 | 1.97 | 1.95 | 1.86 | 1.93 | 4.05 |
| Ischemic heart disease | 2.71 | 2.80 | 2.71 | 3.17 | 3.11 | 2.96 | 3.02 | 11.57 |
| **Chronic respiratory diseases (%)** |  |  |  |  |  |  |  |  |
| Chronic obstructive pulmonary disease | 3.52 | 3.57 | 3.50 | 4.01 | 3.94 | 3.75 | 3.78 | 7.47 |
| Asthma | 3.48 | 2.62 | 2.55 | 2.62 | 2.79 | 2.77 | 3.38 | -2.95 |
| **Digestive diseases (%)** |  |  |  |  |  |  |  |  |
| Gallbladder and biliary diseases | 1.15 | 1.09 | 1.09 | 1.14 | 1.11 | 1.07 | 1.10 | -4.64 |
| Upper digestive system diseases | 5.79 | 5.78 | 5.80 | 6.18 | 6.26 | 6.24 | 6.37 | 9.99 |
| **Neurological disorders (%)** |  |  |  |  |  |  |  |  |
| Alzheimer's disease and other dementias | 0.62 | 0.67 | 0.68 | 0.82 | 0.78 | 0.67 | 0.63 | 1.10 |
| Parkinson's disease | 0.15 | 0.15 | 0.15 | 0.17 | 0.17 | 0.15 | 0.16 | 4.28 |
| **Mental disorders (%)** |  |  |  |  |  |  |  |  |
| Depressive disorders | 3.19 | 3.20 | 3.23 | 3.38 | 3.47 | 3.45 | 3.49 | 9.33 |
| Anxiety disorder | 4.05 | 4.06 | 4.16 | 4.14 | 4.26 | 4.26 | 4.27 | 5.46 |
| **Substance use disorders (%)** |  |  |  |  |  |  |  |  |
| Alcohol use disorders | 0.67 | 0.69 | 0.71 | 0.76 | 0.79 | 0.80 | 0.81 | 22.37 |
| Drug use disorders | 0.69 | 0.70 | 0.72 | 0.71 | 0.74 | 0.72 | 0.72 | 3.56 |
| **Diabetes and kidney diseases (%)** |  |  |  |  |  |  |  |  |
| Diabetes mellitus type 2 | 8.72 | 9.69 | 10.89 | 13.62 | 14.91 | 16.06 | 17.75 | 103.43 |
| Chronic kidney disease | 13.33 | 13.91 | 14.42 | 16.22 | 16.79 | 16.96 | 17.73 | 32.99 |
| **Musculoskeletal disorders (%)** |  |  |  |  |  |  |  |  |
| Osteoarthritis | 5.36 | 5.35 | 5.39 | 6.43 | 6.46 | 6.45 | 6.70 | 25.07 |
| **Tonga** |  |  |  |  |  |  |  |  |
| **Neoplasms/Cancer (per 100,000 persons)** |  |  |  |  |  |  |  |  |
| Lip and oral cavity cancer | 6.48 | 7.55 | 9.52 | 10.70 | 12.55 | 15.28 | 17.58 | 171.30 |
| Colon and rectum cancer | 19.24 | 23.62 | 30.87 | 34.03 | 39.61 | 48.85 | 58.32 | 203.12 |
| Stomach cancer | 19.91 | 22.70 | 29.39 | 29.91 | 33.44 | 37.29 | 40.29 | 102.36 |
| Pancreatic cancer | 2.25 | 3.23 | 4.77 | 5.53 | 5.69 | 6.86 | 8.12 | 260.89 |
| Non-Hodgkin's lymphoma | 5.97 | 7.25 | 8.73 | 7.24 | 8.62 | 11.09 | 11.21 | 87.77 |
| Gallbladder and biliary tract cancer | 0.79 | 0.93 | 1.18 | 1.24 | 1.41 | 1.65 | 1.85 | 134.18 |
| Kidney cancer | 3.98 | 5.24 | 7.54 | 8.85 | 9.06 | 10.57 | 12.75 | 220.35 |
| Ovarian cancer | 7.00 | 9.24 | 12.50 | 15.87 | 17.65 | 21.10 | 24.49 | 249.86 |
| Cervical cancer | 80.52 | 89.88 | 101.37 | 101.44 | 117.59 | 134.83 | 146.89 | 82.43 |
| Breast cancer | 164.67 | 201.52 | 250.94 | 282.70 | 333.14 | 410.16 | 479.38 | 191.12 |
| Oesophageal cancer | 2.82 | 3.52 | 4.76 | 5.02 | 5.27 | 6.02 | 6.79 | 140.78 |
| Tracheal, bronchus and lung cancer | 22.06 | 25.84 | 33.43 | 35.00 | 40.06 | 47.37 | 53.46 | 142.34 |
| Prostate cancer | 54.69 | 71.09 | 100.38 | 109.29 | 128.37 | 156.22 | 185.27 | 238.76 |
| Thyroid cancer | 7.26 | 9.46 | 13.23 | 15.48 | 17.01 | 20.15 | 23.18 | 219.28 |
| Leukaemia | 20.21 | 19.53 | 22.78 | 23.16 | 24.82 | 26.18 | 28.02 | 38.64 |
| Multiple myeloma | 3.68 | 4.47 | 5.73 | 6.24 | 6.82 | 8.01 | 9.21 | 150.27 |
| **Cardiovascular diseases (%)** |  |  |  |  |  |  |  |  |
| Stroke | 1.36 | 1.50 | 1.70 | 1.86 | 2.07 | 2.34 | 2.65 | 95.39 |
| Ischemic heart disease | 2.10 | 2.56 | 2.97 | 3.46 | 3.98 | 4.68 | 5.21 | 148.21 |
| **Chronic respiratory diseases (%)** |  |  |  |  |  |  |  |  |
| Chronic obstructive pulmonary disease | 1.18 | 1.33 | 1.47 | 1.64 | 1.71 | 1.95 | 2.11 | 79.31 |
| Asthma | 4.39 | 3.89 | 3.80 | 3.55 | 3.25 | 3.28 | 3.66 | -16.48 |
| **Digestive diseases (%)** |  |  |  |  |  |  |  |  |
| Gallbladder and biliary diseases | 1.12 | 1.21 | 1.33 | 1.47 | 1.58 | 1.78 | 1.90 | 70.01 |
| Upper digestive system diseases | 5.62 | 6.29 | 7.05 | 7.80 | 8.25 | 8.69 | 8.95 | 59.25 |
| **Neurological disorders (%)** |  |  |  |  |  |  |  |  |
| Alzheimer's disease and other dementias | 0.38 | 0.47 | 0.57 | 0.69 | 0.82 | 1.02 | 1.15 | 205.44 |
| Parkinson's disease | 0.10 | 0.11 | 0.13 | 0.15 | 0.18 | 0.21 | 0.23 | 145.72 |
| **Mental disorders (%)** |  |  |  |  |  |  |  |  |
| Depressive disorders | 2.77 | 3.07 | 3.37 | 3.59 | 3.67 | 3.82 | 3.95 | 42.56 |
| Anxiety disorder | 4.29 | 4.56 | 4.63 | 4.60 | 4.64 | 4.72 | 4.74 | 10.52 |
| **Substance use disorders (%)** |  |  |  |  |  |  |  |  |
| Alcohol use disorders | 0.60 | 0.69 | 0.80 | 0.90 | 0.94 | 0.93 | 0.90 | 49.20 |
| Drug use disorders | 0.83 | 0.99 | 1.09 | 0.96 | 0.78 | 0.62 | 0.55 | -33.90 |
| **Diabetes and kidney diseases (%)** |  |  |  |  |  |  |  |  |
| Diabetes mellitus type 2 | 7.81 | 9.77 | 12.28 | 15.73 | 18.77 | 23.06 | 26.90 | 244.27 |
| Chronic kidney disease | 12.68 | 14.74 | 17.23 | 20.12 | 22.69 | 25.52 | 27.56 | 117.33 |
| **Musculoskeletal disorders (%)** |  |  |  |  |  |  |  |  |
| Osteoarthritis | 4.45 | 5.33 | 6.27 | 7.47 | 8.28 | 9.82 | 11.29 | 153.85 |
| **Malaysia** |  |  |  |  |  |  |  |  |
| **Neoplasms/Cancer (per 100,000 persons)** |  |  |  |  |  |  |  |  |
| Lip and oral cavity cancer | 14.72 | 16.08 | 17.08 | 18.24 | 21.74 | 28.63 | 38.15 | 159.17 |
| Colon and rectum cancer | 47.03 | 56.74 | 70.28 | 86.08 | 111.52 | 155.04 | 222.22 | 372.51 |
| Stomach cancer | 11.46 | 12.19 | 12.75 | 13.32 | 15.02 | 17.53 | 22.02 | 92.15 |
| Pancreatic cancer | 1.29 | 1.62 | 2.28 | 3.07 | 4.19 | 5.10 | 6.10 | 372.87 |
| Non-Hodgkin's lymphoma | 2.03 | 3.24 | 9.07 | 16.41 | 27.64 | 44.39 | 69.17 | 3307.39 |
| Gallbladder and biliary tract cancer | 1.16 | 1.24 | 1.33 | 1.48 | 1.83 | 2.30 | 2.89 | 149.14 |
| Kidney cancer | 5.53 | 6.77 | 9.22 | 12.19 | 16.78 | 22.95 | 30.44 | 450.45 |
| Ovarian cancer | 9.18 | 11.61 | 16.25 | 22.01 | 26.72 | 30.42 | 32.58 | 254.90 |
| Cervical cancer | 46.63 | 49.70 | 52.09 | 53.21 | 58.24 | 71.91 | 85.66 | 83.70 |
| Breast cancer | 123.62 | 143.93 | 176.79 | 225.49 | 290.90 | 382.71 | 478.40 | 286.99 |
| Oesophageal cancer | 3.46 | 3.73 | 4.10 | 4.47 | 5.18 | 6.52 | 8.63 | 149.42 |
| Tracheal, bronchus and lung cancer | 12.49 | 14.81 | 18.44 | 21.96 | 27.12 | 31.29 | 36.90 | 195.44 |
| Prostate cancer | 16.90 | 20.85 | 27.53 | 38.63 | 53.21 | 77.03 | 106.84 | 532.19 |
| Thyroid cancer | 17.95 | 21.55 | 26.98 | 34.07 | 41.00 | 50.38 | 61.93 | 245.01 |
| Leukaemia | 32.79 | 29.68 | 28.83 | 30.12 | 33.54 | 37.49 | 44.52 | 35.77 |
| Multiple myeloma | 1.87 | 2.13 | 2.59 | 3.24 | 4.19 | 5.24 | 6.39 | 241.71 |
| **Cardiovascular diseases (%)** |  |  |  |  |  |  |  |  |
| Stroke | 1.57 | 1.56 | 1.63 | 1.76 | 1.99 | 2.34 | 2.70 | 71.67 |
| Ischemic heart disease | 1.55 | 1.61 | 1.85 | 2.14 | 2.65 | 3.18 | 3.72 | 139.56 |
| **Chronic respiratory diseases (%)** |  |  |  |  |  |  |  |  |
| Chronic obstructive pulmonary disease | 1.60 | 1.71 | 1.92 | 2.23 | 2.56 | 2.95 | 3.28 | 105.01 |
| Asthma | 3.18 | 3.27 | 3.12 | 2.89 | 2.62 | 2.27 | 2.29 | -27.92 |
| **Digestive diseases (%)** |  |  |  |  |  |  |  |  |
| Gallbladder and biliary diseases | 1.39 | 1.46 | 1.55 | 1.62 | 1.77 | 1.88 | 2.04 | 46.93 |
| Upper digestive system diseases | 5.72 | 6.18 | 6.79 | 7.35 | 7.87 | 8.30 | 8.63 | 50.91 |
| **Neurological disorders (%)** |  |  |  |  |  |  |  |  |
| Alzheimer's disease and other dementias | 0.32 | 0.33 | 0.36 | 0.42 | 0.52 | 0.66 | 0.81 | 156.29 |
| Parkinson's disease | 0.06 | 0.06 | 0.07 | 0.08 | 0.10 | 0.14 | 0.17 | 174.36 |
| **Mental disorders (%)** |  |  |  |  |  |  |  |  |
| Depressive disorders | 3.46 | 3.71 | 3.79 | 4.38 | 4.50 | 4.89 | 5.13 | 48.30 |
| Anxiety disorder | 4.81 | 5.09 | 5.29 | 5.34 | 5.43 | 5.76 | 5.81 | 20.72 |
| **Substance use disorders (%)** |  |  |  |  |  |  |  |  |
| Alcohol use disorders | 0.60 | 0.66 | 0.72 | 0.74 | 0.71 | 0.69 | 0.69 | 15.25 |
| Drug use disorders | 0.65 | 0.71 | 0.73 | 0.75 | 0.70 | 0.62 | 0.56 | -13.81 |
| **Diabetes and kidney diseases (%)** |  |  |  |  |  |  |  |  |
| Diabetes mellitus type 2 | 4.84 | 5.75 | 7.27 | 8.98 | 10.29 | 13.78 | 14.99 | 209.55 |
| Chronic kidney disease | 11.68 | 12.91 | 14.67 | 16.78 | 19.40 | 22.09 | 24.52 | 109.86 |
| **Musculoskeletal disorders (%)** |  |  |  |  |  |  |  |  |
| Osteoarthritis | 3.14 | 3.46 | 4.05 | 4.80 | 5.83 | 7.02 | 8.20 | 160.86 |
| **Tuvalu** |  |  |  |  |  |  |  |  |
| **Neoplasms/Cancer (per 100,000 persons)** |  |  |  |  |  |  |  |  |
| Lip and oral cavity cancer | 10.06 | 11.08 | 11.34 | 12.12 | 13.34 | 14.21 | 15.58 | 54.87 |
| Colon and rectum cancer | 33.13 | 39.37 | 42.51 | 45.98 | 50.05 | 54.31 | 61.20 | 84.73 |
| Stomach cancer | 26.80 | 27.08 | 26.27 | 26.18 | 26.74 | 27.19 | 28.17 | 5.11 |
| Pancreatic cancer | 2.33 | 2.92 | 3.28 | 3.87 | 4.01 | 4.43 | 4.98 | 113.73 |
| Non-Hodgkin's lymphoma | 0.00 | 0.00 | 0.00 | 0.00 | 0.00 | 0.00 | 0.00 | N.A |
| Gallbladder and biliary tract cancer | 1.57 | 1.65 | 1.63 | 1.60 | 1.61 | 1.64 | 1.72 | 9.55 |
| Kidney cancer | 4.82 | 5.77 | 6.36 | 6.73 | 7.09 | 7.73 | 8.93 | 85.27 |
| Ovarian cancer | 8.46 | 10.78 | 13.01 | 15.53 | 16.52 | 17.94 | 19.33 | 128.49 |
| Cervical cancer | 105.18 | 109.11 | 107.19 | 103.34 | 105.56 | 103.60 | 106.05 | 0.83 |
| Breast cancer | 174.40 | 199.47 | 224.07 | 248.31 | 272.08 | 289.78 | 314.35 | 80.25 |
| Oesophageal cancer | 4.58 | 4.81 | 4.66 | 4.65 | 4.62 | 4.79 | 5.09 | 11.14 |
| Tracheal, bronchus and lung cancer | 23.84 | 26.10 | 26.85 | 28.52 | 30.32 | 32.28 | 34.89 | 46.35 |
| Prostate cancer | 40.75 | 52.02 | 57.00 | 60.58 | 67.97 | 76.90 | 91.89 | 125.50 |
| Thyroid cancer | 11.33 | 14.20 | 16.81 | 18.37 | 19.99 | 20.81 | 23.09 | 103.80 |
| Leukaemia | 54.52 | 48.16 | 43.69 | 40.45 | 40.01 | 38.61 | 38.35 | -29.66 |
| Multiple myeloma | 3.67 | 3.95 | 3.91 | 3.97 | 4.11 | 4.39 | 4.79 | 30.52 |
| **Cardiovascular diseases (%)** |  |  |  |  |  |  |  |  |
| Stroke | 1.75 | 1.86 | 1.92 | 2.00 | 2.05 | 2.12 | 2.23 | 27.38 |
| Ischemic heart disease | 2.08 | 2.36 | 2.51 | 2.73 | 2.84 | 2.98 | 3.17 | 52.39 |
| **Chronic respiratory diseases (%)** |  |  |  |  |  |  |  |  |
| Chronic obstructive pulmonary disease | 2.81 | 3.13 | 3.21 | 3.38 | 3.41 | 3.53 | 3.70 | 31.56 |
| Asthma | 3.06 | 2.78 | 2.53 | 2.26 | 2.03 | 1.95 | 3.19 | 4.40 |
| **Digestive diseases (%)** |  |  |  |  |  |  |  |  |
| Gallbladder and biliary diseases | 1.24 | 1.25 | 1.25 | 1.29 | 1.28 | 1.30 | 1.35 | 8.28 |
| Upper digestive system diseases | 6.06 | 6.27 | 6.43 | 6.75 | 6.96 | 7.18 | 7.39 | 21.83 |
| **Neurological disorders (%)** |  |  |  |  |  |  |  |  |
| Alzheimer's disease and other dementias | 0.36 | 0.42 | 0.46 | 0.52 | 0.54 | 0.56 | 0.60 | 63.87 |
| Parkinson's disease | 0.10 | 0.11 | 0.13 | 0.14 | 0.14 | 0.15 | 0.16 | 59.49 |
| **Mental disorders (%)** |  |  |  |  |  |  |  |  |
| Depressive disorders | 3.40 | 3.50 | 3.58 | 3.74 | 3.85 | 3.93 | 3.99 | 17.28 |
| Anxiety disorder | 4.18 | 4.29 | 4.38 | 4.41 | 4.47 | 4.54 | 4.55 | 8.87 |
| **Substance use disorders (%)** |  |  |  |  |  |  |  |  |
| Alcohol use disorders | 0.73 | 0.76 | 0.80 | 0.85 | 0.90 | 0.93 | 0.97 | 33.34 |
| Drug use disorders | 0.73 | 0.73 | 0.76 | 0.80 | 0.80 | 0.77 | 0.75 | 1.83 |
| **Diabetes and kidney diseases (%)** |  |  |  |  |  |  |  |  |
| Diabetes mellitus type 2 | 8.49 | 10.19 | 12.10 | 14.67 | 16.84 | 19.30 | 21.50 | 153.35 |
| Chronic kidney disease | 13.05 | 14.40 | 15.37 | 16.59 | 17.53 | 18.67 | 19.90 | 52.47 |
| **Musculoskeletal disorders (%)** |  |  |  |  |  |  |  |  |
| Osteoarthritis | 4.79 | 5.33 | 5.68 | 6.29 | 6.52 | 6.93 | 7.33 | 53.01 |
| **Marshall Islands** |  |  |  |  |  |  |  |  |
| **Neoplasms/Cancer (per 100,000 persons)** |  |  |  |  |  |  |  |  |
| Lip and oral cavity cancer | 6.43 | 6.71 | 7.15 | 8.94 | 10.95 | 12.85 | 14.65 | 127.84 |
| Colon and rectum cancer | 20.05 | 21.16 | 22.93 | 28.71 | 36.16 | 44.03 | 51.64 | 157.56 |
| Stomach cancer | 18.12 | 17.87 | 19.24 | 22.25 | 25.42 | 27.71 | 30.03 | 65.73 |
| Pancreatic cancer | 1.18 | 1.34 | 1.58 | 2.11 | 2.69 | 3.40 | 4.08 | 245.76 |
| Non-Hodgkin's lymphoma | 0.04 | 0.00 | 0.00 | 0.00 | 0.00 | 0.00 | 0.05 | 25.00 |
| Gallbladder and biliary tract cancer | 0.87 | 0.85 | 0.91 | 1.06 | 1.24 | 1.42 | 1.60 | 83.91 |
| Kidney cancer | 2.74 | 3.13 | 3.36 | 4.15 | 4.87 | 5.94 | 7.12 | 159.85 |
| Ovarian cancer | 4.65 | 6.46 | 8.44 | 11.67 | 14.10 | 17.11 | 19.59 | 321.29 |
| Cervical cancer | 56.60 | 69.30 | 77.30 | 95.07 | 111.25 | 122.13 | 131.77 | 132.81 |
| Breast cancer | 81.53 | 96.75 | 117.71 | 157.17 | 201.44 | 245.21 | 287.39 | 252.50 |
| Oesophageal cancer | 2.60 | 2.53 | 2.80 | 3.32 | 3.87 | 4.46 | 5.00 | 92.31 |
| Tracheal, bronchus and lung cancer | 14.87 | 14.11 | 15.75 | 19.26 | 24.14 | 28.78 | 32.83 | 120.78 |
| Prostate cancer | 24.35 | 19.28 | 20.55 | 26.38 | 34.08 | 45.10 | 56.77 | 133.14 |
| Thyroid cancer | 6.23 | 7.98 | 9.46 | 12.87 | 15.62 | 18.24 | 20.88 | 235.15 |
| Leukaemia | 36.93 | 37.67 | 38.51 | 40.65 | 40.41 | 39.97 | 40.78 | 10.43 |
| Multiple myeloma | 2.05 | 1.99 | 2.15 | 2.64 | 3.20 | 3.80 | 4.35 | 112.20 |
| **Cardiovascular diseases (%)** |  |  |  |  |  |  |  |  |
| Stroke | 1.32 | 1.39 | 1.54 | 1.75 | 2.03 | 2.29 | 2.49 | 88.44 |
| Ischemic heart disease | 1.13 | 1.16 | 1.23 | 1.41 | 1.68 | 2.00 | 2.29 | 101.88 |
| **Chronic respiratory diseases (%)** |  |  |  |  |  |  |  |  |
| Chronic obstructive pulmonary disease | 1.19 | 1.17 | 1.17 | 1.27 | 1.36 | 1.51 | 1.63 | 36.91 |
| Asthma | 3.72 | 3.39 | 3.16 | 3.01 | 2.66 | 2.50 | 2.87 | -22.83 |
| **Digestive diseases (%)** |  |  |  |  |  |  |  |  |
| Gallbladder and biliary diseases | 0.97 | 1.01 | 1.09 | 1.21 | 1.32 | 1.44 | 1.53 | 58.05 |
| Upper digestive system diseases | 4.81 | 5.07 | 5.48 | 6.08 | 6.71 | 7.19 | 7.54 | 56.66 |
| **Neurological disorders (%)** |  |  |  |  |  |  |  |  |
| Alzheimer's disease and other dementias | 0.20 | 0.19 | 0.19 | 0.20 | 0.22 | 0.26 | 0.30 | 53.08 |
| Parkinson's disease | 0.07 | 0.06 | 0.07 | 0.07 | 0.09 | 0.10 | 0.12 | 78.01 |
| **Mental disorders (%)** |  |  |  |  |  |  |  |  |
| Depressive disorders | 2.65 | 2.82 | 3.04 | 3.32 | 3.54 | 3.70 | 3.81 | 43.95 |
| Anxiety disorder | 3.91 | 4.12 | 4.29 | 4.36 | 4.51 | 4.57 | 4.57 | 16.92 |
| **Substance use disorders (%)** |  |  |  |  |  |  |  |  |
| Alcohol use disorders | 0.68 | 0.75 | 0.83 | 0.93 | 1.02 | 1.08 | 1.11 | 63.86 |
| Drug use disorders | 0.81 | 0.87 | 0.94 | 0.98 | 0.95 | 0.88 | 0.81 | 1.10 |
| **Diabetes and kidney diseases (%)** |  |  |  |  |  |  |  |  |
| Diabetes mellitus type 2 | 9.95 | 11.93 | 15.67 | 20.03 | 24.43 | 30.36 | 34.98 | 251.72 |
| Chronic kidney disease | 9.56 | 10.25 | 11.16 | 12.81 | 14.84 | 16.80 | 18.41 | 92.50 |
| **Musculoskeletal disorders (%)** |  |  |  |  |  |  |  |  |
| Osteoarthritis | 2.55 | 2.67 | 3.00 | 3.64 | 4.39 | 5.27 | 6.08 | 138.39 |
| **Vanuatu** |  |  |  |  |  |  |  |  |
| **Neoplasms/Cancer (per 100,000 persons)** |  |  |  |  |  |  |  |  |
| Lip and oral cavity cancer | 5.13 | 6.33 | 6.98 | 7.23 | 8.77 | 10.13 | 11.34 | 121.05 |
| Colon and rectum cancer | 15.16 | 18.59 | 20.80 | 21.76 | 27.18 | 33.15 | 37.00 | 144.06 |
| Stomach cancer | 15.36 | 18.52 | 19.67 | 20.59 | 23.25 | 26.05 | 27.37 | 78.19 |
| Pancreatic cancer | 1.01 | 1.29 | 1.48 | 1.74 | 2.24 | 2.87 | 3.25 | 221.78 |
| Non-Hodgkin's lymphoma | 0.00 | 0.00 | 0.00 | 0.00 | 0.00 | 0.00 | 0.00 | N.A |
| Gallbladder and biliary tract cancer | 0.70 | 0.83 | 0.88 | 0.91 | 1.08 | 1.27 | 1.38 | 97.14 |
| Kidney cancer | 2.40 | 2.77 | 3.13 | 3.16 | 3.93 | 4.67 | 5.29 | 120.42 |
| Ovarian cancer | 2.65 | 3.91 | 4.99 | 5.90 | 7.74 | 9.56 | 10.36 | 290.94 |
| Cervical cancer | 41.02 | 56.06 | 63.27 | 62.01 | 73.37 | 81.12 | 86.39 | 110.60 |
| Breast cancer | 56.08 | 69.13 | 82.48 | 92.71 | 123.52 | 151.81 | 171.17 | 205.22 |
| Oesophageal cancer | 2.47 | 2.93 | 3.06 | 3.26 | 3.78 | 4.44 | 4.80 | 94.33 |
| Tracheal, bronchus and lung cancer | 12.06 | 14.20 | 15.27 | 16.77 | 20.84 | 25.09 | 27.42 | 127.36 |
| Prostate cancer | 20.82 | 23.94 | 27.62 | 29.54 | 37.40 | 47.51 | 54.97 | 164.02 |
| Thyroid cancer | 4.75 | 6.81 | 8.71 | 8.62 | 10.78 | 12.51 | 14.10 | 196.84 |
| Leukaemia | 30.92 | 34.03 | 33.48 | 32.54 | 35.74 | 37.17 | 37.20 | 20.31 |
| Multiple myeloma | 1.73 | 2.06 | 2.16 | 2.25 | 2.74 | 3.32 | 3.65 | 110.98 |
| **Cardiovascular diseases (%)** |  |  |  |  |  |  |  |  |
| Stroke | 1.63 | 1.75 | 1.89 | 2.06 | 2.25 | 2.49 | 2.64 | 61.95 |
| Ischemic heart disease | 1.56 | 1.69 | 1.82 | 2.06 | 2.34 | 2.73 | 2.99 | 91.40 |
| **Chronic respiratory diseases (%)** |  |  |  |  |  |  |  |  |
| Chronic obstructive pulmonary disease | 1.26 | 1.31 | 1.38 | 1.42 | 1.47 | 1.64 | 1.74 | 38.06 |
| Asthma | 3.29 | 2.91 | 2.72 | 2.28 | 2.09 | 2.04 | 2.97 | -9.86 |
| **Digestive diseases (%)** |  |  |  |  |  |  |  |  |
| Gallbladder and biliary diseases | 1.15 | 1.24 | 1.37 | 1.49 | 1.59 | 1.70 | 1.74 | 51.03 |
| Upper digestive system diseases | 5.10 | 5.51 | 6.02 | 6.52 | 6.92 | 7.18 | 7.25 | 42.12 |
| **Neurological disorders (%)** |  |  |  |  |  |  |  |  |
| Alzheimer's disease and other dementias | 0.21 | 0.22 | 0.24 | 0.26 | 0.30 | 0.38 | 0.43 | 104.94 |
| Parkinson's disease | 0.07 | 0.07 | 0.08 | 0.08 | 0.10 | 0.12 | 0.13 | 92.23 |
| **Mental disorders (%)** |  |  |  |  |  |  |  |  |
| Depressive disorders | 2.83 | 3.06 | 3.32 | 3.54 | 3.62 | 3.62 | 3.62 | 27.66 |
| Anxiety disorder | 3.85 | 4.14 | 4.43 | 4.38 | 4.37 | 4.42 | 4.44 | 15.25 |
| **Substance use disorders (%)** |  |  |  |  |  |  |  |  |
| Alcohol use disorders | 0.72 | 0.78 | 0.87 | 0.95 | 1.02 | 1.02 | 1.01 | 40.23 |
| Drug use disorders | 0.65 | 0.69 | 0.77 | 0.81 | 0.75 | 0.62 | 0.57 | -11.79 |
| **Diabetes and kidney diseases (%)** |  |  |  |  |  |  |  |  |
| Diabetes mellitus type 2 | 5.43 | 6.49 | 8.04 | 10.10 | 12.37 | 15.41 | 17.71 | 225.91 |
| Chronic kidney disease | 9.86 | 10.88 | 12.17 | 13.54 | 15.09 | 16.73 | 17.71 | 79.69 |
| **Musculoskeletal disorders (%)** |  |  |  |  |  |  |  |  |
| Osteoarthritis | 2.90 | 3.20 | 3.55 | 4.15 | 4.75 | 5.54 | 6.07 | 109.04 |
| **Micronesia, Federated States of** |  |  |  |  |  |  |  |  |
| **Neoplasms/Cancer (per 100,000 persons)** |  |  |  |  |  |  |  |  |
| Lip and oral cavity cancer | 8.26 | 9.34 | 10.07 | 12.59 | 14.88 | 17.78 | 19.91 | 141.04 |
| Colon and rectum cancer | 27.46 | 31.74 | 35.71 | 45.20 | 55.28 | 67.13 | 78.16 | 184.63 |
| Stomach cancer | 23.31 | 24.40 | 25.39 | 28.16 | 32.28 | 35.76 | 38.89 | 66.84 |
| Pancreatic cancer | 2.09 | 2.68 | 3.32 | 4.36 | 5.24 | 6.15 | 7.09 | 239.23 |
| Non-Hodgkin's lymphoma | 0.00 | 0.05 | 0.07 | 0.08 | 0.09 | 0.09 | 0.27 | N.A |
| Gallbladder and biliary tract cancer | 1.17 | 1.25 | 1.32 | 1.47 | 1.72 | 1.99 | 2.20 | 88.03 |
| Kidney cancer | 4.17 | 5.20 | 6.06 | 8.06 | 9.02 | 10.66 | 12.18 | 192.09 |
| Ovarian cancer | 5.89 | 8.73 | 12.37 | 18.02 | 21.36 | 24.87 | 27.16 | 361.12 |
| Cervical cancer | 70.57 | 81.25 | 88.83 | 107.60 | 122.60 | 134.91 | 141.08 | 99.91 |
| Breast cancer | 112.27 | 135.92 | 172.48 | 238.25 | 301.95 | 362.85 | 411.09 | 266.16 |
| Oesophageal cancer | 3.93 | 4.26 | 4.57 | 5.19 | 5.99 | 6.79 | 7.58 | 92.88 |
| Tracheal, bronchus and lung cancer | 19.89 | 21.38 | 23.95 | 27.73 | 33.95 | 40.69 | 46.32 | 132.88 |
| Prostate cancer | 29.83 | 33.29 | 35.93 | 46.36 | 56.13 | 73.65 | 93.45 | 213.28 |
| Thyroid cancer | 8.69 | 11.42 | 14.04 | 20.15 | 23.19 | 27.61 | 31.00 | 256.73 |
| Leukaemia | 41.87 | 40.34 | 39.12 | 41.05 | 43.46 | 44.55 | 46.09 | 10.08 |
| Multiple myeloma | 2.95 | 3.24 | 3.43 | 4.10 | 4.89 | 5.82 | 6.63 | 124.75 |
| **Cardiovascular diseases (%)** |  |  |  |  |  |  |  |  |
| Stroke | 1.67 | 1.76 | 1.88 | 2.07 | 2.32 | 2.55 | 2.71 | 62.07 |
| Ischemic heart disease | 1.50 | 1.63 | 1.76 | 1.99 | 2.30 | 2.59 | 2.84 | 88.90 |
| **Chronic respiratory diseases (%)** |  |  |  |  |  |  |  |  |
| Chronic obstructive pulmonary disease | 1.36 | 1.42 | 1.46 | 1.61 | 1.64 | 1.76 | 1.85 | 36.07 |
| Asthma | 3.81 | 3.51 | 3.13 | 3.02 | 2.66 | 2.64 | 3.10 | -18.71 |
| **Digestive diseases (%)** |  |  |  |  |  |  |  |  |
| Gallbladder and biliary diseases | 1.18 | 1.25 | 1.33 | 1.44 | 1.55 | 1.67 | 1.71 | 44.41 |
| Upper digestive system diseases | 5.45 | 5.89 | 6.44 | 7.05 | 7.52 | 7.81 | 7.97 | 46.13 |
| **Neurological disorders (%)** |  |  |  |  |  |  |  |  |
| Alzheimer's disease and other dementias | 0.24 | 0.27 | 0.31 | 0.35 | 0.40 | 0.44 | 0.47 | 92.68 |
| Parkinson's disease | 0.08 | 0.09 | 0.10 | 0.11 | 0.13 | 0.14 | 0.16 | 97.12 |
| **Mental disorders (%)** |  |  |  |  |  |  |  |  |
| Depressive disorders | 2.99 | 3.24 | 3.49 | 3.70 | 3.82 | 3.91 | 3.95 | 32.08 |
| Anxiety disorder | 4.17 | 4.40 | 4.52 | 4.48 | 4.54 | 4.58 | 4.58 | 9.74 |
| **Substance use disorders (%)** |  |  |  |  |  |  |  |  |
| Alcohol use disorders | 0.74 | 0.80 | 0.89 | 0.99 | 1.03 | 1.01 | 0.99 | 33.91 |
| Drug use disorders | 0.88 | 0.97 | 1.06 | 1.01 | 0.86 | 0.73 | 0.67 | -23.69 |
| **Diabetes and kidney diseases (%)** |  |  |  |  |  |  |  |  |
| Diabetes mellitus type 2 | 6.04 | 7.72 | 12.04 | 17.19 | 19.07 | 22.39 | 25.28 | 318.49 |
| Chronic kidney disease | 11.51 | 12.98 | 14.73 | 17.09 | 19.37 | 21.27 | 22.47 | 95.17 |
| **Musculoskeletal disorders (%)** |  |  |  |  |  |  |  |  |
| Osteoarthritis | 3.51 | 3.94 | 4.58 | 5.50 | 6.53 | 7.53 | 8.32 | 137.01 |
| **Viet Nam** |  |  |  |  |  |  |  |  |
| **Neoplasms/Cancer (per 100,000 persons)** |  |  |  |  |  |  |  |  |
| Lip and oral cavity cancer | 11.74 | 13.15 | 15.33 | 19.72 | 25.50 | 34.06 | 42.87 | 265.16 |
| Colon and rectum cancer | 22.80 | 27.24 | 35.92 | 56.56 | 87.70 | 125.01 | 170.40 | 647.37 |
| Stomach cancer | 19.29 | 19.54 | 19.14 | 19.25 | 20.07 | 24.19 | 29.18 | 51.27 |
| Pancreatic cancer | 1.09 | 1.22 | 1.49 | 2.30 | 3.45 | 4.56 | 5.83 | 434.86 |
| Non-Hodgkin's lymphoma | 0.00 | 0.00 | 0.00 | 0.43 | 3.92 | 6.00 | 12.40 | N.A |
| Gallbladder and biliary tract cancer | 0.86 | 0.87 | 0.96 | 1.32 | 1.80 | 2.20 | 2.65 | 208.14 |
| Kidney cancer | 2.86 | 3.19 | 3.79 | 5.54 | 8.25 | 11.54 | 15.31 | 435.31 |
| Ovarian cancer | 5.93 | 7.23 | 8.95 | 12.22 | 16.02 | 20.93 | 25.34 | 327.32 |
| Cervical cancer | 30.25 | 33.82 | 38.03 | 46.80 | 56.26 | 66.55 | 77.08 | 154.81 |
| Breast cancer | 82.31 | 87.36 | 106.01 | 151.14 | 203.68 | 270.60 | 348.89 | 323.87 |
| Oesophageal cancer | 2.60 | 2.66 | 2.92 | 3.98 | 5.57 | 6.89 | 8.27 | 218.08 |
| Tracheal, bronchus and lung cancer | 15.04 | 16.00 | 17.67 | 21.56 | 27.30 | 35.16 | 43.78 | 191.09 |
| Prostate cancer | 10.11 | 12.33 | 16.24 | 22.67 | 30.58 | 42.27 | 58.97 | 483.28 |
| Thyroid cancer | 12.08 | 13.77 | 17.38 | 33.52 | 58.09 | 73.63 | 89.28 | 639.07 |
| Leukaemia | 20.30 | 18.65 | 17.23 | 19.13 | 21.50 | 24.23 | 27.74 | 36.65 |
| Multiple myeloma | 1.19 | 1.24 | 1.39 | 1.82 | 2.49 | 3.36 | 4.31 | 262.18 |
| **Cardiovascular diseases (%)** |  |  |  |  |  |  |  |  |
| Stroke | 1.03 | 1.06 | 1.18 | 1.32 | 1.57 | 1.80 | 2.20 | 113.47 |
| Ischemic heart disease | 1.11 | 1.13 | 1.23 | 1.44 | 1.72 | 1.97 | 2.30 | 108.35 |
| **Chronic respiratory diseases (%)** |  |  |  |  |  |  |  |  |
| Chronic obstructive pulmonary disease | 1.47 | 1.62 | 1.88 | 2.29 | 2.74 | 3.31 | 3.90 | 165.78 |
| Asthma | 2.52 | 2.50 | 2.82 | 2.73 | 2.45 | 2.69 | 2.96 | 17.61 |
| **Digestive diseases (%)** |  |  |  |  |  |  |  |  |
| Gallbladder and biliary diseases | 1.19 | 1.18 | 1.21 | 1.28 | 1.46 | 1.61 | 1.81 | 52.02 |
| Upper digestive system diseases | 4.77 | 4.97 | 5.39 | 6.05 | 6.80 | 7.52 | 8.08 | 69.44 |
| **Neurological disorders (%)** |  |  |  |  |  |  |  |  |
| Alzheimer's disease and other dementias | 0.34 | 0.35 | 0.38 | 0.45 | 0.53 | 0.62 | 0.72 | 112.62 |
| Parkinson's disease | 0.06 | 0.06 | 0.07 | 0.08 | 0.10 | 0.12 | 0.14 | 143.65 |
| **Mental disorders (%)** |  |  |  |  |  |  |  |  |
| Depressive disorders | 2.31 | 2.36 | 2.54 | 2.81 | 3.07 | 3.30 | 3.50 | 51.23 |
| Anxiety disorder | 2.21 | 2.26 | 2.38 | 2.26 | 2.38 | 2.77 | 2.79 | 26.54 |
| **Substance use disorders (%)** |  |  |  |  |  |  |  |  |
| Alcohol use disorders | 0.85 | 0.92 | 1.06 | 1.20 | 1.53 | 1.69 | 1.78 | 109.99 |
| Drug use disorders | 0.63 | 0.64 | 0.67 | 0.72 | 0.78 | 0.78 | 0.70 | 11.54 |
| **Diabetes and kidney diseases (%)** |  |  |  |  |  |  |  |  |
| Diabetes mellitus type 2 | 2.49 | 2.80 | 3.12 | 4.11 | 5.21 | 6.79 | 8.69 | 248.71 |
| Chronic kidney disease | 9.57 | 10.14 | 11.30 | 13.20 | 15.37 | 17.72 | 20.09 | 109.98 |
| **Musculoskeletal disorders (%)** |  |  |  |  |  |  |  |  |
| Osteoarthritis | 2.47 | 2.54 | 2.82 | 3.34 | 4.04 | 4.93 | 5.85 | 137.28 |
| **Mongolia** |  |  |  |  |  |  |  |  |
| **Neoplasms/Cancer (per 100,000 persons)** |  |  |  |  |  |  |  |  |
| Lip and oral cavity cancer | 11.57 | 12.21 | 11.13 | 8.73 | 9.24 | 11.31 | 14.24 | 23.08 |
| Colon and rectum cancer | 20.88 | 22.47 | 24.11 | 26.89 | 33.45 | 43.17 | 56.13 | 168.82 |
| Stomach cancer | 65.82 | 71.45 | 72.31 | 68.58 | 71.14 | 78.13 | 92.57 | 40.64 |
| Pancreatic cancer | 1.95 | 2.29 | 2.78 | 3.63 | 4.83 | 6.29 | 8.11 | 315.90 |
| Non-Hodgkin's lymphoma | 0.12 | 0.11 | 0.10 | 0.00 | 0.00 | 0.01 | 2.67 | 2125.00 |
| Gallbladder and biliary tract cancer | 2.77 | 2.95 | 3.11 | 3.16 | 3.47 | 3.90 | 4.77 | 72.20 |
| Kidney cancer | 2.88 | 2.90 | 4.16 | 7.47 | 11.12 | 14.44 | 18.68 | 548.61 |
| Ovarian cancer | 5.64 | 7.24 | 9.47 | 12.65 | 14.84 | 17.68 | 21.48 | 280.85 |
| Cervical cancer | 45.99 | 51.31 | 60.15 | 68.74 | 78.33 | 83.47 | 94.68 | 105.87 |
| Breast cancer | 44.73 | 46.09 | 55.87 | 72.56 | 90.69 | 106.99 | 136.61 | 205.41 |
| Oesophageal cancer | 22.85 | 24.70 | 26.39 | 26.58 | 28.82 | 31.82 | 38.93 | 70.37 |
| Tracheal, bronchus and lung cancer | 25.81 | 27.44 | 26.88 | 24.16 | 25.71 | 31.06 | 39.30 | 52.27 |
| Prostate cancer | 8.08 | 7.77 | 8.52 | 11.24 | 14.86 | 19.27 | 25.36 | 213.86 |
| Thyroid cancer | 2.98 | 3.31 | 4.13 | 6.51 | 9.49 | 12.06 | 15.60 | 423.49 |
| Leukaemia | 15.62 | 15.68 | 13.23 | 14.44 | 14.57 | 14.32 | 15.67 | 0.32 |
| Multiple myeloma | 1.14 | 1.16 | 1.25 | 1.44 | 1.81 | 2.28 | 2.87 | 151.75 |
| **Cardiovascular diseases (%)** |  |  |  |  |  |  |  |  |
| Stroke | 1.15 | 1.24 | 1.40 | 1.48 | 1.60 | 1.87 | 2.02 | 75.94 |
| Ischemic heart disease | 2.61 | 2.54 | 2.72 | 2.94 | 3.40 | 4.11 | 4.93 | 88.78 |
| **Chronic respiratory diseases (%)** |  |  |  |  |  |  |  |  |
| Chronic obstructive pulmonary disease | 1.22 | 1.19 | 1.26 | 1.41 | 1.45 | 1.54 | 1.63 | 33.32 |
| Asthma | 2.30 | 2.12 | 1.95 | 1.84 | 1.73 | 1.71 | 1.90 | -17.57 |
| **Digestive diseases (%)** |  |  |  |  |  |  |  |  |
| Gallbladder and biliary diseases | 3.02 | 3.14 | 3.32 | 3.35 | 3.41 | 3.63 | 3.96 | 31.19 |
| Upper digestive system diseases | 9.11 | 9.82 | 11.09 | 12.53 | 13.92 | 15.10 | 15.82 | 73.61 |
| **Neurological disorders (%)** |  |  |  |  |  |  |  |  |
| Alzheimer's disease and other dementias | 0.30 | 0.29 | 0.28 | 0.28 | 0.32 | 0.41 | 0.50 | 65.47 |
| Parkinson's disease | 0.04 | 0.04 | 0.04 | 0.04 | 0.05 | 0.06 | 0.07 | 58.83 |
| **Mental disorders (%)** |  |  |  |  |  |  |  |  |
| Depressive disorders | 3.76 | 4.12 | 4.62 | 5.08 | 5.30 | 5.52 | 5.62 | 49.57 |
| Anxiety disorder | 2.13 | 2.29 | 2.45 | 2.18 | 2.19 | 2.23 | 2.58 | 21.35 |
| **Substance use disorders (%)** |  |  |  |  |  |  |  |  |
| Alcohol use disorders | 2.25 | 2.69 | 3.75 | 4.85 | 6.33 | 6.23 | 6.81 | 201.80 |
| Drug use disorders | 0.58 | 0.64 | 0.79 | 0.85 | 0.80 | 0.67 | 0.58 | -0.86 |
| **Diabetes and kidney diseases (%)** |  |  |  |  |  |  |  |  |
| Diabetes mellitus type 2 | 1.37 | 1.43 | 1.78 | 2.34 | 3.01 | 3.76 | 4.50 | 227.55 |
| Chronic kidney disease | 9.81 | 10.53 | 11.83 | 13.74 | 15.94 | 17.98 | 19.47 | 98.45 |
| **Musculoskeletal disorders (%)** |  |  |  |  |  |  |  |  |
| Osteoarthritis | 3.42 | 3.41 | 3.73 | 4.23 | 4.99 | 6.28 | 7.76 | 126.71 |
| **Nauru** |  |  |  |  |  |  |  |  |
| **Neoplasms/Cancer (per 100,000 persons)** |  |  |  |  |  |  |  |  |
| Lip and oral cavity cancer | 11.60 | 11.46 | 11.61 | 12.03 | 13.54 | 16.05 | 18.14 | 56.38 |
| Colon and rectum cancer | 44.75 | 42.76 | 41.14 | 39.74 | 44.30 | 59.04 | 73.42 | 64.07 |
| Stomach cancer | 20.67 | 22.11 | 24.49 | 25.69 | 26.61 | 26.82 | 28.17 | 36.28 |
| Pancreatic cancer | 2.15 | 2.36 | 2.50 | 2.67 | 2.82 | 3.61 | 4.45 | 106.98 |
| Non-Hodgkin's lymphoma | 2.72 | 2.72 | 2.59 | 0.09 | 0.80 | 7.08 | 11.30 | 315.44 |
| Gallbladder and biliary tract cancer | 1.02 | 1.03 | 1.09 | 1.11 | 1.17 | 1.25 | 1.36 | 33.33 |
| Kidney cancer | 9.61 | 9.91 | 8.82 | 8.01 | 8.14 | 10.96 | 14.07 | 46.41 |
| Ovarian cancer | 6.87 | 8.09 | 10.01 | 12.09 | 14.04 | 17.99 | 21.20 | 208.59 |
| Cervical cancer | 90.90 | 97.46 | 111.53 | 116.15 | 128.27 | 138.27 | 149.56 | 64.53 |
| Breast cancer | 136.25 | 148.12 | 176.36 | 201.74 | 242.48 | 302.48 | 356.46 | 161.62 |
| Oesophageal cancer | 3.40 | 3.66 | 3.90 | 3.93 | 3.90 | 4.14 | 4.54 | 33.53 |
| Tracheal, bronchus and lung cancer | 21.97 | 21.65 | 22.26 | 22.56 | 24.25 | 27.67 | 30.84 | 40.37 |
| Prostate cancer | 29.49 | 31.06 | 32.08 | 32.30 | 34.42 | 42.77 | 52.55 | 78.20 |
| Thyroid cancer | 14.79 | 17.05 | 20.02 | 21.48 | 24.07 | 30.05 | 35.96 | 143.14 |
| Leukaemia | 54.94 | 57.89 | 54.82 | 50.72 | 46.62 | 43.47 | 44.84 | -18.38 |
| Multiple myeloma | 2.64 | 2.69 | 2.87 | 2.96 | 3.21 | 3.62 | 4.14 | 56.82 |
| **Cardiovascular diseases (%)** |  |  |  |  |  |  |  |  |
| Stroke | 1.44 | 1.47 | 1.58 | 1.63 | 1.75 | 1.87 | 1.96 | 35.94 |
| Ischemic heart disease | 1.12 | 1.12 | 1.14 | 1.18 | 1.27 | 1.42 | 1.57 | 40.52 |
| **Chronic respiratory diseases (%)** |  |  |  |  |  |  |  |  |
| Chronic obstructive pulmonary disease | 1.76 | 1.72 | 1.72 | 1.74 | 1.83 | 2.11 | 2.30 | 30.62 |
| Asthma | 3.34 | 3.01 | 2.95 | 2.68 | 2.31 | 2.16 | 2.90 | -13.23 |
| **Digestive diseases (%)** |  |  |  |  |  |  |  |  |
| Gallbladder and biliary diseases | 0.85 | 0.88 | 0.98 | 1.07 | 1.12 | 1.18 | 1.20 | 40.07 |
| Upper digestive system diseases | 5.07 | 5.11 | 5.43 | 5.82 | 6.30 | 6.79 | 6.98 | 37.66 |
| **Neurological disorders (%)** |  |  |  |  |  |  |  |  |
| Alzheimer's disease and other dementias | 0.15 | 0.14 | 0.14 | 0.13 | 0.14 | 0.15 | 0.17 | 15.94 |
| Parkinson's disease | 0.05 | 0.05 | 0.05 | 0.05 | 0.05 | 0.06 | 0.07 | 25.31 |
| **Mental disorders (%)** |  |  |  |  |  |  |  |  |
| Depressive disorders | 2.96 | 3.01 | 3.18 | 3.44 | 3.74 | 3.96 | 3.98 | 34.39 |
| Anxiety disorder | 3.90 | 3.95 | 4.18 | 4.36 | 4.55 | 4.61 | 4.55 | 16.52 |
| **Substance use disorders (%)** |  |  |  |  |  |  |  |  |
| Alcohol use disorders | 0.82 | 0.81 | 0.86 | 0.91 | 0.99 | 1.09 | 1.12 | 36.95 |
| Drug use disorders | 0.81 | 0.82 | 0.88 | 0.94 | 1.03 | 1.02 | 0.93 | 13.70 |
| **Diabetes and kidney diseases (%)** |  |  |  |  |  |  |  |  |
| Diabetes mellitus type 2 | 5.62 | 6.41 | 7.48 | 8.93 | 10.89 | 13.49 | 15.80 | 181.11 |
| Chronic kidney disease | 11.01 | 11.35 | 12.14 | 13.02 | 14.36 | 16.22 | 17.51 | 59.07 |
| **Musculoskeletal disorders (%)** |  |  |  |  |  |  |  |  |
| Osteoarthritis | 2.87 | 2.88 | 3.05 | 3.33 | 3.65 | 4.23 | 4.74 | 65.48 |

N.A, not available.

# Table S7. Prevalence of noncommunicable diseases among the general population in other countries/areas of the western pacific region, 1990-2019.

| Noncommunicable diseases | Prevalence | | | | | | | Relative change,1990-2019 (%) | |
| --- | --- | --- | --- | --- | --- | --- | --- | --- | --- |
|  | 1990 | 1995 | 2000 | 2005 | 2010 | 2015 | 2019 |  |  |
| **American Samoa (USA)** |  |  |  |  |  |  |  |  | |
| **Neoplasms/Cancer (per 100,000 persons)** |  |  |  |  |  |  |  |  | |
| Lip and oral cavity cancer | 3.18 | 3.34 | 3.04 | 4.04 | 4.47 | 5.14 | 5.23 | 64.47 | |
| Colon and rectum cancer | 32.26 | 35.42 | 41.27 | 43.93 | 52.21 | 63.75 | 68.49 | 112.31 | |
| Stomach cancer | 12.52 | 12.73 | 13.72 | 13.12 | 14.95 | 17.08 | 17.90 | 42.97 | |
| Pancreatic cancer | 1.09 | 1.41 | 1.83 | 2.51 | 2.54 | 3.04 | 3.27 | 200.00 | |
| Non-Hodgkin's lymphoma | 0.04 | 0.05 | 0.05 | 1.18 | 1.92 | 2.96 | 2.80 | 6900.00 | |
| Gallbladder and biliary tract cancer | 0.65 | 0.69 | 0.81 | 0.77 | 0.81 | 0.76 | 0.83 | 27.69 | |
| Kidney cancer | 4.62 | 5.36 | 6.15 | 7.21 | 6.91 | 7.16 | 7.26 | 57.14 | |
| Ovarian cancer | 12.04 | 15.98 | 20.60 | 26.43 | 27.34 | 30.98 | 31.61 | 162.54 | |
| Cervical cancer | 25.49 | 28.93 | 31.77 | 33.92 | 38.26 | 44.06 | 44.76 | 75.60 | |
| Breast cancer | 79.68 | 89.03 | 115.58 | 130.84 | 157.59 | 200.67 | 211.92 | 165.96 | |
| Oesophageal cancer | 0.81 | 0.91 | 1.02 | 1.27 | 1.43 | 1.64 | 1.71 | 111.11 | |
| Tracheal, bronchus and lung cancer | 13.18 | 13.48 | 14.65 | 16.44 | 18.53 | 21.20 | 23.10 | 75.27 | |
| Prostate cancer | 47.90 | 53.72 | 65.79 | 81.11 | 96.45 | 112.43 | 123.20 | 157.20 | |
| Thyroid cancer | 8.78 | 10.64 | 12.86 | 17.96 | 19.47 | 22.16 | 23.26 | 164.92 | |
| Leukaemia | 12.89 | 12.46 | 12.45 | 12.33 | 12.13 | 12.85 | 13.11 | 1.71 | |
| Multiple myeloma | 1.54 | 1.71 | 1.87 | 1.87 | 2.17 | 2.43 | 2.63 | 70.78 | |
| **Cardiovascular diseases (%)** |  |  |  |  |  |  |  |  | |
| Stroke | 1.04 | 1.06 | 1.15 | 1.21 | 1.33 | 1.44 | 1.57 | 50.72 | |
| Ischemic heart disease | 1.10 | 1.20 | 1.34 | 1.53 | 1.73 | 1.97 | 2.16 | 96.56 | |
| **Chronic respiratory diseases (%)** |  |  |  |  |  |  |  |  | |
| Chronic obstructive pulmonary disease | 0.86 | 0.88 | 0.93 | 0.97 | 1.01 | 1.08 | 1.11 | 29.95 | |
| Asthma | 3.42 | 2.67 | 2.69 | 2.65 | 2.98 | 2.74 | 3.34 | -2.33 | |
| **Digestive diseases (%)** |  |  |  |  |  |  |  |  | |
| Gallbladder and biliary diseases | 0.67 | 0.67 | 0.71 | 0.78 | 0.83 | 0.89 | 0.91 | 36.58 | |
| Upper digestive system diseases | 4.30 | 4.41 | 4.55 | 4.69 | 4.84 | 5.06 | 5.29 | 23.08 | |
| **Neurological disorders (%)** |  |  |  |  |  |  |  |  | |
| Alzheimer's disease and other dementias | 0.17 | 0.18 | 0.21 | 0.25 | 0.30 | 0.35 | 0.39 | 132.91 | |
| Parkinson's disease | 0.05 | 0.06 | 0.07 | 0.08 | 0.09 | 0.10 | 0.11 | 94.58 | |
| **Mental disorders (%)** |  |  |  |  |  |  |  |  | |
| Depressive disorders | 2.18 | 2.19 | 2.23 | 2.28 | 2.34 | 2.46 | 2.58 | 18.58 | |
| Anxiety disorder | 3.58 | 3.60 | 3.67 | 3.70 | 3.84 | 4.00 | 4.07 | 13.84 | |
| **Substance use disorders (%)** |  |  |  |  |  |  |  |  | |
| Alcohol use disorders | 0.58 | 0.59 | 0.59 | 0.58 | 0.58 | 0.59 | 0.61 | 4.83 | |
| Drug use disorders | 0.75 | 0.74 | 0.72 | 0.70 | 0.69 | 0.71 | 0.75 | -0.50 | |
| **Diabetes and kidney diseases (%)** |  |  |  |  |  |  |  |  | |
| Diabetes mellitus type 2 | 7.02 | 8.41 | 10.91 | 13.14 | 14.67 | 16.70 | 18.43 | 162.54 | |
| Chronic kidney disease | 6.66 | 7.21 | 7.91 | 8.58 | 9.26 | 10.06 | 10.71 | 60.72 | |
| **Musculoskeletal disorders (%)** |  |  |  |  |  |  |  |  | |
| Osteoarthritis | 2.70 | 2.96 | 3.37 | 3.94 | 4.34 | 4.86 | 5.30 | 96.19 | |
| **New Zealand** |  |  |  |  |  |  |  |  | |
| **Neoplasms/Cancer (per 100,000 persons)** |  |  |  |  |  |  |  |  | |
| Lip and oral cavity cancer | 35.78 | 34.88 | 34.57 | 34.64 | 36.93 | 40.24 | 42.59 | 19.03 | |
| Colon and rectum cancer | 378.37 | 407.45 | 416.76 | 442.50 | 457.30 | 509.35 | 575.51 | 52.10 | |
| Stomach cancer | 21.97 | 23.44 | 23.87 | 23.17 | 22.80 | 24.48 | 26.69 | 21.48 | |
| Pancreatic cancer | 7.59 | 8.25 | 8.40 | 9.61 | 11.12 | 12.56 | 13.76 | 81.29 | |
| Non-Hodgkin's lymphoma | 57.80 | 81.73 | 89.85 | 90.27 | 86.51 | 92.92 | 104.16 | 80.21 | |
| Gallbladder and biliary tract cancer | 4.62 | 4.73 | 5.07 | 5.61 | 6.06 | 6.86 | 7.33 | 58.66 | |
| Kidney cancer | 48.34 | 50.91 | 53.71 | 63.76 | 72.99 | 81.22 | 88.37 | 82.81 | |
| Ovarian cancer | 28.56 | 31.39 | 29.99 | 28.60 | 27.77 | 27.46 | 28.43 | -0.46 | |
| Cervical cancer | 46.93 | 36.50 | 28.43 | 24.82 | 25.24 | 25.36 | 25.24 | -46.22 | |
| Breast cancer | 670.35 | 684.77 | 697.10 | 734.04 | 780.79 | 856.02 | 900.16 | 34.28 | |
| Oesophageal cancer | 9.41 | 11.06 | 11.93 | 13.38 | 14.38 | 15.92 | 17.65 | 87.57 | |
| Tracheal, bronchus and lung cancer | 56.02 | 62.62 | 64.20 | 72.06 | 79.30 | 88.24 | 94.44 | 68.58 | |
| Prostate cancer | 482.44 | 560.07 | 617.88 | 607.64 | 630.01 | 720.47 | 836.88 | 73.47 | |
| Thyroid cancer | 10.91 | 14.41 | 15.52 | 18.92 | 21.70 | 23.41 | 24.63 | 125.76 | |
| Leukaemia | 52.81 | 62.22 | 67.87 | 76.84 | 76.82 | 88.28 | 93.07 | 76.24 | |
| Multiple myeloma | 20.50 | 24.43 | 28.81 | 32.02 | 35.95 | 40.80 | 45.34 | 121.17 | |
| **Cardiovascular diseases (%)** |  |  |  |  |  |  |  |  | |
| Stroke | 0.95 | 0.88 | 0.90 | 0.87 | 0.91 | 0.94 | 1.00 | 5.40 | |
| Ischemic heart disease | 3.43 | 3.42 | 3.34 | 3.37 | 3.49 | 3.80 | 4.05 | 18.20 | |
| **Chronic respiratory diseases (%)** |  |  |  |  |  |  |  |  | |
| Chronic obstructive pulmonary disease | 4.14 | 4.47 | 4.74 | 4.91 | 5.10 | 5.43 | 5.69 | 37.47 | |
| Asthma | 14.35 | 13.44 | 10.22 | 8.05 | 6.73 | 6.26 | 6.01 | -58.12 | |
| **Digestive diseases (%)** |  |  |  |  |  |  |  |  | |
| Gallbladder and biliary diseases | 2.59 | 3.24 | 3.74 | 4.02 | 4.14 | 4.19 | 4.00 | 54.17 | |
| Upper digestive system diseases | 10.39 | 10.57 | 10.77 | 10.99 | 11.22 | 11.54 | 11.77 | 13.24 | |
| **Neurological disorders (%)** |  |  |  |  |  |  |  |  | |
| Alzheimer's disease and other dementias | 0.75 | 0.83 | 0.93 | 0.99 | 1.08 | 1.22 | 1.35 | 78.99 | |
| Parkinson's disease | 0.13 | 0.13 | 0.14 | 0.16 | 0.17 | 0.20 | 0.23 | 77.37 | |
| **Mental disorders (%)** |  |  |  |  |  |  |  |  | |
| Depressive disorders | 3.80 | 3.83 | 3.78 | 3.84 | 3.80 | 3.82 | 3.80 | -0.04 | |
| Anxiety disorder | 7.31 | 7.78 | 7.74 | 7.75 | 7.49 | 7.42 | 7.38 | 0.85 | |
| **Substance use disorders (%)** |  |  |  |  |  |  |  |  | |
| Alcohol use disorders | 2.40 | 2.46 | 1.65 | 1.63 | 2.19 | 3.25 | 3.24 | 35.02 | |
| Drug use disorders | 1.67 | 1.65 | 1.64 | 1.66 | 1.54 | 1.51 | 1.47 | -11.81 | |
| **Diabetes and kidney diseases (%)** |  |  |  |  |  |  |  |  | |
| Diabetes mellitus type 2 | 1.97 | 2.32 | 2.45 | 2.25 | 2.83 | 3.74 | 4.74 | 140.19 | |
| Chronic kidney disease | 7.05 | 7.50 | 8.06 | 8.43 | 9.01 | 9.73 | 10.30 | 46.24 | |
| **Musculoskeletal disorders (%)** |  |  |  |  |  |  |  |  | |
| Osteoarthritis | 8.66 | 9.14 | 9.87 | 10.56 | 11.42 | 12.54 | 13.37 | 54.37 | |
| **Brunei Darussalam** |  |  |  |  |  |  |  |  | |
| **Neoplasms/Cancer (per 100,000 persons)** |  |  |  |  |  |  |  |  | |
| Lip and oral cavity cancer | 8.42 | 7.97 | 8.06 | 8.42 | 9.30 | 11.34 | 12.89 | 53.09 | |
| Colon and rectum cancer | 65.73 | 63.58 | 67.13 | 89.63 | 112.55 | 139.32 | 166.00 | 152.55 | |
| Stomach cancer | 24.02 | 22.01 | 18.69 | 18.54 | 18.39 | 20.18 | 23.99 | -0.12 | |
| Pancreatic cancer | 1.77 | 1.77 | 1.88 | 2.34 | 3.01 | 3.94 | 4.72 | 166.67 | |
| Non-Hodgkin's lymphoma | 15.37 | 16.57 | 17.21 | 23.14 | 26.20 | 31.13 | 35.01 | 127.78 | |
| Gallbladder and biliary tract cancer | 1.91 | 1.69 | 1.67 | 1.91 | 2.16 | 2.63 | 3.00 | 57.07 | |
| Kidney cancer | 7.11 | 8.01 | 8.90 | 12.26 | 15.65 | 19.87 | 22.73 | 219.69 | |
| Ovarian cancer | 12.26 | 14.31 | 17.05 | 22.41 | 27.17 | 31.28 | 33.74 | 175.20 | |
| Cervical cancer | 71.58 | 67.34 | 58.92 | 62.94 | 64.52 | 69.95 | 76.17 | 6.41 | |
| Breast cancer | 119.03 | 121.37 | 133.32 | 172.53 | 217.96 | 271.89 | 310.16 | 160.57 | |
| Oesophageal cancer | 2.77 | 2.60 | 2.45 | 2.53 | 2.75 | 3.27 | 3.98 | 43.68 | |
| Tracheal, bronchus and lung cancer | 17.68 | 16.49 | 17.73 | 21.41 | 25.24 | 30.37 | 36.27 | 105.15 | |
| Prostate cancer | 14.58 | 14.73 | 18.05 | 25.79 | 33.06 | 39.73 | 50.35 | 245.34 | |
| Thyroid cancer | 15.67 | 16.84 | 18.49 | 24.90 | 30.38 | 33.42 | 37.84 | 141.48 | |
| Leukaemia | 25.85 | 23.83 | 23.26 | 25.91 | 27.21 | 32.29 | 33.43 | 29.32 | |
| Multiple myeloma | 1.91 | 1.77 | 2.07 | 3.02 | 4.11 | 5.33 | 6.19 | 224.08 | |
| **Cardiovascular diseases (%)** |  |  |  |  |  |  |  |  | |
| Stroke | 0.99 | 0.93 | 0.93 | 0.91 | 0.97 | 1.02 | 1.20 | 21.26 | |
| Ischemic heart disease | 0.56 | 0.56 | 0.59 | 0.61 | 0.70 | 0.77 | 0.90 | 62.19 | |
| **Chronic respiratory diseases (%)** |  |  |  |  |  |  |  |  | |
| Chronic obstructive pulmonary disease | 1.37 | 1.45 | 1.48 | 1.53 | 1.59 | 1.70 | 1.86 | 36.04 | |
| Asthma | 4.27 | 4.36 | 4.17 | 4.05 | 3.89 | 3.90 | 4.06 | -5.07 | |
| **Digestive diseases (%)** |  |  |  |  |  |  |  |  | |
| Gallbladder and biliary diseases | 3.22 | 3.38 | 3.43 | 3.53 | 3.70 | 3.96 | 4.13 | 28.05 | |
| Upper digestive system diseases | 5.12 | 5.34 | 5.54 | 5.78 | 6.11 | 6.46 | 6.76 | 32.10 | |
| **Neurological disorders (%)** |  |  |  |  |  |  |  |  | |
| Alzheimer's disease and other dementias | 0.12 | 0.13 | 0.13 | 0.14 | 0.16 | 0.19 | 0.23 | 84.19 | |
| Parkinson's disease | 0.02 | 0.03 | 0.03 | 0.03 | 0.04 | 0.04 | 0.05 | 112.13 | |
| **Mental disorders (%)** |  |  |  |  |  |  |  |  | |
| Depressive disorders | 1.44 | 1.43 | 1.47 | 1.52 | 1.54 | 1.63 | 1.71 | 18.90 | |
| Anxiety disorder | 2.77 | 2.84 | 2.92 | 2.98 | 3.01 | 3.04 | 3.04 | 9.56 | |
| **Substance use disorders (%)** |  |  |  |  |  |  |  |  | |
| Alcohol use disorders | 0.94 | 0.92 | 0.92 | 0.94 | 0.98 | 1.00 | 1.05 | 11.90 | |
| Drug use disorders | 0.95 | 0.93 | 0.93 | 0.93 | 0.94 | 0.94 | 0.93 | -1.45 | |
| **Diabetes and kidney diseases (%)** |  |  |  |  |  |  |  |  | |
| Diabetes mellitus type 2 | 4.81 | 5.49 | 6.20 | 7.47 | 8.64 | 9.69 | 10.46 | 117.46 | |
| Chronic kidney disease | 5.23 | 5.58 | 5.97 | 6.42 | 6.84 | 7.38 | 7.86 | 50.22 | |
| **Musculoskeletal disorders (%)** |  |  |  |  |  |  |  |  | |
| Osteoarthritis | 3.38 | 3.65 | 4.08 | 4.67 | 5.35 | 6.19 | 6.93 | 104.70 | |
| **Niue** |  |  |  |  |  |  |  |  | |
| **Neoplasms/Cancer (per 100,000 persons)** |  |  |  |  |  |  |  |  | |
| Lip and oral cavity cancer | 11.69 | 13.20 | 14.69 | 16.29 | 17.71 | 18.09 | 18.78 | 60.65 | |
| Colon and rectum cancer | 50.76 | 59.74 | 72.97 | 85.01 | 97.29 | 101.40 | 107.31 | 111.41 | |
| Stomach cancer | 15.67 | 17.47 | 19.38 | 20.61 | 20.35 | 19.49 | 19.75 | 26.04 | |
| Pancreatic cancer | 2.55 | 3.20 | 4.14 | 5.21 | 5.49 | 5.79 | 6.11 | 139.61 | |
| Non-Hodgkin's lymphoma | 0.51 | 1.55 | 3.33 | 4.71 | 7.99 | 9.02 | 9.85 | 1831.37 | |
| Gallbladder and biliary tract cancer | 1.35 | 1.46 | 1.60 | 1.69 | 1.70 | 1.64 | 1.65 | 22.22 | |
| Kidney cancer | 9.38 | 11.60 | 14.57 | 16.38 | 17.36 | 17.90 | 18.78 | 100.21 | |
| Ovarian cancer | 8.90 | 11.57 | 14.81 | 18.01 | 19.96 | 21.56 | 22.77 | 155.84 | |
| Cervical cancer | 61.08 | 71.90 | 73.73 | 72.43 | 78.26 | 77.31 | 77.79 | 27.36 | |
| Breast cancer | 174.14 | 203.06 | 248.25 | 288.99 | 335.10 | 347.28 | 358.87 | 106.08 | |
| Oesophageal cancer | 3.16 | 3.65 | 4.25 | 4.62 | 4.46 | 4.42 | 4.57 | 44.62 | |
| Tracheal, bronchus and lung cancer | 24.48 | 27.40 | 32.53 | 36.67 | 38.58 | 38.69 | 40.00 | 63.40 | |
| Prostate cancer | 64.46 | 79.31 | 111.85 | 143.20 | 166.06 | 163.52 | 172.71 | 167.93 | |
| Thyroid cancer | 13.41 | 18.08 | 22.97 | 26.36 | 29.45 | 30.27 | 31.64 | 135.94 | |
| Leukaemia | 28.76 | 29.80 | 31.04 | 31.12 | 30.55 | 28.62 | 27.93 | -2.89 | |
| Multiple myeloma | 2.56 | 2.95 | 3.38 | 3.66 | 3.76 | 3.82 | 4.01 | 56.64 | |
| **Cardiovascular diseases (%)** |  |  |  |  |  |  |  |  | |
| Stroke | 1.66 | 1.72 | 1.94 | 2.05 | 2.09 | 2.06 | 2.13 | 27.95 | |
| Ischemic heart disease | 2.26 | 2.39 | 2.73 | 3.07 | 3.13 | 3.13 | 3.16 | 40.11 | |
| **Chronic respiratory diseases (%)** |  |  |  |  |  |  |  |  | |
| Chronic obstructive pulmonary disease | 3.00 | 3.14 | 3.49 | 3.89 | 4.03 | 4.01 | 3.98 | 32.60 | |
| Asthma | 3.71 | 2.73 | 2.76 | 2.76 | 2.87 | 2.84 | 3.52 | -5.29 | |
| **Digestive diseases (%)** |  |  |  |  |  |  |  |  | |
| Gallbladder and biliary diseases | 0.82 | 0.85 | 0.92 | 0.96 | 0.97 | 0.98 | 0.99 | 21.24 | |
| Upper digestive system diseases | 5.03 | 5.35 | 5.68 | 5.96 | 6.07 | 6.15 | 6.26 | 24.40 | |
| **Neurological disorders (%)** |  |  |  |  |  |  |  |  | |
| Alzheimer's disease and other dementias | 0.64 | 0.60 | 0.63 | 0.71 | 0.74 | 0.74 | 0.75 | 18.11 | |
| Parkinson's disease | 0.14 | 0.14 | 0.16 | 0.19 | 0.20 | 0.19 | 0.19 | 33.45 | |
| **Mental disorders (%)** |  |  |  |  |  |  |  |  | |
| Depressive disorders | 2.81 | 3.00 | 3.12 | 3.24 | 3.31 | 3.35 | 3.41 | 21.27 | |
| Anxiety disorder | 3.76 | 3.90 | 3.99 | 4.00 | 4.07 | 4.14 | 4.20 | 11.77 | |
| **Substance use disorders (%)** |  |  |  |  |  |  |  |  | |
| Alcohol use disorders | 0.64 | 0.69 | 0.72 | 0.75 | 0.77 | 0.78 | 0.79 | 22.83 | |
| Drug use disorders | 0.67 | 0.69 | 0.67 | 0.69 | 0.69 | 0.67 | 0.69 | 3.39 | |
| **Diabetes and kidney diseases (%)** |  |  |  |  |  |  |  |  | |
| Diabetes mellitus type 2 | 7.87 | 9.73 | 12.52 | 15.17 | 17.07 | 18.83 | 20.07 | 155.04 | |
| Chronic kidney disease | 9.04 | 9.94 | 11.27 | 12.54 | 13.19 | 13.54 | 13.94 | 54.22 | |
| **Musculoskeletal disorders (%)** |  |  |  |  |  |  |  |  | |
| Osteoarthritis | 4.67 | 5.16 | 6.01 | 6.79 | 6.93 | 7.13 | 7.37 | 57.93 | |
| **Cambodia** |  |  |  |  |  |  |  |  | |
| **Neoplasms/Cancer (per 100,000 persons)** |  |  |  |  |  |  |  |  | |
| Lip and oral cavity cancer | 4.91 | 5.20 | 5.53 | 6.60 | 7.80 | 9.60 | 11.36 | 131.36 | |
| Colon and rectum cancer | 12.83 | 14.18 | 16.39 | 21.58 | 27.28 | 35.26 | 43.84 | 241.70 | |
| Stomach cancer | 8.57 | 7.97 | 7.42 | 7.27 | 7.35 | 7.70 | 8.29 | -3.27 | |
| Pancreatic cancer | 0.71 | 0.75 | 0.83 | 1.03 | 1.29 | 1.61 | 1.93 | 171.83 | |
| Non-Hodgkin's lymphoma | 0.00 | 0.00 | 0.00 | 0.00 | 0.00 | 0.54 | 0.59 |  | |
| Gallbladder and biliary tract cancer | 0.73 | 0.71 | 0.69 | 0.74 | 0.83 | 0.97 | 1.10 | 50.68 | |
| Kidney cancer | 3.02 | 3.03 | 3.14 | 4.06 | 4.67 | 5.89 | 7.14 | 136.42 | |
| Ovarian cancer | 4.59 | 5.65 | 7.33 | 10.16 | 12.38 | 15.34 | 17.65 | 284.53 | |
| Cervical cancer | 29.34 | 30.34 | 30.61 | 33.35 | 33.71 | 37.01 | 40.48 | 37.97 | |
| Breast cancer | 33.26 | 36.10 | 41.72 | 51.36 | 60.26 | 73.20 | 86.01 | 158.60 | |
| Oesophageal cancer | 2.25 | 2.18 | 2.11 | 2.18 | 2.39 | 2.68 | 2.98 | 32.44 | |
| Tracheal, bronchus and lung cancer | 10.43 | 10.48 | 10.75 | 11.73 | 13.55 | 15.70 | 17.84 | 71.05 | |
| Prostate cancer | 6.48 | 7.05 | 8.32 | 11.83 | 15.35 | 20.58 | 27.08 | 317.90 | |
| Thyroid cancer | 6.54 | 7.59 | 8.87 | 11.85 | 13.60 | 17.25 | 20.92 | 219.88 | |
| Leukaemia | 73.52 | 63.05 | 51.92 | 49.00 | 39.87 | 37.46 | 35.48 | -51.74 | |
| Multiple myeloma | 0.67 | 0.69 | 0.73 | 0.86 | 1.05 | 1.33 | 1.59 | 137.31 | |
| **Cardiovascular diseases (%)** |  |  |  |  |  |  |  |  | |
| Stroke | 0.72 | 0.72 | 0.77 | 0.81 | 0.89 | 0.94 | 1.03 | 42.82 | |
| Ischemic heart disease | 0.69 | 0.69 | 0.76 | 0.83 | 0.92 | 1.01 | 1.10 | 60.11 | |
| **Chronic respiratory diseases (%)** |  |  |  |  |  |  |  |  | |
| Chronic obstructive pulmonary disease | 0.91 | 0.93 | 1.00 | 1.15 | 1.26 | 1.46 | 1.64 | 80.27 | |
| Asthma | 2.19 | 2.08 | 2.02 | 2.06 | 2.03 | 2.08 | 2.33 | 6.02 | |
| **Digestive diseases (%)** |  |  |  |  |  |  |  |  | |
| Gallbladder and biliary diseases | 1.21 | 1.22 | 1.22 | 1.23 | 1.23 | 1.26 | 1.30 | 7.28 | |
| Upper digestive system diseases | 3.93 | 4.01 | 4.18 | 4.47 | 4.78 | 5.07 | 5.26 | 33.91 | |
| **Neurological disorders (%)** |  |  |  |  |  |  |  |  | |
| Alzheimer's disease and other dementias | 0.16 | 0.17 | 0.18 | 0.21 | 0.24 | 0.28 | 0.31 | 90.86 | |
| Parkinson's disease | 0.03 | 0.03 | 0.03 | 0.04 | 0.04 | 0.05 | 0.06 | 86.40 | |
| **Mental disorders (%)** |  |  |  |  |  |  |  |  | |
| Depressive disorders | 2.36 | 2.41 | 2.50 | 2.59 | 2.66 | 2.74 | 2.80 | 18.61 | |
| Anxiety disorder | 3.61 | 3.71 | 3.89 | 4.07 | 4.26 | 4.39 | 4.47 | 23.81 | |
| **Substance use disorders (%)** |  |  |  |  |  |  |  |  | |
| Alcohol use disorders | 0.55 | 0.57 | 0.59 | 0.63 | 0.69 | 0.74 | 0.77 | 42.11 | |
| Drug use disorders | 0.42 | 0.42 | 0.40 | 0.44 | 0.47 | 0.47 | 0.47 | 12.00 | |
| **Diabetes and kidney diseases (%)** |  |  |  |  |  |  |  |  | |
| Diabetes mellitus type 2 | 1.13 | 1.26 | 1.53 | 1.86 | 2.42 | 3.27 | 4.23 | 272.87 | |
| Chronic kidney disease | 4.94 | 5.14 | 5.47 | 6.04 | 6.66 | 7.34 | 7.94 | 60.65 | |
| **Musculoskeletal disorders (%)** |  |  |  |  |  |  |  |  | |
| Osteoarthritis | 1.66 | 1.71 | 1.85 | 2.11 | 2.41 | 2.71 | 2.96 | 78.13 | |
| **Northern Mariana Islands, Commonwealth of the (USA)** |  |  |  |  |  |  |  |  | |
| **Neoplasms/Cancer (per 100,000 persons)** |  |  |  |  |  |  |  |  | |
| Lip and oral cavity cancer | 16.55 | 17.53 | 18.65 | 42.27 | 54.40 | 64.32 | 71.22 | 330.33 | |
| Colon and rectum cancer | 54.96 | 62.64 | 62.59 | 79.17 | 107.97 | 137.60 | 178.85 | 225.42 | |
| Stomach cancer | 13.98 | 15.50 | 15.32 | 14.56 | 18.04 | 21.97 | 25.55 | 82.76 | |
| Pancreatic cancer | 1.07 | 1.29 | 1.43 | 3.44 | 4.68 | 6.14 | 7.41 | 592.52 | |
| Non-Hodgkin's lymphoma | 2.62 | 4.56 | 5.03 | 10.39 | 11.94 | 13.66 | 17.10 | 552.67 | |
| Gallbladder and biliary tract cancer | 0.52 | 0.55 | 0.54 | 0.79 | 1.01 | 1.06 | 1.31 | 151.92 | |
| Kidney cancer | 14.13 | 17.52 | 19.40 | 28.26 | 28.39 | 25.72 | 28.16 | 99.29 | |
| Ovarian cancer | 6.24 | 8.84 | 10.66 | 16.98 | 18.93 | 22.57 | 23.10 | 270.19 | |
| Cervical cancer | 94.53 | 106.11 | 110.29 | 122.14 | 122.64 | 134.51 | 133.97 | 41.72 | |
| Breast cancer | 133.54 | 166.46 | 179.16 | 229.22 | 281.41 | 372.48 | 402.00 | 201.03 | |
| Oesophageal cancer | 0.99 | 1.09 | 1.16 | 2.26 | 3.82 | 5.19 | 5.94 | 500.00 | |
| Tracheal, bronchus and lung cancer | 23.80 | 23.38 | 22.68 | 27.49 | 37.64 | 48.77 | 61.64 | 158.99 | |
| Prostate cancer | 32.58 | 40.53 | 40.67 | 67.91 | 100.95 | 140.83 | 195.73 | 500.77 | |
| Thyroid cancer | 10.44 | 13.90 | 16.40 | 24.80 | 31.50 | 36.29 | 41.13 | 293.97 | |
| Leukaemia | 22.97 | 23.41 | 24.71 | 24.26 | 23.65 | 25.19 | 28.19 | 22.73 | |
| Multiple myeloma | 3.01 | 3.31 | 3.08 | 3.54 | 5.14 | 6.09 | 7.92 | 163.12 | |
| **Cardiovascular diseases (%)** |  |  |  |  |  |  |  |  | |
| Stroke | 1.05 | 1.02 | 1.01 | 1.15 | 1.44 | 1.74 | 2.08 | 98.76 | |
| Ischemic heart disease | 0.85 | 0.92 | 0.88 | 1.19 | 1.68 | 2.26 | 2.80 | 229.75 | |
| **Chronic respiratory diseases (%)** |  |  |  |  |  |  |  |  | |
| Chronic obstructive pulmonary disease | 0.73 | 0.76 | 0.72 | 0.75 | 0.82 | 0.94 | 1.11 | 50.45 | |
| Asthma | 3.66 | 2.96 | 2.76 | 3.22 | 3.12 | 2.95 | 3.33 | -9.10 | |
| **Digestive diseases (%)** |  |  |  |  |  |  |  |  | |
| Gallbladder and biliary diseases | 0.72 | 0.74 | 0.77 | 0.86 | 0.99 | 1.14 | 1.22 | 69.22 | |
| Upper digestive system diseases | 5.04 | 5.06 | 5.39 | 5.41 | 5.83 | 6.24 | 6.68 | 32.41 | |
| **Neurological disorders (%)** |  |  |  |  |  |  |  |  | |
| Alzheimer's disease and other dementias | 0.10 | 0.12 | 0.11 | 0.16 | 0.23 | 0.34 | 0.45 | 330.19 | |
| Parkinson's disease | 0.05 | 0.05 | 0.04 | 0.06 | 0.09 | 0.12 | 0.15 | 232.38 | |
| **Mental disorders (%)** |  |  |  |  |  |  |  |  | |
| Depressive disorders | 2.58 | 2.52 | 2.69 | 2.70 | 2.91 | 3.14 | 3.33 | 28.83 | |
| Anxiety disorder | 3.84 | 3.74 | 4.03 | 3.83 | 4.06 | 4.31 | 4.39 | 14.43 | |
| **Substance use disorders (%)** |  |  |  |  |  |  |  |  | |
| Alcohol use disorders | 1.11 | 1.10 | 1.10 | 1.09 | 1.08 | 1.03 | 1.05 | -5.22 | |
| Drug use disorders | 0.91 | 0.85 | 0.96 | 0.78 | 0.73 | 0.73 | 0.78 | -14.29 | |
| **Diabetes and kidney diseases (%)** |  |  |  |  |  |  |  |  | |
| Diabetes mellitus type 2 | 3.88 | 4.34 | 5.06 | 6.63 | 8.90 | 11.17 | 13.49 | 247.99 | |
| Chronic kidney disease | 7.62 | 8.22 | 8.79 | 9.74 | 11.25 | 12.75 | 13.84 | 81.77 | |
| **Musculoskeletal disorders (%)** |  |  |  |  |  |  |  |  | |
| Osteoarthritis | 2.38 | 2.50 | 2.45 | 3.54 | 4.95 | 6.55 | 7.83 | 228.63 | |
| **Palau** |  |  |  |  |  |  |  |  | |
| **Neoplasms/Cancer (per 100,000 persons)** |  |  |  |  |  |  |  |  | |
| Lip and oral cavity cancer | 87.11 | 95.47 | 98.99 | 104.92 | 119.50 | 133.93 | 149.81 | 71.98 | |
| Colon and rectum cancer | 41.82 | 47.50 | 51.87 | 59.02 | 71.20 | 84.69 | 101.11 | 141.77 | |
| Stomach cancer | 13.98 | 15.03 | 17.18 | 17.81 | 19.68 | 21.71 | 24.15 | 72.75 | |
| Pancreatic cancer | 3.73 | 4.43 | 4.92 | 6.11 | 7.00 | 8.42 | 10.05 | 169.44 | |
| Non-Hodgkin's lymphoma | 0.48 | 1.62 | 2.33 | 2.10 | 2.20 | 2.77 | 3.95 | 722.92 | |
| Gallbladder and biliary tract cancer | 0.61 | 0.63 | 0.67 | 0.73 | 0.85 | 0.98 | 1.11 | 81.97 | |
| Kidney cancer | 4.97 | 6.10 | 7.47 | 8.56 | 9.04 | 10.25 | 11.99 | 141.25 | |
| Ovarian cancer | 4.75 | 6.20 | 7.10 | 8.94 | 10.06 | 11.57 | 12.95 | 172.63 | |
| Cervical cancer | 163.46 | 190.82 | 181.25 | 188.93 | 208.28 | 219.11 | 227.56 | 39.21 | |
| Breast cancer | 168.83 | 199.17 | 214.89 | 256.49 | 309.61 | 357.28 | 404.11 | 139.36 | |
| Oesophageal cancer | 2.10 | 2.38 | 2.73 | 3.04 | 3.39 | 3.92 | 4.53 | 115.71 | |
| Tracheal, bronchus and lung cancer | 27.65 | 29.09 | 31.86 | 35.15 | 41.91 | 49.34 | 57.26 | 107.09 | |
| Prostate cancer | 72.28 | 81.04 | 91.99 | 95.99 | 117.87 | 145.56 | 180.95 | 150.35 | |
| Thyroid cancer | 23.98 | 30.64 | 39.24 | 45.70 | 48.79 | 53.87 | 61.01 | 154.42 | |
| Leukaemia | 9.31 | 8.75 | 8.74 | 9.31 | 9.99 | 10.52 | 11.39 | 22.34 | |
| Multiple myeloma | 0.99 | 1.06 | 1.15 | 1.31 | 1.53 | 1.83 | 2.19 | 121.21 | |
| **Cardiovascular diseases (%)** |  |  |  |  |  |  |  |  | |
| Stroke | 1.33 | 1.38 | 1.49 | 1.58 | 1.80 | 2.02 | 2.30 | 73.03 | |
| Ischemic heart disease | 1.44 | 1.54 | 1.64 | 1.80 | 2.08 | 2.41 | 2.73 | 89.20 | |
| **Chronic respiratory diseases (%)** |  |  |  |  |  |  |  |  | |
| Chronic obstructive pulmonary disease | 2.18 | 2.30 | 2.38 | 2.57 | 2.83 | 3.18 | 3.51 | 60.98 | |
| Asthma | 3.90 | 3.47 | 3.39 | 3.20 | 2.99 | 2.93 | 3.18 | -18.48 | |
| **Digestive diseases (%)** |  |  |  |  |  |  |  |  | |
| Gallbladder and biliary diseases | 0.75 | 0.79 | 0.80 | 0.86 | 0.91 | 0.99 | 1.07 | 42.43 | |
| Upper digestive system diseases | 5.03 | 5.43 | 5.69 | 5.90 | 6.15 | 6.50 | 6.82 | 35.72 | |
| **Neurological disorders (%)** |  |  |  |  |  |  |  |  | |
| Alzheimer's disease and other dementias | 0.25 | 0.26 | 0.26 | 0.28 | 0.33 | 0.38 | 0.43 | 71.45 | |
| Parkinson's disease | 0.09 | 0.09 | 0.09 | 0.10 | 0.12 | 0.13 | 0.15 | 69.97 | |
| **Mental disorders (%)** |  |  |  |  |  |  |  |  | |
| Depressive disorders | 2.91 | 3.12 | 3.19 | 3.31 | 3.43 | 3.55 | 3.67 | 25.77 | |
| Anxiety disorder | 3.86 | 3.91 | 3.90 | 4.00 | 4.11 | 4.18 | 4.25 | 10.14 | |
| **Substance use disorders (%)** |  |  |  |  |  |  |  |  | |
| Alcohol use disorders | 0.75 | 0.83 | 0.94 | 0.95 | 0.92 | 0.94 | 0.97 | 29.63 | |
| Drug use disorders | 0.83 | 0.82 | 0.82 | 0.82 | 0.79 | 0.73 | 0.71 | -14.56 | |
| **Diabetes and kidney diseases (%)** |  |  |  |  |  |  |  |  | |
| Diabetes mellitus type 2 | 5.24 | 6.54 | 8.28 | 10.28 | 12.27 | 14.96 | 17.36 | 231.11 | |
| Chronic kidney disease | 8.20 | 9.42 | 10.60 | 11.43 | 12.44 | 13.22 | 14.32 | 74.70 | |
| **Musculoskeletal disorders (%)** |  |  |  |  |  |  |  |  | |
| Osteoarthritis | 3.34 | 3.68 | 4.04 | 4.80 | 5.48 | 6.44 | 7.38 | 121.18 | |
| **Cook Islands** |  |  |  |  |  |  |  |  | |
| **Neoplasms/Cancer (per 100,000 persons)** |  |  |  |  |  |  |  |  | |
| Lip and oral cavity cancer | 7.76 | 7.86 | 8.35 | 8.83 | 11.01 | 13.30 | 14.94 | 92.53 | |
| Colon and rectum cancer | 29.63 | 33.59 | 40.03 | 40.24 | 50.17 | 63.64 | 76.06 | 156.70 | |
| Stomach cancer | 10.44 | 10.56 | 11.55 | 10.88 | 12.34 | 14.52 | 15.65 | 49.90 | |
| Pancreatic cancer | 1.86 | 2.28 | 2.99 | 3.09 | 3.52 | 4.52 | 5.17 | 177.96 | |
| Non-Hodgkin's lymphoma | 0.14 | 0.75 | 1.28 | 2.29 | 4.49 | 6.93 | 9.32 | 6557.14 | |
| Gallbladder and biliary tract cancer | 1.16 | 1.17 | 1.36 | 1.11 | 1.25 | 1.33 | 1.46 | 25.86 | |
| Kidney cancer | 6.14 | 6.82 | 7.92 | 8.58 | 9.57 | 11.16 | 12.83 | 108.96 | |
| Ovarian cancer | 4.23 | 5.15 | 6.50 | 8.29 | 10.59 | 13.23 | 14.58 | 244.68 | |
| Cervical cancer | 24.13 | 22.02 | 20.81 | 23.11 | 26.93 | 29.13 | 30.10 | 24.74 | |
| Breast cancer | 224.48 | 247.02 | 289.85 | 307.29 | 388.90 | 463.65 | 510.24 | 127.30 | |
| Oesophageal cancer | 3.01 | 3.36 | 3.89 | 4.05 | 4.37 | 4.97 | 5.48 | 82.06 | |
| Tracheal, bronchus and lung cancer | 23.78 | 24.94 | 28.41 | 29.49 | 32.75 | 37.48 | 42.34 | 78.05 | |
| Prostate cancer | 167.11 | 207.93 | 259.25 | 284.64 | 343.49 | 398.06 | 471.10 | 181.91 | |
| Thyroid cancer | 15.28 | 17.19 | 21.85 | 18.17 | 17.21 | 21.30 | 24.13 | 57.92 | |
| Leukaemia | 14.99 | 13.56 | 13.01 | 12.07 | 11.93 | 12.35 | 13.10 | -12.61 | |
| Multiple myeloma | 1.29 | 1.41 | 1.62 | 1.71 | 2.04 | 2.47 | 2.82 | 118.60 | |
| **Cardiovascular diseases (%)** |  |  |  |  |  |  |  |  | |
| Stroke | 1.25 | 1.32 | 1.52 | 1.56 | 1.78 | 2.01 | 2.25 | 79.86 | |
| Ischemic heart disease | 1.56 | 1.78 | 2.12 | 2.29 | 2.69 | 3.15 | 3.46 | 120.82 | |
| **Chronic respiratory diseases (%)** |  |  |  |  |  |  |  |  | |
| Chronic obstructive pulmonary disease | 2.30 | 2.53 | 2.90 | 3.08 | 3.53 | 4.05 | 4.40 | 90.95 | |
| Asthma | 3.97 | 3.00 | 3.09 | 3.10 | 3.13 | 3.10 | 3.54 | -10.61 | |
| **Digestive diseases (%)** |  |  |  |  |  |  |  |  | |
| Gallbladder and biliary diseases | 0.67 | 0.69 | 0.76 | 0.78 | 0.84 | 0.91 | 0.96 | 41.88 | |
| Upper digestive system diseases | 4.68 | 4.92 | 5.36 | 5.47 | 5.82 | 6.16 | 6.39 | 36.55 | |
| **Neurological disorders (%)** |  |  |  |  |  |  |  |  | |
| Alzheimer's disease and other dementias | 0.29 | 0.33 | 0.39 | 0.44 | 0.55 | 0.68 | 0.78 | 163.69 | |
| Parkinson's disease | 0.09 | 0.09 | 0.12 | 0.13 | 0.15 | 0.18 | 0.20 | 121.08 | |
| **Mental disorders (%)** |  |  |  |  |  |  |  |  | |
| Depressive disorders | 2.75 | 2.87 | 3.05 | 3.10 | 3.26 | 3.39 | 3.48 | 26.56 | |
| Anxiety disorder | 3.72 | 3.79 | 3.89 | 3.93 | 4.07 | 4.19 | 4.28 | 14.99 | |
| **Substance use disorders (%)** |  |  |  |  |  |  |  |  | |
| Alcohol use disorders | 0.67 | 0.70 | 0.77 | 0.78 | 0.79 | 0.79 | 0.79 | 17.24 | |
| Drug use disorders | 0.77 | 0.77 | 0.75 | 0.75 | 0.73 | 0.69 | 0.69 | -10.11 | |
| **Diabetes and kidney diseases (%)** |  |  |  |  |  |  |  |  | |
| Diabetes mellitus type 2 | 5.06 | 6.33 | 8.31 | 9.76 | 11.96 | 14.41 | 16.19 | 219.79 | |
| Chronic kidney disease | 7.33 | 8.16 | 9.52 | 10.19 | 11.50 | 12.87 | 13.97 | 90.58 | |
| **Musculoskeletal disorders (%)** |  |  |  |  |  |  |  |  | |
| Osteoarthritis | 3.54 | 4.05 | 4.92 | 5.40 | 6.18 | 7.19 | 7.92 | 123.78 | |
| **Papua New Guinea** |  |  |  |  |  |  |  |  | |
| **Neoplasms/Cancer (per 100,000 persons)** |  |  |  |  |  |  |  |  | |
| Lip and oral cavity cancer | 3.53 | 3.51 | 3.53 | 3.66 | 3.96 | 4.36 | 4.71 | 33.43 | |
| Colon and rectum cancer | 9.30 | 9.53 | 9.75 | 9.86 | 10.55 | 11.75 | 13.03 | 40.11 | |
| Stomach cancer | 8.29 | 8.12 | 8.19 | 8.47 | 8.68 | 8.77 | 8.95 | 7.96 | |
| Pancreatic cancer | 0.41 | 0.44 | 0.46 | 0.49 | 0.52 | 0.58 | 0.64 | 56.10 | |
| Non-Hodgkin's lymphoma | 0.00 | 0.00 | 0.00 | 0.00 | 0.00 | 0.00 | 0.00 |  | |
| Gallbladder and biliary tract cancer | 0.43 | 0.42 | 0.42 | 0.41 | 0.42 | 0.44 | 0.46 | 6.98 | |
| Kidney cancer | 1.97 | 2.07 | 2.27 | 2.26 | 2.29 | 2.37 | 2.62 | 32.99 | |
| Ovarian cancer | 2.47 | 2.76 | 3.19 | 3.59 | 3.93 | 4.49 | 4.92 | 99.19 | |
| Cervical cancer | 27.80 | 28.04 | 29.86 | 32.42 | 34.38 | 36.03 | 37.55 | 35.07 | |
| Breast cancer | 98.43 | 103.30 | 110.52 | 117.57 | 127.58 | 139.72 | 151.08 | 53.49 | |
| Oesophageal cancer | 1.17 | 1.15 | 1.13 | 1.14 | 1.15 | 1.20 | 1.25 | 6.84 | |
| Tracheal, bronchus and lung cancer | 8.50 | 8.33 | 8.41 | 8.60 | 9.12 | 9.82 | 10.50 | 23.53 | |
| Prostate cancer | 9.97 | 10.70 | 11.34 | 11.72 | 12.75 | 14.57 | 16.51 | 65.60 | |
| Thyroid cancer | 2.88 | 3.22 | 3.80 | 4.18 | 4.45 | 4.84 | 5.33 | 85.07 | |
| Leukaemia | 33.99 | 32.95 | 35.02 | 35.67 | 34.50 | 31.64 | 31.52 | -7.27 | |
| Multiple myeloma | 0.72 | 0.69 | 0.67 | 0.68 | 0.71 | 0.77 | 0.83 | 15.28 | |
| **Cardiovascular diseases (%)** |  |  |  |  |  |  |  |  | |
| Stroke | 0.93 | 0.92 | 0.91 | 0.92 | 0.95 | 0.99 | 1.02 | 8.96 | |
| Ischemic heart disease | 0.98 | 0.99 | 1.00 | 1.01 | 1.03 | 1.06 | 1.11 | 12.62 | |
| **Chronic respiratory diseases (%)** |  |  |  |  |  |  |  |  | |
| Chronic obstructive pulmonary disease | 1.49 | 1.48 | 1.46 | 1.46 | 1.48 | 1.53 | 1.59 | 6.88 | |
| Asthma | 5.33 | 5.29 | 5.27 | 5.04 | 4.51 | 4.27 | 4.18 | -21.51 | |
| **Digestive diseases (%)** |  |  |  |  |  |  |  |  | |
| Gallbladder and biliary diseases | 0.72 | 0.69 | 0.69 | 0.70 | 0.71 | 0.73 | 0.75 | 4.30 | |
| Upper digestive system diseases | 4.21 | 4.18 | 4.18 | 4.24 | 4.34 | 4.45 | 4.54 | 7.87 | |
| **Neurological disorders (%)** |  |  |  |  |  |  |  |  | |
| Alzheimer's disease and other dementias | 0.14 | 0.15 | 0.15 | 0.15 | 0.14 | 0.14 | 0.15 | 6.97 | |
| Parkinson's disease | 0.04 | 0.04 | 0.04 | 0.04 | 0.04 | 0.04 | 0.04 | 2.79 | |
| **Mental disorders (%)** |  |  |  |  |  |  |  |  | |
| Depressive disorders | 2.55 | 2.54 | 2.51 | 2.54 | 2.57 | 2.61 | 2.63 | 2.96 | |
| Anxiety disorder | 3.52 | 3.53 | 3.54 | 3.55 | 3.65 | 3.68 | 3.69 | 4.75 | |
| **Substance use disorders (%)** |  |  |  |  |  |  |  |  | |
| Alcohol use disorders | 0.63 | 0.63 | 0.64 | 0.65 | 0.67 | 0.68 | 0.69 | 9.47 | |
| Drug use disorders | 0.69 | 0.69 | 0.69 | 0.70 | 0.72 | 0.72 | 0.72 | 4.85 | |
| **Diabetes and kidney diseases (%)** |  |  |  |  |  |  |  |  | |
| Diabetes mellitus type 2 | 3.74 | 4.05 | 4.53 | 4.91 | 5.52 | 6.29 | 6.87 | 83.66 | |
| Chronic kidney disease | 5.11 | 5.19 | 5.28 | 5.41 | 5.62 | 5.87 | 6.13 | 19.94 | |
| **Musculoskeletal disorders (%)** |  |  |  |  |  |  |  |  | |
| Osteoarthritis | 2.17 | 2.13 | 2.12 | 2.16 | 2.24 | 2.38 | 2.53 | 16.49 | |
| **Fiji** |  |  |  |  |  |  |  |  | |
| **Neoplasms/Cancer (per 100,000 persons)** |  |  |  |  |  |  |  |  | |
| Lip and oral cavity cancer | 8.08 | 7.70 | 7.63 | 8.46 | 9.29 | 10.31 | 11.42 | 41.34 | |
| Colon and rectum cancer | 17.94 | 18.61 | 20.27 | 22.76 | 28.63 | 34.06 | 39.05 | 117.67 | |
| Stomach cancer | 5.43 | 5.22 | 5.23 | 5.33 | 6.35 | 6.87 | 7.24 | 33.33 | |
| Pancreatic cancer | 0.93 | 1.06 | 1.29 | 1.56 | 1.82 | 2.15 | 2.50 | 168.82 | |
| Non-Hodgkin's lymphoma | 0.00 | 0.00 | 0.01 | 0.01 | 0.02 | 0.02 | 0.49 |  | |
| Gallbladder and biliary tract cancer | 0.67 | 0.67 | 0.71 | 0.79 | 1.00 | 1.09 | 1.18 | 76.12 | |
| Kidney cancer | 3.45 | 3.72 | 4.19 | 4.09 | 4.60 | 5.15 | 6.04 | 75.07 | |
| Ovarian cancer | 2.98 | 3.74 | 4.58 | 5.01 | 5.22 | 5.43 | 5.96 | 100.00 | |
| Cervical cancer | 78.01 | 73.78 | 70.60 | 76.95 | 88.73 | 90.95 | 95.28 | 22.14 | |
| Breast cancer | 106.91 | 112.03 | 121.97 | 137.91 | 172.33 | 190.72 | 211.49 | 97.82 | |
| Oesophageal cancer | 1.56 | 1.56 | 1.63 | 1.89 | 2.46 | 2.75 | 2.97 | 90.38 | |
| Tracheal, bronchus and lung cancer | 5.04 | 5.07 | 5.43 | 5.80 | 6.63 | 7.53 | 8.27 | 64.09 | |
| Prostate cancer | 18.15 | 17.99 | 19.09 | 23.77 | 30.37 | 36.13 | 42.44 | 133.83 | |
| Thyroid cancer | 15.22 | 15.82 | 17.22 | 17.42 | 19.86 | 21.51 | 24.82 | 63.07 | |
| Leukaemia | 25.66 | 27.65 | 30.59 | 24.62 | 22.30 | 22.16 | 22.97 | -10.48 | |
| Multiple myeloma | 0.97 | 1.02 | 1.11 | 1.24 | 1.55 | 1.69 | 1.86 | 91.75 | |
| **Cardiovascular diseases (%)** |  |  |  |  |  |  |  |  | |
| Stroke | 1.01 | 1.04 | 1.15 | 1.19 | 1.29 | 1.34 | 1.45 | 43.37 | |
| Ischemic heart disease | 1.16 | 1.24 | 1.36 | 1.52 | 1.73 | 1.90 | 2.03 | 74.99 | |
| **Chronic respiratory diseases (%)** |  |  |  |  |  |  |  |  | |
| Chronic obstructive pulmonary disease | 0.51 | 0.48 | 0.47 | 0.48 | 0.47 | 0.48 | 0.52 | 2.22 | |
| Asthma | 4.14 | 4.03 | 3.87 | 3.63 | 3.20 | 3.02 | 3.20 | -22.65 | |
| **Digestive diseases (%)** |  |  |  |  |  |  |  |  | |
| Gallbladder and biliary diseases | 0.70 | 0.71 | 0.74 | 0.80 | 0.83 | 0.88 | 0.91 | 29.70 | |
| Upper digestive system diseases | 4.48 | 4.64 | 4.86 | 5.10 | 5.27 | 5.37 | 5.46 | 21.74 | |
| **Neurological disorders (%)** |  |  |  |  |  |  |  |  | |
| Alzheimer's disease and other dementias | 0.16 | 0.17 | 0.18 | 0.20 | 0.23 | 0.27 | 0.30 | 90.70 | |
| Parkinson's disease | 0.05 | 0.05 | 0.06 | 0.07 | 0.08 | 0.08 | 0.09 | 71.44 | |
| **Mental disorders (%)** |  |  |  |  |  |  |  |  | |
| Depressive disorders | 2.45 | 2.55 | 2.68 | 2.79 | 2.83 | 2.85 | 2.89 | 17.96 | |
| Anxiety disorder | 3.69 | 3.78 | 3.86 | 3.90 | 3.95 | 3.99 | 4.02 | 8.95 | |
| **Substance use disorders (%)** |  |  |  |  |  |  |  |  | |
| Alcohol use disorders | 0.54 | 0.55 | 0.57 | 0.60 | 0.61 | 0.58 | 0.60 | 12.47 | |
| Drug use disorders | 0.74 | 0.75 | 0.76 | 0.77 | 0.75 | 0.72 | 0.70 | -5.27 | |
| **Diabetes and kidney diseases (%)** |  |  |  |  |  |  |  |  | |
| Diabetes mellitus type 2 | 5.35 | 6.78 | 8.31 | 10.39 | 12.26 | 13.48 | 14.64 | 173.57 | |
| Chronic kidney disease | 6.96 | 7.49 | 8.07 | 8.67 | 9.32 | 9.85 | 10.31 | 48.20 | |
| **Musculoskeletal disorders (%)** |  |  |  |  |  |  |  |  | |
| Osteoarthritis | 2.55 | 2.86 | 3.27 | 3.73 | 4.13 | 4.51 | 4.82 | 88.86 | |
| **Philippines** |  |  |  |  |  |  |  |  | |
| **Neoplasms/Cancer (per 100,000 persons)** |  |  |  |  |  |  |  |  | |
| Lip and oral cavity cancer | 8.45 | 8.97 | 8.04 | 7.28 | 7.39 | 8.03 | 8.85 | 4.73 | |
| Colon and rectum cancer | 27.75 | 30.90 | 31.29 | 32.50 | 37.95 | 47.45 | 54.08 | 94.88 | |
| Stomach cancer | 4.64 | 4.67 | 4.13 | 3.90 | 3.95 | 4.13 | 4.22 | -9.05 | |
| Pancreatic cancer | 1.20 | 1.39 | 1.42 | 1.44 | 1.56 | 2.02 | 2.29 | 90.83 | |
| Non-Hodgkin's lymphoma | 1.51 | 1.24 | 1.16 | 1.08 | 1.25 | 1.60 | 2.13 | 41.06 | |
| Gallbladder and biliary tract cancer | 0.46 | 0.48 | 0.45 | 0.45 | 0.48 | 0.53 | 0.56 | 21.74 | |
| Kidney cancer | 7.19 | 7.32 | 7.67 | 7.63 | 8.11 | 9.97 | 10.99 | 52.85 | |
| Ovarian cancer | 11.19 | 12.96 | 13.70 | 14.54 | 16.33 | 18.99 | 20.73 | 85.25 | |
| Cervical cancer | 27.68 | 28.37 | 27.02 | 27.08 | 29.24 | 31.66 | 32.28 | 16.62 | |
| Breast cancer | 79.27 | 87.05 | 89.73 | 93.48 | 105.10 | 126.78 | 138.21 | 74.35 | |
| Oesophageal cancer | 1.14 | 1.22 | 1.16 | 1.15 | 1.22 | 1.36 | 1.43 | 25.44 | |
| Tracheal, bronchus and lung cancer | 11.40 | 12.75 | 12.13 | 10.99 | 10.92 | 11.86 | 13.03 | 14.30 | |
| Prostate cancer | 16.71 | 20.08 | 20.38 | 20.47 | 23.18 | 27.88 | 32.16 | 92.46 | |
| Thyroid cancer | 17.89 | 19.83 | 19.63 | 19.39 | 21.04 | 24.37 | 26.55 | 48.41 | |
| Leukaemia | 46.62 | 38.75 | 36.27 | 33.68 | 32.60 | 31.74 | 29.71 | -36.27 | |
| Multiple myeloma | 0.68 | 0.74 | 0.71 | 0.72 | 0.82 | 0.97 | 1.05 | 54.41 | |
| **Cardiovascular diseases (%)** |  |  |  |  |  |  |  |  | |
| Stroke | 0.84 | 0.84 | 0.87 | 0.93 | 1.01 | 1.07 | 1.13 | 34.71 | |
| Ischemic heart disease | 1.06 | 1.14 | 1.31 | 1.36 | 1.39 | 1.50 | 1.61 | 51.86 | |
| **Chronic respiratory diseases (%)** |  |  |  |  |  |  |  |  | |
| Chronic obstructive pulmonary disease | 1.11 | 1.21 | 1.30 | 1.39 | 1.53 | 1.64 | 1.72 | 54.81 | |
| Asthma | 6.24 | 5.92 | 5.45 | 5.21 | 4.90 | 4.73 | 4.65 | -25.45 | |
| **Digestive diseases (%)** |  |  |  |  |  |  |  |  | |
| Gallbladder and biliary diseases | 1.08 | 1.08 | 1.07 | 1.09 | 1.32 | 1.38 | 1.41 | 31.03 | |
| Upper digestive system diseases | 4.47 | 4.57 | 4.67 | 4.79 | 5.01 | 5.19 | 5.35 | 19.67 | |
| **Neurological disorders (%)** |  |  |  |  |  |  |  |  | |
| Alzheimer's disease and other dementias | 0.18 | 0.19 | 0.21 | 0.22 | 0.24 | 0.26 | 0.29 | 60.13 | |
| Parkinson's disease | 0.03 | 0.03 | 0.04 | 0.04 | 0.05 | 0.05 | 0.06 | 68.80 | |
| **Mental disorders (%)** |  |  |  |  |  |  |  |  | |
| Depressive disorders | 2.42 | 2.39 | 2.29 | 2.28 | 2.39 | 2.50 | 2.57 | 6.32 | |
| Anxiety disorder | 3.92 | 3.99 | 4.06 | 4.12 | 4.25 | 4.33 | 4.40 | 12.33 | |
| **Substance use disorders (%)** |  |  |  |  |  |  |  |  | |
| Alcohol use disorders | 0.85 | 0.82 | 0.75 | 0.69 | 0.72 | 0.73 | 0.76 | -10.52 | |
| Drug use disorders | 0.60 | 0.58 | 0.56 | 0.54 | 0.55 | 0.55 | 0.56 | -5.30 | |
| **Diabetes and kidney diseases (%)** |  |  |  |  |  |  |  |  | |
| Diabetes mellitus type 2 | 1.90 | 2.10 | 2.25 | 2.48 | 2.49 | 2.76 | 3.26 | 71.19 | |
| Chronic kidney disease | 6.18 | 6.58 | 6.99 | 7.40 | 7.98 | 8.56 | 9.13 | 47.79 | |
| **Musculoskeletal disorders (%)** |  |  |  |  |  |  |  |  | |
| Osteoarthritis | 1.74 | 1.83 | 2.01 | 2.18 | 2.40 | 2.65 | 2.86 | 64.63 | |
| **Guam (USA)** |  |  |  |  |  |  |  |  | |
| **Neoplasms/Cancer (per 100,000 persons)** |  |  |  |  |  |  |  |  | |
| Lip and oral cavity cancer | 6.70 | 7.94 | 8.52 | 10.09 | 11.57 | 13.79 | 14.29 | 113.28 |  |
| Colon and rectum cancer | 51.51 | 56.96 | 70.11 | 69.53 | 79.08 | 92.96 | 102.67 | 99.32 |  |
| Stomach cancer | 6.59 | 7.45 | 8.66 | 7.76 | 8.60 | 9.66 | 10.19 | 54.63 |  |
| Pancreatic cancer | 1.67 | 1.76 | 2.37 | 3.02 | 3.51 | 4.11 | 4.50 | 169.46 |  |
| Non-Hodgkin's lymphoma | 7.96 | 12.07 | 10.48 | 8.22 | 9.57 | 11.85 | 12.81 | 60.93 |  |
| Gallbladder and biliary tract cancer | 0.72 | 0.77 | 0.81 | 0.72 | 9.57 | 0.66 | 0.74 | 2.78 |  |
| Kidney cancer | 14.25 | 16.63 | 18.22 | 22.15 | 20.54 | 17.40 | 17.90 | 25.61 |  |
| Ovarian cancer | 6.52 | 7.96 | 12.61 | 15.01 | 15.54 | 16.51 | 16.38 | 151.23 |  |
| Cervical cancer | 28.57 | 37.04 | 36.18 | 35.61 | 37.17 | 39.39 | 39.46 | 38.12 |  |
| Breast cancer | 85.79 | 99.82 | 131.10 | 138.39 | 156.88 | 178.44 | 187.76 | 118.86 |  |
| Oesophageal cancer | 2.10 | 2.20 | 2.24 | 2.85 | 3.48 | 4.29 | 4.47 | 112.86 |  |
| Tracheal, bronchus and lung cancer | 21.74 | 22.58 | 23.70 | 25.91 | 29.76 | 34.07 | 36.93 | 69.87 |  |
| Prostate cancer | 41.47 | 51.37 | 61.03 | 66.96 | 74.21 | 83.22 | 93.19 | 124.72 |  |
| Thyroid cancer | 7.30 | 9.02 | 17.24 | 22.58 | 22.48 | 25.45 | 26.73 | 266.16 |  |
| Leukaemia | 17.79 | 18.85 | 19.72 | 19.30 | 20.13 | 20.99 | 22.09 | 24.17 |  |
| Multiple myeloma | 1.95 | 2.19 | 2.37 | 2.26 | 2.62 | 2.73 | 3.11 | 59.49 |  |
| **Cardiovascular diseases (%)** |  |  |  |  |  |  |  |  |  |
| Stroke | 1.09 | 1.18 | 1.34 | 1.49 | 1.69 | 1.84 | 1.95 | 79.85 |  |
| Ischemic heart disease | 1.13 | 1.35 | 1.63 | 1.93 | 2.26 | 2.58 | 2.78 | 145.15 |  |
| **Chronic respiratory diseases (%)** |  |  |  |  |  |  |  |  |  |
| Chronic obstructive pulmonary disease | 0.72 | 0.79 | 0.90 | 0.95 | 0.97 | 1.01 | 1.07 | 48.73 |  |
| Asthma | 3.85 | 3.39 | 3.43 | 3.25 | 3.02 | 2.92 | 3.33 | -13.53 |  |
| **Digestive diseases (%)** |  |  |  |  |  |  |  |  |  |
| Gallbladder and biliary diseases | 0.61 | 0.60 | 0.64 | 0.67 | 0.70 | 0.73 | 0.77 | 26.97 |  |
| Upper digestive system diseases | 4.95 | 5.07 | 5.22 | 5.42 | 5.58 | 5.73 | 5.85 | 18.15 |  |
| **Neurological disorders (%)** |  |  |  |  |  |  |  |  |  |
| Alzheimer's disease and other dementias | 0.19 | 0.23 | 0.29 | 0.36 | 0.46 | 0.56 | 0.64 | 234.87 |  |
| Parkinson's disease | 0.07 | 0.07 | 0.09 | 0.10 | 0.11 | 0.12 | 0.13 | 105.48 |  |
| **Mental disorders (%)** |  |  |  |  |  |  |  |  |  |
| Depressive disorders | 3.01 | 3.01 | 3.01 | 3.11 | 3.23 | 3.29 | 3.32 | 10.06 |  |
| Anxiety disorder | 3.76 | 3.76 | 3.85 | 3.92 | 4.00 | 4.05 | 4.07 | 8.25 |  |
| **Substance use disorders (%)** |  |  |  |  |  |  |  |  |  |
| Alcohol use disorders | 0.75 | 0.75 | 0.75 | 0.77 | 0.78 | 0.79 | 0.79 | 5.33 |  |
| Drug use disorders | 0.87 | 0.82 | 0.78 | 0.77 | 0.76 | 0.75 | 0.74 | -14.91 |  |
| **Diabetes and kidney diseases (%)** |  |  |  |  |  |  |  |  |  |
| Diabetes mellitus type 2 | 3.70 | 4.12 | 4.78 | 5.60 | 6.55 | 7.62 | 8.73 | 135.95 |  |
| Chronic kidney disease | 7.32 | 8.03 | 8.96 | 9.76 | 10.41 | 11.07 | 11.72 | 60.06 |  |
| **Musculoskeletal disorders (%)** |  |  |  |  |  |  |  |  |  |
| Osteoarthritis | 3.15 | 3.54 | 4.14 | 4.81 | 5.46 | 6.06 | 6.46 | 104.78 |  |
| **Samoa** |  |  |  |  |  |  |  |  |  |
| **Neoplasms/Cancer (per 100,000 persons)** |  |  |  |  |  |  |  |  |  |
| Lip and oral cavity cancer | 5.18 | 5.39 | 5.62 | 5.85 | 6.07 | 6.01 | 6.52 | 25.87 |  |
| Colon and rectum cancer | 22.38 | 24.62 | 27.58 | 29.68 | 31.32 | 31.05 | 34.69 | 55.00 |  |
| Stomach cancer | 10.75 | 11.09 | 11.23 | 11.31 | 11.50 | 11.25 | 11.43 | 6.33 |  |
| Pancreatic cancer | 1.67 | 1.92 | 2.15 | 2.26 | 2.08 | 2.06 | 2.27 | 35.93 |  |
| Non-Hodgkin's lymphoma | 0.02 | 0.02 | 0.03 | 0.03 | 0.10 | 0.09 | 0.40 | 1900.00 |  |
| Gallbladder and biliary tract cancer | 0.77 | 0.82 | 0.86 | 0.89 | 0.91 | 0.88 | 0.90 | 16.88 |  |
| Kidney cancer | 4.83 | 5.02 | 5.65 | 5.68 | 5.42 | 5.20 | 6.00 | 24.22 |  |
| Ovarian cancer | 9.73 | 12.53 | 16.35 | 19.15 | 19.89 | 20.34 | 21.88 | 124.87 |  |
| Cervical cancer | 39.14 | 43.56 | 48.45 | 49.96 | 53.77 | 51.71 | 53.54 | 36.79 |  |
| Breast cancer | 77.43 | 86.25 | 101.31 | 113.71 | 126.35 | 128.00 | 139.10 | 79.65 |  |
| Oesophageal cancer | 1.52 | 1.61 | 1.64 | 1.65 | 1.60 | 1.57 | 1.63 | 7.24 |  |
| Tracheal, bronchus and lung cancer | 6.19 | 6.46 | 6.84 | 7.23 | 7.55 | 7.68 | 8.09 | 30.69 |  |
| Prostate cancer | 22.30 | 23.66 | 25.57 | 26.92 | 27.95 | 27.86 | 32.05 | 43.72 |  |
| Thyroid cancer | 11.94 | 14.50 | 18.38 | 19.90 | 20.14 | 18.93 | 21.29 | 78.31 |  |
| Leukaemia | 22.81 | 21.33 | 20.94 | 20.53 | 20.51 | 19.62 | 19.78 | -13.28 |  |
| Multiple myeloma | 1.15 | 1.25 | 1.33 | 1.37 | 1.37 | 1.35 | 1.46 | 26.96 |  |
| **Cardiovascular diseases (%)** |  |  |  |  |  |  |  |  |  |
| Stroke | 1.16 | 1.21 | 1.29 | 1.32 | 1.35 | 1.33 | 1.36 | 17.19 |  |
| Ischemic heart disease | 1.29 | 1.40 | 1.53 | 1.63 | 1.67 | 1.67 | 1.69 | 30.95 |  |
| **Chronic respiratory diseases (%)** |  |  |  |  |  |  |  |  |  |
| Chronic obstructive pulmonary disease | 1.00 | 1.01 | 1.03 | 1.01 | 0.94 | 0.88 | 0.90 | -10.48 |  |
| Asthma | 3.35 | 3.12 | 3.00 | 2.90 | 2.76 | 2.67 | 2.95 | -11.76 |  |
| **Digestive diseases (%)** |  |  |  |  |  |  |  |  |  |
| Gallbladder and biliary diseases | 0.73 | 0.74 | 0.77 | 0.81 | 0.81 | 0.82 | 0.83 | 12.90 |  |
| Upper digestive system diseases | 4.14 | 4.33 | 4.51 | 4.63 | 4.68 | 4.69 | 4.76 | 14.97 |  |
| **Neurological disorders (%)** |  |  |  |  |  |  |  |  |  |
| Alzheimer's disease and other dementias | 0.23 | 0.25 | 0.27 | 0.30 | 0.32 | 0.32 | 0.32 | 39.51 |  |
| Parkinson's disease | 0.06 | 0.07 | 0.07 | 0.08 | 0.08 | 0.08 | 0.08 | 28.31 |  |
| **Mental disorders (%)** |  |  |  |  |  |  |  |  |  |
| Depressive disorders | 2.37 | 2.42 | 2.44 | 2.46 | 2.43 | 2.45 | 2.49 | 4.92 |  |
| Anxiety disorder | 3.65 | 3.70 | 3.74 | 3.76 | 3.82 | 3.89 | 3.93 | 7.89 |  |
| **Substance use disorders (%)** |  |  |  |  |  |  |  |  |  |
| Alcohol use disorders | 0.51 | 0.54 | 0.56 | 0.57 | 0.57 | 0.55 | 0.57 | 10.10 |  |
| Drug use disorders | 0.88 | 1.03 | 1.06 | 1.03 | 1.02 | 1.02 | 1.05 | 19.62 |  |
| **Diabetes and kidney diseases (%)** |  |  |  |  |  |  |  |  |  |
| Diabetes mellitus type 2 | 3.91 | 4.69 | 5.78 | 6.86 | 7.45 | 8.01 | 8.66 | 121.52 |  |
| Chronic kidney disease | 6.31 | 6.89 | 7.58 | 8.08 | 8.36 | 8.38 | 8.64 | 36.91 |  |
| **Musculoskeletal disorders (%)** |  |  |  |  |  |  |  |  |  |
| Osteoarthritis | 2.79 | 3.01 | 3.28 | 3.59 | 3.72 | 3.81 | 3.91 | 40.07 |  |
| **Japan** |  |  |  |  |  |  |  |  |  |
| **Neoplasms/Cancer (per 100,000 persons)** |  |  |  |  |  |  |  |  |  |
| Lip and oral cavity cancer | 8.54 | 10.58 | 12.57 | 14.69 | 17.06 | 17.29 | 17.02 | 99.30 |  |
| Colon and rectum cancer | 334.15 | 431.98 | 493.02 | 573.40 | 646.61 | 717.00 | 749.85 | 124.41 |  |
| Stomach cancer | 253.85 | 256.91 | 245.97 | 248.80 | 244.93 | 230.21 | 226.85 | -10.64 |  |
| Pancreatic cancer | 12.04 | 14.25 | 16.62 | 21.57 | 26.94 | 30.91 | 32.70 | 171.59 |  |
| Non-Hodgkin's lymphoma | 37.13 | 51.23 | 68.37 | 85.55 | 101.55 | 116.83 | 123.77 | 233.34 |  |
| Gallbladder and biliary tract cancer | 15.62 | 17.45 | 18.84 | 21.62 | 24.15 | 25.32 | 26.50 | 69.65 |  |
| Kidney cancer | 18.04 | 23.86 | 29.66 | 37.47 | 42.20 | 45.10 | 45.70 | 153.33 |  |
| Ovarian cancer | 20.60 | 24.88 | 27.66 | 30.47 | 31.95 | 31.84 | 32.00 | 55.34 |  |
| Cervical cancer | 43.65 | 40.43 | 41.94 | 48.03 | 55.11 | 56.59 | 53.81 | 23.28 |  |
| Breast cancer | 312.07 | 360.63 | 428.69 | 517.22 | 622.12 | 679.79 | 694.49 | 122.54 |  |
| Oesophageal cancer | 22.83 | 29.67 | 37.73 | 47.63 | 52.01 | 52.12 | 52.85 | 131.49 |  |
| Tracheal, bronchus and lung cancer | 71.95 | 89.22 | 109.04 | 147.45 | 184.42 | 197.18 | 198.23 | 175.51 |  |
| Prostate cancer | 78.66 | 102.70 | 152.77 | 247.80 | 311.72 | 340.35 | 358.57 | 355.85 |  |
| Thyroid cancer | 37.47 | 43.66 | 49.31 | 58.50 | 64.06 | 61.64 | 60.08 | 60.34 |  |
| Leukaemia | 48.17 | 58.98 | 71.85 | 84.68 | 90.09 | 91.76 | 91.69 | 90.35 |  |
| Multiple myeloma | 6.65 | 8.05 | 10.00 | 13.17 | 15.93 | 16.68 | 17.49 | 163.01 |  |
| **Cardiovascular diseases (%)** |  |  |  |  |  |  |  |  |  |
| Stroke | 1.85 | 2.05 | 2.34 | 2.51 | 2.76 | 2.99 | 3.15 | 70.75 |  |
| Ischemic heart disease | 1.71 | 1.88 | 2.06 | 2.21 | 2.39 | 2.66 | 2.89 | 69.60 |  |
| **Chronic respiratory diseases (%)** |  |  |  |  |  |  |  |  |  |
| Chronic obstructive pulmonary disease | 2.38 | 2.68 | 2.86 | 3.07 | 3.14 | 3.50 | 3.75 | 57.45 |  |
| Asthma | 8.93 | 8.50 | 6.68 | 4.93 | 4.13 | 3.74 | 4.00 | -55.18 |  |
| **Digestive diseases (%)** |  |  |  |  |  |  |  |  |  |
| Gallbladder and biliary diseases | 4.63 | 4.77 | 4.93 | 5.09 | 5.34 | 5.64 | 6.05 | 30.81 |  |
| Upper digestive system diseases | 7.18 | 7.51 | 7.65 | 8.00 | 8.47 | 8.65 | 8.82 | 22.78 |  |
| **Neurological disorders (%)** |  |  |  |  |  |  |  |  |  |
| Alzheimer's disease and other dementias | 0.77 | 0.96 | 1.27 | 1.64 | 2.02 | 2.66 | 3.08 | 299.06 |  |
| Parkinson's disease | 0.08 | 0.10 | 0.11 | 0.15 | 0.17 | 0.18 | 0.23 | 183.07 |  |
| **Mental disorders (%)** |  |  |  |  |  |  |  |  |  |
| Depressive disorders | 2.34 | 2.28 | 2.58 | 2.70 | 2.58 | 2.52 | 2.55 | 9.09 |  |
| Anxiety disorder | 2.85 | 2.85 | 2.84 | 2.81 | 2.62 | 2.46 | 2.44 | -14.47 |  |
| **Substance use disorders (%)** |  |  |  |  |  |  |  | -2.93 |  |
| Alcohol use disorders | 0.67 | 0.66 | 0.65 | 0.63 | 0.62 | 0.63 | 0.65 | -2.93 |  |
| Drug use disorders | 0.75 | 0.75 | 0.74 | 0.70 | 0.66 | 0.63 | 0.62 | -17.46 |  |
| **Diabetes and kidney diseases (%)** |  |  |  |  |  |  |  |  |  |
| Diabetes mellitus type 2 | 3.84 | 4.38 | 4.92 | 4.78 | 5.17 | 5.92 | 6.86 | 78.61 |  |
| Chronic kidney disease | 11.02 | 12.15 | 13.25 | 14.95 | 16.44 | 17.78 | 19.33 | 75.43 |  |
| **Musculoskeletal disorders (%)** |  |  |  |  |  |  |  |  |  |
| Osteoarthritis | 10.36 | 11.79 | 13.22 | 14.82 | 17.18 | 18.30 | 18.04 | 74.22 |  |
| **Singapore** |  |  |  |  |  |  |  |  |  |
| **Neoplasms/Cancer (per 100,000 persons)** |  |  |  |  |  |  |  |  |  |
| Lip and oral cavity cancer | 7.28 | 7.17 | 6.75 | 7.57 | 7.57 | 7.49 | 8.00 | 9.89 |  |
| Colon and rectum cancer | 158.57 | 186.44 | 196.64 | 226.47 | 259.92 | 296.82 | 342.54 | 116.02 |  |
| Stomach cancer | 41.51 | 41.78 | 38.47 | 39.11 | 42.11 | 42.74 | 45.56 | 9.76 |  |
| Pancreatic cancer | 3.20 | 3.49 | 3.79 | 4.78 | 5.83 | 7.04 | 8.05 | 151.56 |  |
| Non-Hodgkin's lymphoma | 15.07 | 22.66 | 25.13 | 29.74 | 37.77 | 44.39 | 49.02 | 225.28 |  |
| Gallbladder and biliary tract cancer | 1.54 | 1.79 | 1.87 | 2.15 | 2.17 | 2.27 | 2.58 | 67.53 |  |
| Kidney cancer | 10.02 | 13.02 | 15.58 | 20.12 | 25.64 | 29.80 | 32.43 | 223.65 |  |
| Ovarian cancer | 15.16 | 16.85 | 17.99 | 22.16 | 22.54 | 22.79 | 24.67 | 62.73 |  |
| Cervical cancer | 57.28 | 52.55 | 42.45 | 37.10 | 33.29 | 33.69 | 34.69 | -39.44 |  |
| Breast cancer | 222.98 | 252.60 | 283.78 | 343.41 | 399.59 | 440.98 | 479.66 | 115.11 |  |
| Oesophageal cancer | 8.08 | 7.60 | 7.06 | 7.31 | 8.67 | 9.83 | 11.56 | 43.07 |  |
| Tracheal, bronchus and lung cancer | 40.29 | 43.48 | 45.24 | 53.82 | 63.91 | 73.78 | 85.01 | 111.00 |  |
| Prostate cancer | 30.25 | 40.32 | 52.09 | 70.67 | 89.96 | 116.75 | 144.63 | 378.12 |  |
| Thyroid cancer | 28.05 | 30.28 | 35.05 | 42.80 | 43.64 | 41.85 | 44.77 | 59.61 |  |
| Leukaemia | 20.87 | 22.28 | 22.88 | 29.58 | 43.86 | 52.09 | 55.11 | 164.06 |  |
| Multiple myeloma | 2.33 | 2.61 | 2.81 | 3.48 | 4.14 | 5.03 | 5.98 | 156.65 |  |
| **Cardiovascular diseases (%)** |  |  |  |  |  |  |  |  |  |
| Stroke | 1.45 | 1.39 | 1.30 | 1.27 | 1.23 | 1.32 | 1.46 | 0.27 |  |
| Ischemic heart disease | 1.19 | 1.19 | 1.19 | 1.30 | 1.40 | 1.63 | 1.85 | 55.07 |  |
| **Chronic respiratory diseases (%)** |  |  |  |  |  |  |  |  |  |
| Chronic obstructive pulmonary disease | 2.01 | 2.12 | 2.01 | 1.94 | 1.74 | 1.93 | 2.00 | -0.27 |  |
| Asthma | 6.20 | 5.76 | 4.70 | 3.85 | 3.51 | 3.59 | 3.52 | -43.24 |  |
| **Digestive diseases (%)** |  |  |  |  |  |  |  |  |  |
| Gallbladder and biliary diseases | 3.64 | 3.63 | 3.68 | 3.75 | 3.81 | 4.02 | 4.32 | 18.66 |  |
| Upper digestive system diseases | 7.45 | 7.61 | 7.95 | 8.29 | 8.69 | 9.19 | 9.56 | 28.29 |  |
| **Neurological disorders (%)** |  |  |  |  |  |  |  |  |  |
| Alzheimer's disease and other dementias | 0.30 | 0.33 | 0.35 | 0.43 | 0.48 | 0.60 | 0.79 | 163.28 |  |
| Parkinson's disease | 0.04 | 0.05 | 0.05 | 0.06 | 0.07 | 0.08 | 0.10 | 126.18 |  |
| **Mental disorders (%)** |  |  |  |  |  |  |  |  |  |
| Depressive disorders | 3.06 | 3.02 | 2.91 | 2.36 | 2.31 | 2.25 | 2.32 | -24.42 |  |
| Anxiety disorder | 2.98 | 2.96 | 2.98 | 3.04 | 3.00 | 2.92 | 2.89 | -2.99 |  |
| **Substance use disorders (%)** |  |  |  |  |  |  |  |  |  |
| Alcohol use disorders | 0.75 | 0.75 | 0.78 | 0.72 | 0.76 | 0.75 | 0.79 | 5.19 |  |
| Drug use disorders | 0.95 | 0.94 | 0.95 | 0.91 | 0.95 | 0.86 | 0.77 | -18.56 |  |
| **Diabetes and kidney diseases (%)** |  |  |  |  |  |  |  |  |  |
| Diabetes mellitus type 2 | 4.32 | 5.00 | 5.29 | 5.85 | 6.36 | 7.56 | 7.71 | 78.27 |  |
| Chronic kidney disease | 8.41 | 8.76 | 9.40 | 10.09 | 10.71 | 11.97 | 13.11 | 55.89 |  |
| **Musculoskeletal disorders (%)** |  |  |  |  |  |  |  |  |  |
| Osteoarthritis | 6.49 | 6.87 | 7.35 | 8.44 | 9.38 | 10.88 | 12.29 | 89.21 |  |
| **Kiribati** |  |  |  |  |  |  |  |  |  |
| **Neoplasms/Cancer (per 100,000 persons)** |  |  |  |  |  |  |  |  |  |
| Lip and oral cavity cancer | 29.49 | 27.08 | 28.75 | 30.06 | 34.92 | 36.47 | 39.90 | 35.30 |  |
| Colon and rectum cancer | 18.56 | 18.53 | 19.42 | 19.07 | 19.70 | 20.06 | 22.37 | 20.53 |  |
| Stomach cancer | 13.77 | 13.54 | 13.94 | 13.19 | 13.50 | 13.44 | 13.59 | -1.31 |  |
| Pancreatic cancer | 0.82 | 0.93 | 1.03 | 1.11 | 1.00 | 1.01 | 1.16 | 41.46 |  |
| Non-Hodgkin's lymphoma | 0.00 | 0.00 | 0.01 | 0.01 | 0.01 | 0.01 | 0.01 |  |  |
| Gallbladder and biliary tract cancer | 0.84 | 0.79 | 0.80 | 0.78 | 0.82 | 0.84 | 0.88 | 4.76 |  |
| Kidney cancer | 7.70 | 8.50 | 9.84 | 10.46 | 10.13 | 9.51 | 10.87 | 41.17 |  |
| Ovarian cancer | 2.95 | 3.62 | 4.47 | 5.15 | 5.36 | 5.62 | 6.22 | 110.85 |  |
| Cervical cancer | 179.21 | 180.81 | 186.90 | 175.23 | 188.92 | 183.28 | 191.78 | 7.01 |  |
| Breast cancer | 88.78 | 89.77 | 99.40 | 102.33 | 111.40 | 114.68 | 126.13 | 42.07 |  |
| Oesophageal cancer | 5.88 | 5.94 | 6.25 | 6.40 | 6.24 | 6.21 | 6.55 | 11.39 |  |
| Tracheal, bronchus and lung cancer | 10.79 | 10.52 | 10.77 | 10.61 | 11.17 | 11.65 | 12.45 | 15.38 |  |
| Prostate cancer | 10.36 | 9.66 | 9.18 | 8.77 | 9.82 | 10.21 | 11.91 | 14.96 |  |
| Thyroid cancer | 1.76 | 1.95 | 2.27 | 2.30 | 2.42 | 2.32 | 2.60 | 47.73 |  |
| Leukaemia | 32.35 | 31.61 | 32.56 | 29.65 | 29.23 | 26.25 | 26.15 | -19.17 |  |
| Multiple myeloma | 0.89 | 0.88 | 0.89 | 0.88 | 0.89 | 0.91 | 0.98 | 10.11 |  |
| **Cardiovascular diseases (%)** |  |  |  |  |  |  |  |  |  |
| Stroke | 1.56 | 1.55 | 1.54 | 1.54 | 1.52 | 1.57 | 1.57 | 0.98 |  |
| Ischemic heart disease | 1.07 | 1.08 | 1.11 | 1.14 | 1.19 | 1.24 | 1.30 | 21.15 |  |
| **Chronic respiratory diseases (%)** |  |  |  |  |  |  |  |  |  |
| Chronic obstructive pulmonary disease | 0.63 | 0.61 | 0.62 | 0.63 | 0.64 | 0.64 | 0.67 | 7.29 |  |
| Asthma | 6.18 | 5.96 | 5.71 | 5.31 | 4.86 | 4.47 | 4.45 | -27.96 |  |
| **Digestive diseases (%)** |  |  |  |  |  |  |  |  |  |
| Gallbladder and biliary diseases | 1.08 | 1.07 | 1.08 | 1.07 | 1.08 | 1.12 | 1.13 | 4.63 |  |
| Upper digestive system diseases | 4.60 | 4.57 | 4.60 | 4.64 | 4.77 | 4.87 | 4.94 | 7.31 |  |
| **Neurological disorders (%)** |  |  |  |  |  |  |  |  |  |
| Alzheimer's disease and other dementias | 0.15 | 0.15 | 0.15 | 0.15 | 0.16 | 0.16 | 0.17 | 12.32 |  |
| Parkinson's disease | 0.05 | 0.05 | 0.05 | 0.05 | 0.05 | 0.05 | 0.05 | 6.29 |  |
| **Mental disorders (%)** |  |  |  |  |  |  |  |  |  |
| Depressive disorders | 2.57 | 2.50 | 2.47 | 2.50 | 2.56 | 2.60 | 2.62 | 2.11 |  |
| Anxiety disorder | 3.59 | 3.61 | 3.67 | 3.71 | 3.80 | 3.85 | 3.88 | 8.10 |  |
| **Substance use disorders (%)** |  |  |  |  |  |  |  |  |  |
| Alcohol use disorders | 0.52 | 0.51 | 0.51 | 0.51 | 0.53 | 0.53 | 0.54 | 5.57 |  |
| Drug use disorders | 0.68 | 0.65 | 0.67 | 0.71 | 0.74 | 0.72 | 0.71 | 4.11 |  |
| **Diabetes and kidney diseases (%)** |  |  |  |  |  |  |  |  |  |
| Diabetes mellitus type 2 | 4.77 | 5.42 | 6.42 | 7.31 | 8.07 | 8.92 | 9.64 | 102.12 |  |
| Chronic kidney disease | 6.31 | 6.44 | 6.71 | 6.86 | 7.18 | 7.48 | 7.84 | 24.11 |  |
| **Musculoskeletal disorders (%)** |  |  |  |  |  |  |  |  |  |
| Osteoarthritis | 2.52 | 2.56 | 2.71 | 2.88 | 2.96 | 3.13 | 3.29 | 30.57 |  |
| **Solomon Islands** |  |  |  |  |  |  |  |  |  |
| **Neoplasms/Cancer (per 100,000 persons)** |  |  |  |  |  |  |  |  |  |
| Lip and oral cavity cancer | 5.49 | 5.77 | 6.31 | 6.53 | 7.10 | 8.36 | 8.99 | 63.75 |  |
| Colon and rectum cancer | 14.38 | 15.90 | 17.92 | 17.74 | 18.58 | 22.35 | 24.53 | 70.58 |  |
| Stomach cancer | 15.96 | 16.28 | 17.20 | 17.78 | 17.90 | 18.85 | 18.95 | 18.73 |  |
| Pancreatic cancer | 0.61 | 0.73 | 0.88 | 1.02 | 0.96 | 1.10 | 1.20 | 96.72 |  |
| Non-Hodgkin's lymphoma | 2.77 | 3.11 | 3.65 | 3.10 | 3.08 | 4.17 | 5.67 | 104.69 |  |
| Gallbladder and biliary tract cancer | 0.75 | 0.78 | 0.84 | 0.84 | 0.82 | 0.90 | 0.92 | 22.67 |  |
| Kidney cancer | 2.60 | 2.96 | 3.70 | 3.56 | 3.54 | 4.20 | 4.55 | 75.00 |  |
| Ovarian cancer | 2.95 | 3.67 | 4.89 | 6.02 | 6.67 | 8.60 | 9.51 | 222.37 |  |
| Cervical cancer | 69.74 | 73.23 | 82.65 | 86.87 | 94.60 | 107.90 | 110.66 | 58.68 |  |
| Breast cancer | 42.93 | 44.43 | 59.19 | 112.39 | 141.35 | 178.44 | 310.99 | 624.41 |  |
| Oesophageal cancer | 2.20 | 2.29 | 2.45 | 2.54 | 2.37 | 2.51 | 2.57 | 16.82 |  |
| Tracheal, bronchus and lung cancer | 12.96 | 13.55 | 14.50 | 14.51 | 14.73 | 16.42 | 17.16 | 32.41 |  |
| Prostate cancer | 16.36 | 17.87 | 20.66 | 20.19 | 20.62 | 24.38 | 26.97 | 64.85 |  |
| Thyroid cancer | 6.68 | 8.07 | 10.64 | 11.52 | 12.35 | 14.92 | 16.32 | 144.31 |  |
| Leukaemia | 33.89 | 33.92 | 38.17 | 35.14 | 34.75 | 35.43 | 34.16 | 0.80 |  |
| Multiple myeloma | 1.25 | 1.30 | 1.41 | 1.44 | 1.39 | 1.55 | 1.63 | 30.40 |  |
| **Cardiovascular diseases (%)** |  |  |  |  |  |  |  |  |  |
| Stroke | 0.96 | 0.98 | 1.00 | 1.00 | 1.01 | 1.03 | 1.05 | 9.70 |  |
| Ischemic heart disease | 0.84 | 0.89 | 0.93 | 0.94 | 0.95 | 0.97 | 1.00 | 18.95 |  |
| **Chronic respiratory diseases (%)** |  |  |  |  |  |  |  |  |  |
| Chronic obstructive pulmonary disease | 0.75 | 0.76 | 0.77 | 0.74 | 0.71 | 0.74 | 0.75 | 0.95 |  |
| Asthma | 3.54 | 3.16 | 3.18 | 2.98 | 2.85 | 2.87 | 3.37 | -4.95 |  |
| **Digestive diseases (%)** |  |  |  |  |  |  |  |  |  |
| Gallbladder and biliary diseases | 0.76 | 0.77 | 0.80 | 0.81 | 0.82 | 0.83 | 0.84 | 9.92 |  |
| Upper digestive system diseases | 3.79 | 3.90 | 4.01 | 4.10 | 4.18 | 4.27 | 4.37 | 15.04 |  |
| **Neurological disorders (%)** |  |  |  |  |  |  |  |  |  |
| Alzheimer's disease and other dementias | 0.11 | 0.11 | 0.12 | 0.13 | 0.13 | 0.13 | 0.14 | 26.59 |  |
| Parkinson's disease | 0.04 | 0.04 | 0.04 | 0.05 | 0.05 | 0.05 | 0.05 | 17.38 |  |
| **Mental disorders (%)** |  |  |  |  |  |  |  |  |  |
| Depressive disorders | 2.23 | 2.29 | 2.33 | 2.35 | 2.36 | 2.41 | 2.46 | 10.55 |  |
| Anxiety disorder | 3.38 | 3.43 | 3.49 | 3.46 | 3.54 | 3.63 | 3.69 | 9.04 |  |
| **Substance use disorders (%)** |  |  |  |  |  |  |  |  |  |
| Alcohol use disorders | 0.50 | 0.52 | 0.54 | 0.55 | 0.57 | 0.58 | 0.59 | 18.38 |  |
| Drug use disorders | 0.69 | 0.71 | 0.73 | 0.72 | 0.71 | 0.71 | 0.72 | 5.19 |  |
| **Diabetes and kidney diseases (%)** |  |  |  |  |  |  |  |  |  |
| Diabetes mellitus type 2 | 2.77 | 3.17 | 3.73 | 4.39 | 5.12 | 6.08 | 6.78 | 144.52 |  |
| Chronic kidney disease | 5.18 | 5.42 | 5.72 | 5.91 | 6.16 | 6.44 | 6.68 | 28.78 |  |
| **Musculoskeletal disorders (%)** |  |  |  |  |  |  |  |  |  |
| Osteoarthritis | 1.94 | 2.04 | 2.16 | 2.27 | 2.30 | 2.40 | 2.55 | 31.80 |  |
| **Tokelau (New Zealand)** |  |  |  |  |  |  |  |  |  |
| **Neoplasms/Cancer (per 100,000 persons)** |  |  |  |  |  |  |  |  |  |
| Lip and oral cavity cancer | 6.87 | 6.98 | 7.12 | 8.28 | 9.25 | 9.66 | 10.66 | 55.17 |  |
| Colon and rectum cancer | 26.98 | 27.91 | 29.21 | 36.03 | 40.53 | 44.32 | 50.38 | 86.73 |  |
| Stomach cancer | 12.45 | 12.19 | 11.33 | 12.36 | 12.10 | 11.60 | 11.79 | -5.30 |  |
| Pancreatic cancer | 1.48 | 1.64 | 1.78 | 2.38 | 2.49 | 2.67 | 3.06 | 106.76 |  |
| Non-Hodgkin's lymphoma | 0.00 | 0.00 | 0.00 | 0.00 | 0.08 | 0.36 | 1.14 |  |  |
| Gallbladder and biliary tract cancer | 1.12 | 1.09 | 1.03 | 1.12 | 1.09 | 1.03 | 1.08 | -3.57 |  |
| Kidney cancer | 4.03 | 4.35 | 5.01 | 5.95 | 6.23 | 7.00 | 8.01 | 98.76 |  |
| Ovarian cancer | 5.20 | 6.15 | 7.78 | 9.90 | 10.80 | 11.78 | 13.13 | 152.50 |  |
| Cervical cancer | 68.89 | 70.30 | 72.12 | 75.20 | 78.70 | 78.05 | 84.06 | 22.02 |  |
| Breast cancer | 127.23 | 133.40 | 152.60 | 186.52 | 210.01 | 222.97 | 244.33 | 92.04 |  |
| Oesophageal cancer | 2.31 | 2.25 | 2.04 | 2.43 | 2.32 | 2.39 | 2.45 | 6.06 |  |
| Tracheal, bronchus and lung cancer | 16.44 | 16.41 | 15.77 | 19.17 | 20.13 | 21.15 | 22.78 | 38.56 |  |
| Prostate cancer | 32.41 | 35.61 | 35.97 | 48.15 | 54.41 | 60.87 | 72.59 | 123.97 |  |
| Thyroid cancer | 8.15 | 9.45 | 11.27 | 13.96 | 16.10 | 17.10 | 19.84 | 143.44 |  |
| Leukaemia | 27.53 | 25.95 | 25.69 | 25.44 | 21.93 | 20.42 | 19.76 | -28.22 |  |
| Multiple myeloma | 1.85 | 1.77 | 1.71 | 1.98 | 2.07 | 2.20 | 2.37 | 28.11 |  |
| **Cardiovascular diseases (%)** |  |  |  |  |  |  |  |  |  |
| Stroke | 1.34 | 1.33 | 1.32 | 1.47 | 1.46 | 1.44 | 1.51 | 13.11 |  |
| Ischemic heart disease | 1.88 | 1.96 | 1.91 | 2.28 | 2.24 | 2.23 | 2.31 | 23.02 |  |
| **Chronic respiratory diseases (%)** |  |  |  |  |  |  |  |  |  |
| Chronic obstructive pulmonary disease | 2.51 | 2.58 | 2.54 | 2.94 | 2.92 | 2.88 | 2.95 | 17.38 |  |
| Asthma | 3.51 | 2.68 | 2.67 | 2.75 | 2.92 | 2.93 | 3.51 | 0.01 |  |
| **Digestive diseases (%)** |  |  |  |  |  |  |  |  |  |
| Gallbladder and biliary diseases | 0.87 | 0.84 | 0.84 | 0.89 | 0.87 | 0.86 | 0.89 | 2.22 |  |
| Upper digestive system diseases | 4.50 | 4.55 | 4.60 | 4.94 | 5.05 | 5.12 | 5.26 | 16.73 |  |
| **Neurological disorders (%)** |  |  |  |  |  |  |  |  |  |
| Alzheimer's disease and other dementias | 0.42 | 0.46 | 0.47 | 0.57 | 0.55 | 0.49 | 0.47 | 13.09 |  |
| Parkinson's disease | 0.10 | 0.10 | 0.10 | 0.12 | 0.12 | 0.11 | 0.12 | 15.63 |  |
| **Mental disorders (%)** |  |  |  |  |  |  |  |  |  |
| Depressive disorders | 2.54 | 2.58 | 2.63 | 2.77 | 2.87 | 2.90 | 2.94 | 15.51 |  |
| Anxiety disorder | 3.51 | 3.56 | 3.68 | 3.67 | 3.82 | 3.84 | 3.85 | 9.54 |  |
| **Substance use disorders (%)** |  |  |  |  |  |  |  |  |  |
| Alcohol use disorders | 0.54 | 0.56 | 0.58 | 0.63 | 0.66 | 0.67 | 0.69 | 28.46 |  |
| Drug use disorders | 0.62 | 0.63 | 0.65 | 0.65 | 0.69 | 0.67 | 0.67 | 7.70 |  |
| **Diabetes and kidney diseases (%)** |  |  |  |  |  |  |  |  |  |
| Diabetes mellitus type 2 | 4.74 | 5.34 | 6.04 | 7.67 | 8.47 | 9.39 | 10.49 | 121.25 |  |
| Chronic kidney disease | 7.08 | 7.48 | 7.80 | 8.85 | 9.24 | 9.56 | 10.09 | 42.51 |  |
| **Musculoskeletal disorders (%)** |  |  |  |  |  |  |  |  |  |
| Osteoarthritis | 3.76 | 3.81 | 3.86 | 4.69 | 4.74 | 4.89 | 5.16 | 36.95 |  |
| **Tonga** |  |  |  |  |  |  |  |  |  |
| **Neoplasms/Cancer (per 100,000 persons)** |  |  |  |  |  |  |  |  |  |
| Lip and oral cavity cancer | 4.51 | 4.83 | 5.55 | 5.60 | 6.11 | 6.74 | 7.37 | 63.41 |  |
| Colon and rectum cancer | 13.04 | 14.71 | 17.53 | 17.16 | 18.32 | 20.49 | 23.04 | 76.69 |  |
| Stomach cancer | 11.04 | 11.55 | 13.62 | 12.36 | 12.72 | 12.98 | 13.37 | 21.11 |  |
| Pancreatic cancer | 1.19 | 1.57 | 2.12 | 2.17 | 2.04 | 2.22 | 2.47 | 107.56 |  |
| Non-Hodgkin's lymphoma | 2.22 | 2.47 | 3.71 | 3.33 | 3.47 | 3.97 | 4.98 | 124.32 |  |
| Gallbladder and biliary tract cancer | 0.53 | 0.57 | 0.66 | 0.61 | 0.64 | 0.67 | 0.71 | 33.96 |  |
| Kidney cancer | 3.53 | 4.34 | 5.83 | 5.93 | 5.47 | 5.65 | 6.22 | 76.20 |  |
| Ovarian cancer | 5.00 | 6.10 | 7.58 | 8.63 | 8.80 | 9.96 | 11.16 | 123.20 |  |
| Cervical cancer | 57.21 | 58.82 | 60.08 | 53.72 | 56.61 | 60.14 | 64.08 | 12.01 |  |
| Breast cancer | 113.38 | 127.45 | 144.22 | 144.01 | 154.80 | 173.74 | 192.63 | 69.90 |  |
| Oesophageal cancer | 1.59 | 1.82 | 2.24 | 2.09 | 2.01 | 2.07 | 2.19 | 37.74 |  |
| Tracheal, bronchus and lung cancer | 14.78 | 15.88 | 18.76 | 17.43 | 18.37 | 19.59 | 20.72 | 40.19 |  |
| Prostate cancer | 36.02 | 42.88 | 55.46 | 53.14 | 57.49 | 62.74 | 70.03 | 94.42 |  |
| Thyroid cancer | 5.20 | 6.28 | 8.14 | 8.50 | 8.60 | 9.31 | 10.47 | 101.35 |  |
| Leukaemia | 14.15 | 14.39 | 16.98 | 15.69 | 15.14 | 14.33 | 14.01 | -0.99 |  |
| Multiple myeloma | 1.75 | 1.96 | 2.29 | 2.20 | 2.20 | 2.33 | 2.54 | 45.14 |  |
| **Cardiovascular diseases (%)** |  |  |  |  |  |  |  |  |  |
| Stroke | 0.95 | 0.96 | 1.01 | 0.98 | 1.02 | 1.05 | 1.14 | 19.52 |  |
| Ischemic heart disease | 1.40 | 1.57 | 1.66 | 1.71 | 1.80 | 1.90 | 2.00 | 42.54 |  |
| **Chronic respiratory diseases (%)** |  |  |  |  |  |  |  |  |  |
| Chronic obstructive pulmonary disease | 0.84 | 0.88 | 0.90 | 0.90 | 0.88 | 0.92 | 0.96 | 13.21 |  |
| Asthma | 4.36 | 3.95 | 4.09 | 3.88 | 3.43 | 3.37 | 3.71 | -14.83 |  |
| **Digestive diseases (%)** |  |  |  |  |  |  |  |  |  |
| Gallbladder and biliary diseases | 0.82 | 0.82 | 0.83 | 0.83 | 0.83 | 0.87 | 0.90 | 9.76 |  |
| Upper digestive system diseases | 4.25 | 4.39 | 4.49 | 4.56 | 4.62 | 4.75 | 4.90 | 15.14 |  |
| **Neurological disorders (%)** |  |  |  |  |  |  |  |  |  |
| Alzheimer's disease and other dementias | 0.25 | 0.28 | 0.31 | 0.33 | 0.37 | 0.40 | 0.43 | 75.35 |  |
| Parkinson's disease | 0.06 | 0.07 | 0.07 | 0.07 | 0.08 | 0.09 | 0.09 | 40.66 |  |
| **Mental disorders (%)** |  |  |  |  |  |  |  |  |  |
| Depressive disorders | 2.15 | 2.19 | 2.22 | 2.24 | 2.25 | 2.32 | 2.40 | 11.73 |  |
| Anxiety disorder | 3.64 | 3.68 | 3.71 | 3.68 | 3.74 | 3.84 | 3.89 | 6.83 |  |
| **Substance use disorders (%)** |  |  |  |  |  |  |  |  |  |
| Alcohol use disorders | 0.47 | 0.49 | 0.52 | 0.55 | 0.56 | 0.56 | 0.58 | 22.72 |  |
| Drug use disorders | 0.72 | 0.79 | 0.80 | 0.79 | 0.78 | 0.78 | 0.79 | 10.38 |  |
| **Diabetes and kidney diseases (%)** |  |  |  |  |  |  |  |  |  |
| Diabetes mellitus type 2 | 4.08 | 4.69 | 5.37 | 6.12 | 6.72 | 7.61 | 8.51 | 108.39 |  |
| Chronic kidney disease | 6.55 | 7.02 | 7.49 | 7.86 | 8.26 | 8.74 | 9.24 | 40.94 |  |
| **Musculoskeletal disorders (%)** |  |  |  |  |  |  |  |  |  |
| Osteoarthritis | 2.99 | 3.29 | 3.53 | 3.71 | 3.76 | 4.01 | 4.29 | 43.45 |  |
| **Malaysia** |  |  |  |  |  |  |  |  |  |
| **Neoplasms/Cancer (per 100,000 persons)** |  |  |  |  |  |  |  |  |  |
| Lip and oral cavity cancer | 10.39 | 10.80 | 10.76 | 10.97 | 12.31 | 15.29 | 19.36 | 86.33 |  |
| Colon and rectum cancer | 32.83 | 37.71 | 43.64 | 50.79 | 61.66 | 80.53 | 108.82 | 231.47 |  |
| Stomach cancer | 6.50 | 6.61 | 6.44 | 6.41 | 6.79 | 7.45 | 8.88 | 36.62 |  |
| Pancreatic cancer | 0.71 | 0.85 | 1.11 | 1.42 | 1.82 | 2.08 | 2.35 | 230.99 |  |
| Non-Hodgkin's lymphoma | 0.77 | 1.14 | 3.14 | 5.20 | 7.67 | 12.48 | 17.87 | 2220.78 |  |
| Gallbladder and biliary tract cancer | 0.80 | 0.82 | 0.82 | 0.87 | 1.01 | 1.19 | 1.40 | 75.00 |  |
| Kidney cancer | 4.40 | 5.08 | 6.35 | 7.90 | 9.96 | 12.74 | 15.71 | 257.05 |  |
| Ovarian cancer | 6.70 | 8.07 | 10.69 | 13.94 | 16.14 | 17.62 | 18.13 | 170.60 |  |
| Cervical cancer | 33.32 | 33.67 | 33.10 | 32.17 | 33.24 | 38.81 | 44.72 | 34.21 |  |
| Breast cancer | 86.53 | 95.72 | 110.24 | 133.56 | 161.55 | 199.95 | 236.90 | 173.78 |  |
| Oesophageal cancer | 2.00 | 2.06 | 2.11 | 2.19 | 2.38 | 2.81 | 3.48 | 74.00 |  |
| Tracheal, bronchus and lung cancer | 8.60 | 9.72 | 11.30 | 12.80 | 14.80 | 16.01 | 17.71 | 105.93 |  |
| Prostate cancer | 11.67 | 13.71 | 16.81 | 22.36 | 28.80 | 39.11 | 50.81 | 335.39 |  |
| Thyroid cancer | 13.39 | 15.23 | 17.84 | 21.43 | 24.41 | 28.86 | 35.43 | 164.60 |  |
| Leukaemia | 25.68 | 22.44 | 19.81 | 18.82 | 18.03 | 19.98 | 21.86 | -14.88 |  |
| Multiple myeloma | 0.91 | 0.99 | 1.12 | 1.33 | 1.61 | 1.89 | 2.16 | 137.36 |  |
| **Cardiovascular diseases (%)** |  |  |  |  |  |  |  |  |  |
| Stroke | 1.13 | 1.07 | 1.05 | 1.09 | 1.16 | 1.29 | 1.42 | 25.16 |  |
| Ischemic heart disease | 1.10 | 1.08 | 1.16 | 1.27 | 1.47 | 1.66 | 1.83 | 67.29 |  |
| **Chronic respiratory diseases (%)** |  |  |  |  |  |  |  |  |  |
| Chronic obstructive pulmonary disease | 1.18 | 1.20 | 1.27 | 1.40 | 1.52 | 1.66 | 1.75 | 48.98 |  |
| Asthma | 3.01 | 3.07 | 2.92 | 2.84 | 2.77 | 2.48 | 2.53 | -15.82 |  |
| **Digestive diseases (%)** |  |  |  |  |  |  |  |  |  |
| Gallbladder and biliary diseases | 1.03 | 1.04 | 1.04 | 1.05 | 1.09 | 1.12 | 1.17 | 13.56 |  |
| Upper digestive system diseases | 4.40 | 4.52 | 4.70 | 4.93 | 5.19 | 5.45 | 5.65 | 28.64 |  |
| **Neurological disorders (%)** |  |  |  |  |  |  |  |  |  |
| Alzheimer's disease and other dementias | 0.23 | 0.23 | 0.24 | 0.26 | 0.30 | 0.36 | 0.41 | 75.94 |  |
| Parkinson's disease | 0.04 | 0.04 | 0.05 | 0.05 | 0.06 | 0.07 | 0.08 | 88.97 |  |
| **Mental disorders (%)** |  |  |  |  |  |  |  |  |  |
| Depressive disorders | 2.70 | 2.75 | 2.70 | 3.29 | 3.37 | 3.69 | 3.78 | 39.88 |  |
| Anxiety disorder | 4.03 | 4.11 | 4.38 | 4.52 | 4.65 | 4.70 | 4.76 | 18.14 |  |
| **Substance use disorders (%)** |  |  |  |  |  |  |  |  |  |
| Alcohol use disorders | 0.48 | 0.50 | 0.52 | 0.53 | 0.52 | 0.54 | 0.56 | 17.07 |  |
| Drug use disorders | 0.56 | 0.57 | 0.57 | 0.60 | 0.62 | 0.65 | 0.66 | 18.09 |  |
| **Diabetes and kidney diseases (%)** |  |  |  |  |  |  |  |  |  |
| Diabetes mellitus type 2 | 2.58 | 2.91 | 3.46 | 4.06 | 4.38 | 5.55 | 5.80 | 124.39 |  |
| Chronic kidney disease | 6.18 | 6.50 | 6.97 | 7.61 | 8.40 | 9.25 | 9.99 | 61.50 |  |
| **Musculoskeletal disorders (%)** |  |  |  |  |  |  |  |  |  |
| Osteoarthritis | 2.17 | 2.27 | 2.48 | 2.79 | 3.18 | 3.58 | 3.92 | 80.54 |  |
| **Tuvalu** |  |  |  |  |  |  |  |  |  |
| **Neoplasms/Cancer (per 100,000 persons)** |  |  |  |  |  |  |  |  |  |
| Lip and oral cavity cancer | 7.86 | 8.50 | 8.64 | 8.79 | 9.42 | 9.76 | 10.44 | 32.82 |  |
| Colon and rectum cancer | 25.30 | 29.54 | 31.82 | 32.55 | 34.52 | 36.19 | 39.74 | 57.08 |  |
| Stomach cancer | 16.70 | 16.56 | 16.03 | 15.13 | 15.06 | 14.82 | 15.00 | -10.18 |  |
| Pancreatic cancer | 1.39 | 1.71 | 1.93 | 2.14 | 2.16 | 2.30 | 2.52 | 81.29 |  |
| Non-Hodgkin's lymphoma | 0.00 | 0.00 | 0.00 | 0.00 | 0.00 | 0.00 | 0.00 |  |  |
| Gallbladder and biliary tract cancer | 1.18 | 1.22 | 1.20 | 1.12 | 1.09 | 1.08 | 1.09 | -7.63 |  |
| Kidney cancer | 4.53 | 5.27 | 5.70 | 5.53 | 5.64 | 5.80 | 6.45 | 42.38 |  |
| Ovarian cancer | 6.75 | 8.46 | 10.08 | 11.49 | 11.88 | 12.47 | 13.23 | 96.00 |  |
| Cervical cancer | 83.92 | 85.58 | 82.70 | 76.17 | 75.09 | 70.92 | 70.87 | -15.55 |  |
| Breast cancer | 134.56 | 151.58 | 168.85 | 177.46 | 188.62 | 193.23 | 203.89 | 51.52 |  |
| Oesophageal cancer | 2.91 | 3.00 | 2.91 | 2.74 | 2.65 | 2.65 | 2.74 | -5.84 |  |
| Tracheal, bronchus and lung cancer | 17.98 | 19.34 | 19.91 | 19.96 | 20.71 | 21.32 | 22.42 | 24.69 |  |
| Prostate cancer | 30.19 | 37.82 | 41.64 | 41.50 | 45.58 | 49.94 | 58.12 | 92.51 |  |
| Thyroid cancer | 9.07 | 11.19 | 13.07 | 13.70 | 14.46 | 14.56 | 15.74 | 73.54 |  |
| Leukaemia | 41.16 | 35.90 | 31.87 | 27.18 | 25.91 | 23.48 | 22.59 | -45.12 |  |
| Multiple myeloma | 1.97 | 2.08 | 2.06 | 1.98 | 1.99 | 2.05 | 2.17 | 10.15 |  |
| **Cardiovascular diseases (%)** |  |  |  |  |  |  |  |  |  |
| Stroke | 1.35 | 1.42 | 1.45 | 1.43 | 1.44 | 1.43 | 1.47 | 8.89 |  |
| Ischemic heart disease | 1.56 | 1.74 | 1.85 | 1.89 | 1.91 | 1.95 | 2.01 | 29.14 |  |
| **Chronic respiratory diseases (%)** |  |  |  |  |  |  |  |  |  |
| Chronic obstructive pulmonary disease | 2.17 | 2.37 | 2.42 | 2.41 | 2.38 | 2.39 | 2.45 | 13.15 |  |
| Asthma | 3.06 | 2.79 | 2.63 | 2.46 | 2.30 | 2.25 | 3.35 | 9.47 |  |
| **Digestive diseases (%)** |  |  |  |  |  |  |  |  |  |
| Gallbladder and biliary diseases | 1.00 | 0.99 | 0.98 | 0.96 | 0.94 | 0.93 | 0.94 | -5.91 |  |
| Upper digestive system diseases | 4.99 | 5.06 | 5.11 | 5.14 | 5.17 | 5.27 | 5.40 | 8.38 |  |
| **Neurological disorders (%)** |  |  |  |  |  |  |  |  |  |
| Alzheimer's disease and other dementias | 0.27 | 0.31 | 0.33 | 0.35 | 0.36 | 0.36 | 0.37 | 39.87 |  |
| Parkinson's disease | 0.07 | 0.08 | 0.09 | 0.09 | 0.10 | 0.10 | 0.10 | 35.46 |  |
| **Mental disorders (%)** |  |  |  |  |  |  |  |  |  |
| Depressive disorders | 2.84 | 2.87 | 2.89 | 2.91 | 2.95 | 3.01 | 3.07 | 7.86 |  |
| Anxiety disorder | 3.74 | 3.78 | 3.82 | 3.82 | 3.91 | 4.01 | 4.04 | 8.05 |  |
| **Substance use disorders (%)** |  |  |  |  |  |  |  |  |  |
| Alcohol use disorders | 0.61 | 0.63 | 0.65 | 0.66 | 0.68 | 0.70 | 0.73 | 20.12 |  |
| Drug use disorders | 0.68 | 0.66 | 0.67 | 0.68 | 0.71 | 0.73 | 0.76 | 11.83 |  |
| **Diabetes and kidney diseases (%)** |  |  |  |  |  |  |  |  |  |
| Diabetes mellitus type 2 | 4.96 | 5.85 | 6.89 | 7.92 | 8.83 | 9.80 | 10.69 | 115.40 |  |
| Chronic kidney disease | 7.42 | 8.03 | 8.47 | 8.71 | 8.96 | 9.29 | 9.75 | 31.29 |  |
| **Musculoskeletal disorders (%)** |  |  |  |  |  |  |  |  |  |
| Osteoarthritis | 3.61 | 3.96 | 4.21 | 4.40 | 4.44 | 4.54 | 4.66 | 29.04 |  |
| **Marshall Islands** |  |  |  |  |  |  |  |  |  |
| **Neoplasms/Cancer (per 100,000 persons)** |  |  |  |  |  |  |  |  |  |
| Lip and oral cavity cancer | 4.52 | 4.69 | 4.97 | 5.94 | 6.90 | 7.82 | 8.73 | 93.14 |  |
| Colon and rectum cancer | 13.59 | 14.32 | 15.50 | 18.56 | 22.13 | 25.96 | 29.74 | 118.84 |  |
| Stomach cancer | 10.04 | 9.88 | 10.61 | 11.75 | 12.73 | 13.41 | 14.24 | 41.83 |  |
| Pancreatic cancer | 0.62 | 0.70 | 0.83 | 1.06 | 1.28 | 1.56 | 1.82 | 193.55 |  |
| Non-Hodgkin's lymphoma | 0.01 | 0.00 | 0.00 | 0.00 | 0.00 | 0.00 | 0.01 | 0.00 |  |
| Gallbladder and biliary tract cancer | 0.57 | 0.56 | 0.60 | 0.67 | 0.74 | 0.82 | 0.90 | 57.89 |  |
| Kidney cancer | 2.39 | 2.70 | 2.91 | 3.49 | 3.79 | 4.22 | 4.80 | 100.84 |  |
| Ovarian cancer | 3.34 | 4.61 | 5.97 | 7.90 | 9.03 | 10.63 | 11.96 | 258.08 |  |
| Cervical cancer | 39.94 | 48.27 | 53.27 | 62.59 | 69.19 | 73.34 | 77.57 | 94.22 |  |
| Breast cancer | 55.38 | 65.50 | 79.24 | 101.05 | 122.37 | 143.41 | 164.00 | 196.14 |  |
| Oesophageal cancer | 1.45 | 1.41 | 1.56 | 1.77 | 1.95 | 2.16 | 2.36 | 62.76 |  |
| Tracheal, bronchus and lung cancer | 9.93 | 9.41 | 10.50 | 12.29 | 14.59 | 16.75 | 18.63 | 87.61 |  |
| Prostate cancer | 15.80 | 12.48 | 13.33 | 16.43 | 20.21 | 25.86 | 31.81 | 101.33 |  |
| Thyroid cancer | 4.51 | 5.71 | 6.71 | 8.71 | 9.99 | 11.25 | 12.64 | 180.27 |  |
| Leukaemia | 24.33 | 25.46 | 26.85 | 28.97 | 27.38 | 25.29 | 24.76 | 1.77 |  |
| Multiple myeloma | 0.96 | 0.93 | 1.01 | 1.19 | 1.36 | 1.55 | 1.73 | 80.21 |  |
| **Cardiovascular diseases (%)** |  |  |  |  |  |  |  |  |  |
| Stroke | 0.92 | 0.96 | 1.06 | 1.15 | 1.26 | 1.38 | 1.47 | 60.04 |  |
| Ischemic heart disease | 0.74 | 0.76 | 0.80 | 0.88 | 1.00 | 1.15 | 1.28 | 72.81 |  |
| **Chronic respiratory diseases (%)** |  |  |  |  |  |  |  |  |  |
| Chronic obstructive pulmonary disease | 0.85 | 0.84 | 0.84 | 0.88 | 0.91 | 0.98 | 1.04 | 21.70 |  |
| Asthma | 3.60 | 3.24 | 3.06 | 2.93 | 2.65 | 2.55 | 2.96 | -17.81 |  |
| **Digestive diseases (%)** |  |  |  |  |  |  |  |  |  |
| Gallbladder and biliary diseases | 0.71 | 0.74 | 0.79 | 0.84 | 0.87 | 0.92 | 0.96 | 35.02 |  |
| Upper digestive system diseases | 3.63 | 3.79 | 4.04 | 4.32 | 4.56 | 4.80 | 5.01 | 38.14 |  |
| **Neurological disorders (%)** |  |  |  |  |  |  |  |  |  |
| Alzheimer's disease and other dementias | 0.12 | 0.12 | 0.12 | 0.12 | 0.13 | 0.15 | 0.17 | 36.79 |  |
| Parkinson's disease | 0.04 | 0.04 | 0.04 | 0.04 | 0.05 | 0.06 | 0.07 | 54.53 |  |
| **Mental disorders (%)** |  |  |  |  |  |  |  |  |  |
| Depressive disorders | 2.04 | 2.15 | 2.30 | 2.43 | 2.50 | 2.59 | 2.68 | 31.25 |  |
| Anxiety disorder | 3.35 | 3.52 | 3.65 | 3.66 | 3.76 | 3.85 | 3.93 | 17.01 |  |
| **Substance use disorders (%)** |  |  |  |  |  |  |  |  |  |
| Alcohol use disorders | 0.51 | 0.55 | 0.61 | 0.66 | 0.70 | 0.72 | 0.75 | 46.92 |  |
| Drug use disorders | 0.67 | 0.72 | 0.77 | 0.79 | 0.79 | 0.77 | 0.77 | 13.85 |  |
| **Diabetes and kidney diseases (%)** |  |  |  |  |  |  |  |  |  |
| Diabetes mellitus type 2 | 5.19 | 6.19 | 8.08 | 9.88 | 11.41 | 13.71 | 15.49 | 198.25 |  |
| Chronic kidney disease | 4.94 | 5.25 | 5.66 | 6.24 | 6.88 | 7.58 | 8.19 | 65.68 |  |
| **Musculoskeletal disorders (%)** |  |  |  |  |  |  |  |  |  |
| Osteoarthritis | 1.68 | 1.76 | 1.97 | 2.29 | 2.61 | 3.01 | 3.37 | 100.14 |  |
| **Vanuatu** |  |  |  |  |  |  |  |  |  |
| **Neoplasms/Cancer (per 100,000 persons)** |  |  |  |  |  |  |  |  |  |
| Lip and oral cavity cancer | 3.90 | 4.51 | 4.68 | 4.55 | 5.29 | 5.82 | 6.22 | 59.49 |  |
| Colon and rectum cancer | 11.24 | 12.91 | 13.63 | 13.33 | 15.93 | 18.08 | 19.63 | 74.64 |  |
| Stomach cancer | 9.30 | 10.50 | 10.51 | 10.29 | 11.11 | 11.63 | 11.94 | 28.39 |  |
| Pancreatic cancer | 0.58 | 0.69 | 0.76 | 0.83 | 1.02 | 1.21 | 1.32 | 127.59 |  |
| Non-Hodgkin's lymphoma | 0.00 | 0.00 | 0.00 | 0.00 | 0.00 | 0.00 | 0.00 |  |  |
| Gallbladder and biliary tract cancer | 0.51 | 0.56 | 0.57 | 0.54 | 0.61 | 0.67 | 0.70 | 37.25 |  |
| Kidney cancer | 2.12 | 2.42 | 2.80 | 2.53 | 2.98 | 3.17 | 3.42 | 61.32 |  |
| Ovarian cancer | 2.06 | 2.87 | 3.44 | 3.84 | 4.79 | 5.55 | 6.01 | 191.75 |  |
| Cervical cancer | 31.52 | 40.48 | 42.62 | 39.28 | 43.94 | 45.42 | 46.77 | 48.38 |  |
| Breast cancer | 41.72 | 48.38 | 54.24 | 57.06 | 72.31 | 82.47 | 89.66 | 114.91 |  |
| Oesophageal cancer | 1.51 | 1.68 | 1.66 | 1.65 | 1.82 | 1.98 | 2.07 | 37.09 |  |
| Tracheal, bronchus and lung cancer | 8.82 | 9.69 | 9.85 | 10.09 | 12.03 | 13.47 | 14.26 | 61.68 |  |
| Prostate cancer | 14.89 | 15.92 | 17.45 | 17.30 | 21.04 | 24.77 | 27.99 | 87.98 |  |
| Thyroid cancer | 3.70 | 4.98 | 5.99 | 5.66 | 6.72 | 7.33 | 7.86 | 112.43 |  |
| Leukaemia | 20.66 | 22.71 | 24.88 | 22.91 | 24.24 | 23.09 | 22.95 | 11.08 |  |
| Multiple myeloma | 0.90 | 0.99 | 0.99 | 0.96 | 1.11 | 1.25 | 1.32 | 46.67 |  |
| **Cardiovascular diseases (%)** |  |  |  |  |  |  |  |  |  |
| Stroke | 1.23 | 1.23 | 1.26 | 1.29 | 1.35 | 1.40 | 1.44 | 16.78 |  |
| Ischemic heart disease | 1.13 | 1.14 | 1.16 | 1.22 | 1.33 | 1.43 | 1.52 | 34.65 |  |
| **Chronic respiratory diseases (%)** |  |  |  |  |  |  |  |  |  |
| Chronic obstructive pulmonary disease | 0.98 | 0.95 | 0.95 | 0.93 | 0.94 | 0.98 | 1.03 | 5.40 |  |
| Asthma | 3.24 | 2.82 | 2.73 | 2.41 | 2.24 | 2.18 | 3.03 | -6.43 |  |
| **Digestive diseases (%)** |  |  |  |  |  |  |  |  |  |
| Gallbladder and biliary diseases | 0.91 | 0.92 | 0.96 | 0.98 | 1.01 | 1.02 | 1.03 | 12.90 |  |
| Upper digestive system diseases | 4.13 | 4.19 | 4.29 | 4.40 | 4.52 | 4.60 | 4.69 | 13.71 |  |
| **Neurological disorders (%)** |  |  |  |  |  |  |  |  |  |
| Alzheimer's disease and other dementias | 0.15 | 0.15 | 0.15 | 0.15 | 0.17 | 0.19 | 0.21 | 46.19 |  |
| Parkinson's disease | 0.05 | 0.05 | 0.05 | 0.05 | 0.06 | 0.06 | 0.07 | 35.91 |  |
| **Mental disorders (%)** |  |  |  |  |  |  |  |  |  |
| Depressive disorders | 2.33 | 2.36 | 2.39 | 2.44 | 2.47 | 2.50 | 2.53 | 8.50 |  |
| Anxiety disorder | 3.42 | 3.47 | 3.55 | 3.58 | 3.67 | 3.73 | 3.78 | 10.48 |  |
| **Substance use disorders (%)** |  |  |  |  |  |  |  |  |  |
| Alcohol use disorders | 0.59 | 0.60 | 0.62 | 0.64 | 0.66 | 0.66 | 0.67 | 14.56 |  |
| Drug use disorders | 0.57 | 0.58 | 0.59 | 0.60 | 0.60 | 0.60 | 0.60 | 5.42 |  |
| **Diabetes and kidney diseases (%)** |  |  |  |  |  |  |  |  |  |
| Diabetes mellitus type 2 | 3.09 | 3.46 | 4.02 | 4.72 | 5.51 | 6.45 | 7.24 | 134.18 |  |
| Chronic kidney disease | 5.49 | 5.69 | 5.97 | 6.26 | 6.67 | 7.04 | 7.40 | 34.84 |  |
| **Musculoskeletal disorders (%)** |  |  |  |  |  |  |  |  |  |
| Osteoarthritis | 2.11 | 2.17 | 2.28 | 2.48 | 2.71 | 2.92 | 3.07 | 45.18 |  |
| **Micronesia, Federated States of** |  |  |  |  |  |  |  |  |  |
| **Neoplasms/Cancer (per 100,000 persons)** |  |  |  |  |  |  |  |  |  |
| Lip and oral cavity cancer | 5.55 | 5.93 | 6.02 | 7.15 | 8.14 | 9.47 | 10.66 | 92.07 |  |
| Colon and rectum cancer | 17.90 | 19.53 | 20.70 | 24.77 | 28.73 | 34.27 | 39.79 | 122.29 |  |
| Stomach cancer | 12.41 | 12.27 | 12.03 | 12.63 | 13.79 | 15.11 | 16.52 | 33.12 |  |
| Pancreatic cancer | 1.06 | 1.28 | 1.49 | 1.85 | 2.10 | 2.41 | 2.76 | 160.38 |  |
| Non-Hodgkin's lymphoma | 0.00 | 0.01 | 0.02 | 0.02 | 0.02 | 0.02 | 0.05 |  |  |
| Gallbladder and biliary tract cancer | 0.74 | 0.75 | 0.75 | 0.78 | 0.87 | 0.98 | 1.08 | 45.95 |  |
| Kidney cancer | 3.50 | 4.14 | 4.42 | 5.38 | 5.49 | 6.20 | 6.91 | 97.43 |  |
| Ovarian cancer | 4.05 | 5.70 | 7.62 | 10.48 | 11.81 | 13.83 | 15.33 | 278.52 |  |
| Cervical cancer | 47.65 | 51.89 | 53.28 | 60.65 | 65.55 | 71.19 | 76.25 | 60.02 |  |
| Breast cancer | 73.00 | 83.65 | 99.91 | 130.09 | 155.83 | 183.33 | 207.69 | 184.51 |  |
| Oesophageal cancer | 2.11 | 2.16 | 2.18 | 2.34 | 2.55 | 2.82 | 3.13 | 48.34 |  |
| Tracheal, bronchus and lung cancer | 12.76 | 12.93 | 13.66 | 14.98 | 17.41 | 20.40 | 23.02 | 80.41 |  |
| Prostate cancer | 18.71 | 19.63 | 19.99 | 24.34 | 27.92 | 35.94 | 45.23 | 141.74 |  |
| Thyroid cancer | 5.99 | 7.47 | 8.71 | 11.75 | 12.89 | 14.90 | 17.01 | 183.97 |  |
| Leukaemia | 28.70 | 28.37 | 26.05 | 26.18 | 25.09 | 24.76 | 24.69 | -13.97 |  |
| Multiple myeloma | 1.33 | 1.38 | 1.38 | 1.56 | 1.75 | 2.03 | 2.31 | 73.68 |  |
| **Cardiovascular diseases (%)** |  |  |  |  |  |  |  |  |  |
| Stroke | 1.11 | 1.10 | 1.11 | 1.16 | 1.24 | 1.34 | 1.42 | 27.93 |  |
| Ischemic heart disease | 0.95 | 0.97 | 0.99 | 1.05 | 1.15 | 1.27 | 1.38 | 45.03 |  |
| **Chronic respiratory diseases (%)** |  |  |  |  |  |  |  |  |  |
| Chronic obstructive pulmonary disease | 0.94 | 0.94 | 0.92 | 0.96 | 0.95 | 1.02 | 1.07 | 13.87 |  |
| Asthma | 3.61 | 3.32 | 3.08 | 3.00 | 2.70 | 2.66 | 3.07 | -14.80 |  |
| **Digestive diseases (%)** |  |  |  |  |  |  |  |  |  |
| Gallbladder and biliary diseases | 0.83 | 0.84 | 0.85 | 0.87 | 0.89 | 0.95 | 0.99 | 18.13 |  |
| Upper digestive system diseases | 3.95 | 4.05 | 4.18 | 4.38 | 4.61 | 4.89 | 5.13 | 29.72 |  |
| **Neurological disorders (%)** |  |  |  |  |  |  |  |  |  |
| Alzheimer's disease and other dementias | 0.15 | 0.16 | 0.17 | 0.18 | 0.19 | 0.21 | 0.22 | 47.84 |  |
| Parkinson's disease | 0.05 | 0.05 | 0.05 | 0.06 | 0.06 | 0.07 | 0.08 | 51.02 |  |
| **Mental disorders (%)** |  |  |  |  |  |  |  |  |  |
| Depressive disorders | 2.22 | 2.29 | 2.34 | 2.42 | 2.52 | 2.65 | 2.76 | 24.00 |  |
| Anxiety disorder | 3.47 | 3.55 | 3.66 | 3.69 | 3.83 | 3.96 | 4.02 | 15.96 |  |
| **Substance use disorders (%)** |  |  |  |  |  |  |  |  |  |
| Alcohol use disorders | 0.54 | 0.56 | 0.58 | 0.61 | 0.64 | 0.66 | 0.70 | 28.52 |  |
| Drug use disorders | 0.72 | 0.76 | 0.78 | 0.79 | 0.79 | 0.79 | 0.81 | 12.09 |  |
| **Diabetes and kidney diseases (%)** |  |  |  |  |  |  |  |  |  |
| Diabetes mellitus type 2 | 3.03 | 3.65 | 5.35 | 7.22 | 7.61 | 8.81 | 10.03 | 231.53 |  |
| Chronic kidney disease | 5.72 | 6.11 | 6.53 | 7.19 | 7.84 | 8.60 | 9.23 | 61.34 |  |
| **Musculoskeletal disorders (%)** |  |  |  |  |  |  |  |  |  |
| Osteoarthritis | 2.23 | 2.35 | 2.58 | 2.92 | 3.28 | 3.68 | 4.01 | 80.00 |  |
| **Viet Nam** |  |  |  |  |  |  |  |  |  |
| **Neoplasms/Cancer (per 100,000 persons)** |  |  |  |  |  |  |  |  |  |
| Lip and oral cavity cancer | 10.44 | 11.59 | 13.18 | 16.25 | 20.03 | 25.35 | 30.24 | 189.66 |  |
| Colon and rectum cancer | 20.01 | 23.61 | 30.33 | 45.65 | 67.51 | 91.16 | 117.48 | 487.11 |  |
| Stomach cancer | 13.75 | 13.74 | 13.12 | 12.64 | 12.59 | 14.37 | 16.40 | 19.27 |  |
| Pancreatic cancer | 0.75 | 0.83 | 0.99 | 1.45 | 2.07 | 2.60 | 3.15 | 320.00 |  |
| Non-Hodgkin's lymphoma | 0.00 | 0.00 | 0.00 | 0.13 | 1.27 | 1.87 | 3.65 |  |  |
| Gallbladder and biliary tract cancer | 0.75 | 0.74 | 0.80 | 1.05 | 1.36 | 1.58 | 1.80 | 140.00 |  |
| Kidney cancer | 2.83 | 3.07 | 3.61 | 5.16 | 7.15 | 9.35 | 11.56 | 308.48 |  |
| Ovarian cancer | 5.33 | 6.47 | 7.84 | 10.27 | 12.83 | 15.96 | 18.47 | 246.53 |  |
| Cervical cancer | 27.36 | 30.39 | 33.37 | 39.33 | 44.84 | 50.24 | 55.22 | 101.83 |  |
| Breast cancer | 73.10 | 76.87 | 91.16 | 124.44 | 159.38 | 200.56 | 244.96 | 235.10 |  |
| Oesophageal cancer | 1.91 | 1.94 | 2.07 | 2.70 | 3.61 | 4.23 | 4.79 | 150.79 |  |
| Tracheal, bronchus and lung cancer | 13.16 | 13.81 | 14.85 | 17.35 | 20.98 | 25.58 | 30.08 | 128.57 |  |
| Prostate cancer | 8.69 | 10.43 | 13.35 | 17.67 | 22.73 | 29.86 | 39.40 | 353.39 |  |
| Thyroid cancer | 11.01 | 12.52 | 15.48 | 28.67 | 47.26 | 57.12 | 66.50 | 504.00 |  |
| Leukaemia | 13.68 | 12.26 | 11.38 | 12.76 | 13.47 | 14.39 | 15.48 | 13.16 |  |
| Multiple myeloma | 0.73 | 0.76 | 0.83 | 1.04 | 1.35 | 1.73 | 2.09 | 186.30 |  |
| **Cardiovascular diseases (%)** |  |  |  |  |  |  |  |  |  |
| Stroke | 0.91 | 0.92 | 1.00 | 1.07 | 1.21 | 1.33 | 1.54 | 69.47 |  |
| Ischemic heart disease | 0.96 | 0.96 | 1.02 | 1.14 | 1.30 | 1.41 | 1.56 | 62.75 |  |
| **Chronic respiratory diseases (%)** |  |  |  |  |  |  |  |  |  |
| Chronic obstructive pulmonary disease | 1.30 | 1.42 | 1.61 | 1.86 | 2.12 | 2.44 | 2.73 | 109.45 |  |
| Asthma | 2.51 | 2.49 | 2.86 | 2.83 | 2.55 | 2.75 | 2.96 | 17.59 |  |
| **Digestive diseases (%)** |  |  |  |  |  |  |  |  |  |
| Gallbladder and biliary diseases | 1.08 | 1.07 | 1.07 | 1.09 | 1.19 | 1.25 | 1.34 | 24.54 |  |
| Upper digestive system diseases | 4.35 | 4.52 | 4.81 | 5.19 | 5.55 | 5.88 | 6.14 | 41.24 |  |
| **Neurological disorders (%)** |  |  |  |  |  |  |  |  |  |
| Alzheimer's disease and other dementias | 0.29 | 0.29 | 0.31 | 0.35 | 0.39 | 0.43 | 0.48 | 64.04 |  |
| Parkinson's disease | 0.05 | 0.05 | 0.06 | 0.06 | 0.07 | 0.08 | 0.09 | 89.05 |  |
| **Mental disorders (%)** |  |  |  |  |  |  |  |  |  |
| Depressive disorders | 2.12 | 2.16 | 2.28 | 2.42 | 2.53 | 2.63 | 2.73 | 28.96 |  |
| Anxiety disorder | 2.06 | 2.12 | 2.20 | 2.01 | 2.06 | 2.40 | 2.43 | 17.75 |  |
| **Substance use disorders (%)** |  |  |  |  |  |  |  |  |  |
| Alcohol use disorders | 0.78 | 0.85 | 0.96 | 1.04 | 1.27 | 1.35 | 1.41 | 80.22 |  |
| Drug use disorders | 0.58 | 0.60 | 0.62 | 0.65 | 0.67 | 0.67 | 0.65 | 10.64 |  |
| **Diabetes and kidney diseases (%)** |  |  |  |  |  |  |  |  |  |
| Diabetes mellitus type 2 | 1.65 | 1.84 | 1.98 | 2.49 | 3.00 | 3.72 | 4.51 | 174.09 |  |
| Chronic kidney disease | 6.11 | 6.44 | 7.03 | 7.85 | 8.70 | 9.55 | 10.36 | 69.58 |  |
| **Musculoskeletal disorders (%)** |  |  |  |  |  |  |  |  |  |
| Osteoarthritis | 2.16 | 2.20 | 2.38 | 2.69 | 3.11 | 3.58 | 4.02 | 85.98 |  |
| **Mongolia** |  |  |  |  |  |  |  |  |  |
| **Neoplasms/Cancer (per 100,000 persons)** |  |  |  |  |  |  |  |  |  |
| Lip and oral cavity cancer | 8.69 | 8.93 | 7.64 | 5.80 | 5.87 | 6.60 | 7.70 | -11.39 |  |
| Colon and rectum cancer | 15.34 | 16.05 | 16.15 | 17.41 | 20.69 | 24.51 | 29.86 | 94.65 |  |
| Stomach cancer | 38.96 | 41.14 | 39.02 | 35.72 | 35.34 | 35.38 | 39.15 | 0.49 |  |
| Pancreatic cancer | 1.13 | 1.29 | 1.46 | 1.85 | 2.34 | 2.78 | 3.34 | 195.58 |  |
| Non-Hodgkin's lymphoma | 0.04 | 0.03 | 0.03 | 0.00 | 0.00 | 0.00 | 0.58 | 1350.00 |  |
| Gallbladder and biliary tract cancer | 2.02 | 2.09 | 2.06 | 2.02 | 2.12 | 2.17 | 2.47 | 22.28 |  |
| Kidney cancer | 2.71 | 2.68 | 3.33 | 5.40 | 7.55 | 8.95 | 10.69 | 294.46 |  |
| Ovarian cancer | 4.29 | 5.33 | 6.57 | 8.55 | 9.66 | 10.60 | 12.05 | 180.89 |  |
| Cervical cancer | 34.12 | 36.78 | 40.62 | 44.66 | 48.42 | 47.36 | 51.02 | 49.53 |  |
| Breast cancer | 32.79 | 32.73 | 37.22 | 46.60 | 55.56 | 59.94 | 71.56 | 118.24 |  |
| Oesophageal cancer | 14.06 | 14.80 | 14.78 | 14.36 | 14.87 | 14.95 | 17.05 | 21.27 |  |
| Tracheal, bronchus and lung cancer | 18.80 | 19.44 | 17.81 | 15.48 | 15.77 | 17.35 | 20.41 | 8.56 |  |
| Prostate cancer | 5.95 | 5.58 | 5.70 | 7.24 | 9.16 | 10.88 | 13.36 | 124.54 |  |
| Thyroid cancer | 2.22 | 2.39 | 2.82 | 4.28 | 5.91 | 6.87 | 8.30 | 273.87 |  |
| Leukaemia | 11.96 | 11.96 | 9.59 | 10.43 | 10.04 | 9.24 | 9.06 | -24.25 |  |
| Multiple myeloma | 0.59 | 0.58 | 0.59 | 0.65 | 0.79 | 0.91 | 1.07 | 81.36 |  |
| **Cardiovascular diseases (%)** |  |  |  |  |  |  |  |  |  |
| Stroke | 0.85 | 0.89 | 0.95 | 0.97 | 1.01 | 1.08 | 1.09 | 28.68 |  |
| Ischemic heart disease | 1.94 | 1.84 | 1.84 | 1.91 | 2.11 | 2.33 | 2.60 | 33.84 |  |
| **Chronic respiratory diseases (%)** |  |  |  |  |  |  |  |  |  |
| Chronic obstructive pulmonary disease | 0.96 | 0.91 | 0.91 | 0.99 | 0.98 | 0.97 | 0.98 | 2.07 |  |
| Asthma | 2.21 | 2.06 | 1.99 | 1.92 | 1.82 | 1.79 | 1.98 | -10.41 |  |
| **Digestive diseases (%)** |  |  |  |  |  |  |  |  |  |
| Gallbladder and biliary diseases | 2.33 | 2.35 | 2.35 | 2.31 | 2.26 | 2.23 | 2.31 | -0.93 |  |
| Upper digestive system diseases | 7.17 | 7.42 | 7.92 | 8.69 | 9.40 | 9.76 | 9.95 | 38.76 |  |
| **Neurological disorders (%)** |  |  |  |  |  |  |  |  |  |
| Alzheimer's disease and other dementias | 0.24 | 0.22 | 0.20 | 0.20 | 0.21 | 0.25 | 0.28 | 17.04 |  |
| Parkinson's disease | 0.03 | 0.03 | 0.03 | 0.03 | 0.03 | 0.03 | 0.04 | 12.61 |  |
| **Mental disorders (%)** |  |  |  |  |  |  |  |  |  |
| Depressive disorders | 3.06 | 3.21 | 3.43 | 3.71 | 3.84 | 3.87 | 3.83 | 25.02 |  |
| Anxiety disorder | 1.86 | 1.92 | 2.03 | 1.87 | 1.87 | 1.84 | 2.09 | 12.29 |  |
| **Substance use disorders (%)** |  |  |  |  |  |  |  |  |  |
| Alcohol use disorders | 1.81 | 2.07 | 2.72 | 3.40 | 4.31 | 4.18 | 4.53 | 149.73 |  |
| Drug use disorders | 0.51 | 0.53 | 0.63 | 0.69 | 0.70 | 0.65 | 0.60 | 18.40 |  |
| **Diabetes and kidney diseases (%)** |  |  |  |  |  |  |  |  |  |
| Diabetes mellitus type 2 | 0.77 | 0.78 | 0.91 | 1.15 | 1.42 | 1.63 | 1.84 | 138.30 |  |
| Chronic kidney disease | 5.45 | 5.65 | 6.00 | 6.75 | 7.55 | 8.00 | 8.32 | 52.67 |  |
| **Musculoskeletal disorders (%)** |  |  |  |  |  |  |  |  |  |
| Osteoarthritis | 2.49 | 2.41 | 2.46 | 2.70 | 3.03 | 3.47 | 3.97 | 59.56 |  |
| **Nauru** |  |  |  |  |  |  |  |  |  |
| **Neoplasms/Cancer (per 100,000 persons)** |  |  |  |  |  |  |  |  |  |
| Lip and oral cavity cancer | 8.61 | 8.56 | 8.48 | 8.34 | 8.66 | 9.52 | 10.49 | 21.84 |  |
| Colon and rectum cancer | 32.56 | 31.19 | 29.44 | 27.00 | 27.79 | 34.15 | 40.81 | 25.34 |  |
| Stomach cancer | 12.29 | 13.20 | 14.33 | 14.26 | 13.63 | 12.68 | 12.83 | 4.39 |  |
| Pancreatic cancer | 1.22 | 1.33 | 1.39 | 1.41 | 1.37 | 1.62 | 1.91 | 56.56 |  |
| Non-Hodgkin's lymphoma | 0.94 | 0.95 | 0.88 | 0.02 | 0.56 | 3.01 | 4.37 | 364.89 |  |
| Gallbladder and biliary tract cancer | 0.73 | 0.74 | 0.77 | 0.74 | 0.72 | 0.70 | 0.73 | 0.00 |  |
| Kidney cancer | 9.13 | 9.43 | 8.84 | 7.90 | 7.56 | 8.82 | 10.43 | 14.24 |  |
| Ovarian cancer | 5.22 | 6.20 | 7.46 | 8.55 | 9.19 | 10.90 | 12.33 | 136.21 |  |
| Cervical cancer | 67.70 | 73.37 | 81.52 | 80.42 | 81.55 | 80.66 | 83.18 | 22.87 |  |
| Breast cancer | 98.99 | 108.22 | 125.86 | 136.40 | 150.94 | 173.05 | 194.82 | 96.81 |  |
| Oesophageal cancer | 2.04 | 2.20 | 2.30 | 2.20 | 2.02 | 1.97 | 2.07 | 1.47 |  |
| Tracheal, bronchus and lung cancer | 15.76 | 15.53 | 15.70 | 15.09 | 15.01 | 15.82 | 17.01 | 7.93 |  |
| Prostate cancer | 20.74 | 21.66 | 21.99 | 20.85 | 20.70 | 23.79 | 28.30 | 36.45 |  |
| Thyroid cancer | 11.19 | 13.00 | 14.85 | 15.11 | 15.76 | 18.22 | 21.02 | 87.85 |  |
| Leukaemia | 42.29 | 44.97 | 48.15 | 47.61 | 44.17 | 37.12 | 34.77 | -17.78 |  |
| Multiple myeloma | 1.34 | 1.36 | 1.43 | 1.40 | 1.40 | 1.45 | 1.58 | 17.91 |  |
| **Cardiovascular diseases (%)** |  |  |  |  |  |  |  |  |  |
| Stroke | 1.07 | 1.09 | 1.15 | 1.13 | 1.12 | 1.11 | 1.12 | 5.18 |  |
| Ischemic heart disease | 0.79 | 0.79 | 0.79 | 0.78 | 0.78 | 0.80 | 0.84 | 6.69 |  |
| **Chronic respiratory diseases (%)** |  |  |  |  |  |  |  |  |  |
| Chronic obstructive pulmonary disease | 1.31 | 1.29 | 1.26 | 1.21 | 1.20 | 1.28 | 1.35 | 2.86 |  |
| Asthma | 3.37 | 2.99 | 2.93 | 2.73 | 2.52 | 2.52 | 3.22 | -4.25 |  |
| **Digestive diseases (%)** |  |  |  |  |  |  |  |  |  |
| Gallbladder and biliary diseases | 0.66 | 0.69 | 0.74 | 0.77 | 0.75 | 0.74 | 0.73 | 10.38 |  |
| Upper digestive system diseases | 3.98 | 4.05 | 4.18 | 4.25 | 4.26 | 4.30 | 4.38 | 10.05 |  |
| **Neurological disorders (%)** |  |  |  |  |  |  |  |  |  |
| Alzheimer's disease and other dementias | 0.10 | 0.10 | 0.09 | 0.09 | 0.08 | 0.08 | 0.09 | -11.24 |  |
| Parkinson's disease | 0.04 | 0.03 | 0.03 | 0.03 | 0.03 | 0.03 | 0.04 | -4.56 |  |
| **Mental disorders (%)** |  |  |  |  |  |  |  |  |  |
| Depressive disorders | 2.37 | 2.42 | 2.49 | 2.55 | 2.57 | 2.60 | 2.65 | 11.97 |  |
| Anxiety disorder | 3.40 | 3.46 | 3.55 | 3.56 | 3.64 | 3.72 | 3.78 | 11.35 |  |
| **Substance use disorders (%)** |  |  |  |  |  |  |  |  |  |
| Alcohol use disorders | 0.64 | 0.65 | 0.66 | 0.66 | 0.66 | 0.68 | 0.70 | 8.84 |  |
| Drug use disorders | 0.70 | 0.72 | 0.74 | 0.75 | 0.75 | 0.76 | 0.78 | 11.20 |  |
| **Diabetes and kidney diseases (%)** |  |  |  |  |  |  |  |  |  |
| Diabetes mellitus type 2 | 3.12 | 3.58 | 4.08 | 4.61 | 5.18 | 5.91 | 6.66 | 113.49 |  |
| Chronic kidney disease | 6.00 | 6.23 | 6.49 | 6.62 | 6.77 | 7.11 | 7.46 | 24.39 |  |
| **Musculoskeletal disorders (%)** |  |  |  |  |  |  |  |  |  |
| Osteoarthritis | 2.03 | 2.05 | 2.13 | 2.20 | 2.23 | 2.37 | 2.54 | 24.95 |  |

N.A, not available.

**Reference**

1. Tian T, Song C, Jiang L, Dai J, Lin Y, Xu X, et al. Hepatitis B virus infection and the risk of cancer among the Chinese population. *Int J Cancer* (2020) 147(11):3075-84. doi:10.1002/ijc.33130

2. Li M, Gan Y, Fan C, Yuan H, Zhang X, Shen Y, et al. Hepatitis B virus and risk of non-Hodgkin lymphoma: An updated meta-analysis of 58 studies. *J Viral Hepat* (2018) 25(8):894-903. doi:10.1111/jvh.12892

3. Geng H, Xing Y, Zhang J, Cao K, Ye M, Wang G, et al. Association between viral infection other than human papillomavirus and risk of esophageal carcinoma: a comprehensive meta-analysis of epidemiological studies. *Arch Virol* (2022) 167(1):1-20. doi:10.1007/s00705-021-05268-8

4. Dalia S, Dunker K, Sokol L, Mhaskar R. Hepatitis B seropositivity and risk of developing multiple myeloma or Hodgkin lymphoma: A meta-analysis of observational studies. *Leuk Res* (2015) 39(12):1325-33. doi:10.1016/j.leukres.2015.09.008

5. Cai C, Zeng J, Wu H, Shi R, Wei M, Gao Y, et al. Association between hepatitis B virus infection and diabetes mellitus: A meta-analysis. *Exp Ther Med* (2015) 10(2):693-8. doi:10.3892/etm.2015.2537

6. Fabrizi F, Cerutti R, Ridruejo E. Hepatitis B virus infection as a risk factor for chronic kidney disease. *Expert Rev Clin Pharmacol* (2019) 12(9):867-74. doi:10.1080/17512433.2019.1657828
